# Prevalence of widowhood across states and union territories in India, 1993–2021: a repeated cross-sectional study

**DOI:** 10.7189/jogh.16.04068

**Published:** 2026-02-28

**Authors:** Avnish Pal, Rockli Kim, S V Subramanian

**Affiliations:** 1 Department of Humanities and Social Sciences, Indian Institute of Technology Tirupati, Tirupati, India; 2 Division of Health Policy and Management, College of Health Science, Korea University, Seoul, South Korea; 3 Interdisciplinary Program in Precision Public Health, Department of Public Health Sciences, Graduate School of Korea University, Seoul, South Korea; 4 Harvard Center for Population and Development Studies, Cambridge, Massachusetts, USA; 5 Department of Social and Behavioral Sciences, Harvard T. H. Chan School of Public Health, Harvard University, Boston, Massachusetts, USA

## Abstract

**Background:**

Globally, widowhood affects 258 million females, with India contributing 56 million individuals and 78% of spousal losses being women. Increased life expectancy and reduced mortality inequalities imply a rise in widowhood. Despite profound personal and societal consequences, granular demographic and temporal insights into widowhood in India are limited. This study provides estimates of widowhood prevalence and headcount disaggregated by age and gender at the sub-national level.

**Methods:**

The complete case records of ever-married persons from five rounds of the National Family Health Survey (NFHS), between 1993 to 2021 were analysed. We estimate widowhood prevalence and headcount by age and gender across states and union territories. Integrated Public Use Microdata Series Population Weight (IPUMS POPWT) methodology was utilised to estimate the headcount. Additionally, Standardized Absolute Change (SAC) was estimated to quantify the change in percentage point of widowhood prevalence.

**Results:**

Widowhood prevalence has remained relatively stable; however, the headcount has increased from 40 819 749 to 77 079 889 between 1993 to 2021 in India. At the state-level, the prevalence of widowhood has declined and converged, while its headcount has increased and diverged. Female widowhood prevalence has consistently remained higher, but a faster decline among females, primarily driven by an increased age at marriage. For instance, among females aged 65 years or above, prevalence of widowhood exceeds 50%. This shifts widowhood towards older ages for females, suggesting feminisation and rectangularisation of widowhood in India. A pronounced North-South divide persisted, influenced by differing sociocultural norms.

**Conclusions:**

Widowhood prevalence is increasingly concentrated among older females across states in India. Given the multiple vulnerabilities faced by widows, it is imperative to gain insight from areas where widows survive for extended period after their spouse's death as well as areas, where widows have shorter lifespan such as the northern regions to meaningfully address their specific vulnerabilities.

On 15 March 2022, the United Nations General Assembly (UNGA) acknowledged the array of challenges faced by widows globally, ranging from discrimination to economic insecurity exacerbated by the COVID-19 pandemic [1]. Globally, the estimated number of widows is approximately 258 million, with South Asia alone accounting for 58 million widows [2]. In India, this issue assumes greater significance, as nearly a decade ago, India was home to 56 million people who lost their spouse, of whom 78% were widows [3]. Over the past three decades, life expectancy has increased significantly and mortality inequalities have reduced. Therefore, it is reasonable to infer a corresponding increase in the number of widows and widowers [4].

Marriage traditionally provides benefits like increased disposable income, access to material resources, emotional support, social status, and companionship, especially for women in patrilineal societies [5–7]. However, the loss of a spouse renders individuals vulnerable, often leading to loneliness, mental distress, declining health, and economic instability as they adjust to living alone within family or in institutions, with females experiencing a disproportionate burden [6,8]. The main challenges associated with widowhood stem from shifts in living arrangements and reduced financial support. Men often experience an increased financial burden due to covering caregiving expenses previously managed by their wives. Additionally, if the wife contributed financially, her death would exacerbate household responsibilities and reduce family income [9,10]. Widows, particularly those lacking financial independence, frequently experience hardships, such as difficulties in securing gainful employment opportunities and authority imbalances within family relationships [9,11]. In patrilineal societies, they also encounter obstacles in claiming their late husband's assets or property, as they are frequently expected to cede authority to male relatives [1,8]. The ‘widowhood effect’, characterised by heightened morbidity and premature mortality following widowhood, is notably pronounced among both men and women around the world [12–14]. Although remarriage can alleviate some of the hardships, it is generally more socially acceptable for men, while it is often regarded as taboo for women [14]. In the evolving demographic and epidemiological landscape of India characterised by declining fertility rates, increased longevity, and rise in non-communicable diseases, there has been increased health care demands and an acceleration of ageing. These changes are transforming the experience of widowhood by amplifying vulnerabilities for both genders. Consequently, widowhood has profound consequences for individuals, families, and society as a whole. The fragmented social security system further exacerbates the plight of the widows, influencing intergenerational transfers as resources are diverted to support widows and widowers, particularly in advanced ages [15,16].

Indian studies frequently underscore vulnerabilities associated with widowhood but often lack precise data on the scale and demographic distribution of affected populations. Without comprehensively understanding the magnitude and disaggregated characteristics of widowhood, it becomes challenging for policymakers to design and implement tailored interventions to effectively address the complex and multifaceted challenges encountered by widows [1,3].

In their seminal study, Chen and Dreze (1992) noted that the impact of widowhood varies significantly across different health care systems, geographical regions, and sociocultural contexts. Their research revealed a clear North-South divide in the incidence of widowhood by the early 1990s, with lower rates observed in North India compared to South India. Further through this influential work, they established that the measure of widowhood prevalence and headcount alone offers critical insights into the challenges and vulnerabilities faced by widows in India.

Building upon these considerations, our study, the first of its kind, presents an age-wise disaggregated analysis of widowhood prevalence by genders across states and union territories (UTs) of India [17]. Our aim is to discern four major patterns, each underpinned by a distinct focus. Age-specific patterns help to capture various stages of life. When combined with gender-specific patterns, these analyses illuminate variations in age at marriage, remarriage rates and the divergent impacts on men and women. Additionally, geographic patterns in widowhood, informed by variations in life expectancy, mean age at marriage, and remarriage rates, collectively elucidate levels of widowhood survival. Lastly, temporal trends provide insights into progress and potential effectiveness of policy interventions. This analysis serves as a critical lens to understanding the association between multiple deprivations, health, and survival outcomes, highlighting how vulnerable populations – especially in low socioeconomic settings – adapt and survive, as reflected in their prevalence rates.

## METHODS

### Data

For this cross-sectional study, we leveraged data from all five waves of the National Family Health Survey (NFHS), conducted in 1992–93, 1998–99, 2005–06, 2015–16, and 2019–21, hereafter referred to by the end year of each survey [18–22]. National Family Health Survey is a part of the Demographic and Health Surveys (DHS) programme and utilises a multistage stratified cluster sampling design (Text S1 in the **Online Supplementary Document**). Population data for calculating headcount were obtained from the Census of India conducted in 1991 and 2001, and projections for 2006, 2016, and 2021 were derived from its published population projections [23,24] (Text S2 in the **Online Supplementary Document**).

The NFHS surveys underwent ethical approval from institutional review boards of ICF International and the International Institute for Population Sciences (IIPS). As per the guidelines of the Harvard Longwood Institutional Review Board, this study does not meet the regulatory definition of research with human subjects and is exempt from ethical review [25].

### Study population

This is a complete case analysis of all ever-married persons. The analytical sample of ever-married persons includes only those aged 13 years and above, as information on marital status was not collected for those aged 12 years or less in the fourth and fifth rounds of NFHS. The final analytical sample included 112 979 males and 134 057 females (NFHS 1), 115 324 males and 136 080 females (NFHS 2), 121 870 males and 145 501 females (NFHS 3), 704 598 males and 829 722 females (NFHS 4), and 723 813 males and 869 214 females (NFHS 5).

### Widowhood

The NFHS survey asks a consistent question to the household questionnaire respondent about marital status of each member aged 13 years or above. Those who were ‘never married’, ‘married but no gauna performed’, or had not reported any marital status were excluded from the study sample. Among the remaining ever-married population, the ‘widowed’ population is identified as per reported marital status. Additionally, women aged 15–49 and men aged 15–54 years living in the households selected for the state module were asked about their own marital status. We use this information to provide an alternative estimate of widowhood prevalence (Text S3 in the **Online Supplementary Document**).

### Constructing comparable states and UTs across NFHS surveys

The spatial configuration of Indian states and Union Territories (UTs) has undergone significant transformations over the past three decades, marked by alterations in the geographic boundaries of ten states/UTs during this period. Consequently, the establishment of a consistent repeated cross-sectional panel for states/UTs across these years is difficult. To address this challenge, we have aligned (and redistributed) stratum in earlier surveys with states according to their contemporary geometry as per a published methodology [26].

### Statistical analysis

We estimated the prevalence of widowhood in India at national and sub-national level across different time periods employing the complex multistage stratified cluster sampling design through survey weights. Additionally, we employed IPUMS POPWT methodology to compute the headcount of widowhood in each state and union territory (Text S2 in the **Online Supplementary Document**) [27]. To capture various life stages as broadly as possible, we analysed it for four distinct age groups: less than 45 years, 45–64 years, 65–74 years, and 75 years or above, disaggregated by gender. We have also calculated the Standardized Absolute Change (SAC) to meaningfully quantify changes in widowhood prevalence, as there is variation in the number of years between the surveys [25]. The measure was computed as follows, where P_t_ is the widowhood prevalence at time *t*, P_t–n_ is the widowhood prevalence *n* years before *t*, and *n* represents the number of years between the two surveys:

*SAC =* (*P_t_ – P_t–n_*)/*n*

A negative SAC value indicates a decline in widowhood prevalence, while a positive rate signifies an increase. Both prevalence and headcount across India were compared with corresponding census data from comparable years. Specifically, we correlated the 1991 census with NFHS 1 (1992–93), the 2001 census with NFHS 2 (1998–99), and the 2011 census with NFHS 4 (2015–16) to ensure robustness of our findings [17–19,21]. State-level dynamics over time are illustrated using a variety of visual and descriptive methods. Our study adheres to the STROBE statement for observational studies, ensuring transparency and rigor in our reporting. We used Stata, version 18.0 (StataCorp 4905 Lakeway Drive College Station, Texas, USA) for our analysis.

## RESULTS

### Sample characteristics

The analytical sample of ever-married persons is described alongside the unweighted percentages of widowhood, which consisted of 112 979 (4.7%) males and 134 057 (13.6%) females in 1993; 115 324 (4.7%) males and 136 080 (13.7%) females in 1999; 121 870 (4.0%) males and 145 501(14.6%) females in 2006; 704 598 (4.4%) males and 829 722 (13.4%) females in 2016; and 723 813 (4.3%) males and 869 214 (13.9%) females in 2021 (**Table 1**). The age- and state-wise distribution of analytical sample, along with unweighted percentage (Table S1–4 in the **Online Supplementary Document**).

**Table 1:** Sample size (n) of ever-married men and women and unweighted percentage (%) of widows/widowers among them in 1993, 1999, 2006, 2016, and 2021, India

	Total				75 years or above				65–74 years				45–64 years				Less than 45 years*			
**Year**	**Male**		**Female**		**Male**		**Female**		**Male**		**Female**		**Male**		**Female**		**Male**		**Female**	
	**n**	**%**	**n**	**%**	**n**	**%**	**n**	**%**	**n**	**%**	**n**	**%**	**n**	**%**	**n**	**%**	**n**	**%**	**n**	**%**
1993	112 979	4.7	134 057	13.6	3621	32.3	3355	81.6	8793	15.8	7442	60.8	33 158	6.1	31 955	26.1	67 407	1.1	91 305	2.9
1999	115 324	4.7	136 080	13.7	3923	30.5	3469	81.7	9323	16.0	8165	58.8	34 173	5.8	32 845	25.3	67 905	1.1	91 601	3.0
2006	121 870	4.0	145 501	14.6	4187	29.1	4268	80.8	9569	14.9	9184	59.3	39 559	4.3	38 505	24.3	68 555	0.8	93 544	3.2
2016	704 598	4.4	829 722	13.4	27 435	29.6	28 118	75.5	63 625	13.4	58 523	50.5	248 972	4.5	253 160	18.7	364 566	0.9	489 921	2.7
2021	723 813	4.3	869 214	13.9	30 080	27.6	30 783	74.1	72 495	12.3	66 677	48.8	268 577	4.2	283 555	18.6	352 661	0.8	488 199	2.7

*The age group of less than 45 includes those aged between 13 and 44.

### All India trends

In India, the prevalence of widowhood among ever-married persons is highest among those aged 75 years or above, followed by 65 to 74 years, 45 to 64 years, and under 45 years. However, the headcount is highest in age cohort 45 to 64 years, followed by 65 to 74 years, 75 years or above, and under 45 years (**Table 2**; Table S5–7 in the **Online Supplementary Document**). Moreover, the prevalence of widowhood has declined, while the headcount has increased among ever-married men and women across age groups between 1993 and 2021.

**Table 2:** Prevalence of widowhood (95% CI) and its headcount (N) among ever-married men and women in 1993, 1999, 2006, 2016, and 2021, India

	75 years or above		65–74 years		45–64 years		Less than 45 years*	
**Year**	**Male**	**Female**	**Male**	**Female**	**Male**	**Female**	**Male**	**Female**
**Prevalence**								
1993	32.3 (30.8, 33.9)	81.5 (80.2, 82.8)	16.7 (15.9, 17.4)	61.1 (60.0, 62.2)	6.7 (6.4, 6.9)	26.6 (26.1, 27.1)	1.2 (1.1, 1.3)	2.8 (2.7, 2.9)
1999	31.0 (29.5, 32.4)	81.9 (80.6, 83.2)	16.0 (15.3, 16.8)	60.1 (59.1, 61.2)	5.9 (5.7, 6.2)	26.4 (26.0, 26.9)	1.1 (1.0, 1.2)	3.1 (3.0, 3.2)
2006	29.4 (28.0, 30.8)	82.1 (81.0, 83.3)	15.3 (14.6, 16.0)	60.4 (59.4, 61.4)	5.0 (4.8, 5.2)	24.3 (23.8, 24.7)	0.9 (0.8, 1.0)	3.1 (3.0, 3.2)
2016	28.2 (27.6, 28.7)	77.5 (77.0, 78.0)	12.6 (12.4, 12.9)	52.3 (51.9, 52.7)	4.1 (4.0, 4.2)	19.3 (19.2, 19.5)	0.8 (0.8, 0.8)	2.8 (2.8, 2.9)
2021	26.3 (25.8, 26.8)	75.3 (74.8, 75.8)	11.6 (11.3, 11.8)	50.1 (49.7, 50.5)	4.0 (3.9, 4.0)	19.1 (18.9, 19.2)	0.7 (0.7, 0.8)	2.7 (2.7, 2.8)
**Headcount**								
1993	1 754 533	4 151 674	2 005 253	6 952 607	3 920 369	14 025 051	2 584 177	5 426 085
1999	2 223 180	6 065 630	2 723 285	10 530 189	4 270 659	17 779 644	3 002 369	7 775 164
2006	2 395 823	7 089 563	2 888 090	12 377 560	4 233 838	19 063 496	2 729 483	8 813 919
2016	3 437 333	10 487 367	3 312 199	14 557 212	4 568 047	20 619 159	2 748 362	9 302 447
2021	3 924 912	12 997 465	3 397 347	15 781 424	5 027 236	23 718 301	2 767 866	9 465 338

*The age group of less than 45 includes those aged between 13 and 44.

### Age and gender dynamics of widowhood

The prevalence and headcount of widowhood is consistently higher among females compared to males across age groups and time periods. Among women, the largest decline in widowhood prevalence occurred in 65 to 74 years age group, which decreased from 61.1% (95% confidence interval (CI) = 60.0, 62.2) in 1993 to 50.1% (95% CI = 49.7, 50.5) in 2021, followed by the 45 to 64 years age group, and then the 75 years or above cohort. While among men, the most significant decline occurred in age group 75 years or above, followed by 65 to 74 years, and 45 to 64 years (**Table 2**; Table S5 in the **Online Supplementary Document**). These trends are confirmed by the negative SAC across all age groups (Table S8 in the **Online Supplementary Document**). Additionally, the rate of increase in headcount is similar for both genders across the age groups, with the highest observed in ages 75 years or above, followed by 65 to 74 years, and then 45 to 64 years (Table S6–7 in the **Online Supplementary Document****)**. Overall, the nationwide numbers from this study closely align with estimates for the same categories reported in Census of India (Table S9 in the **Online Supplementary Document****)**.

There is a slight variation between the original estimates and the sensitivity analysis in prevalence and headcount of widowhood (Table S10–11 in the **Online Supplementary Document**). For instance, in 2021, the prevalence among females aged less than 45 years was 2.7 in the original estimates, while it was 2.8 in the sensitivity analysis. Similarly, the headcount for females aged 45 to 64 years in 2021 was 23 718 301 in the original estimates and 23 860 304 in sensitivity analysis. In 2006, the headcount for females aged less than 45 years was 8 813 919 in original estimates and 8 782 258 in the sensitivity analysis, showcasing the deviations of the original estimates from the sensitivity analysis.

### Geographic variation in temporal patterns

**Figure 1:**
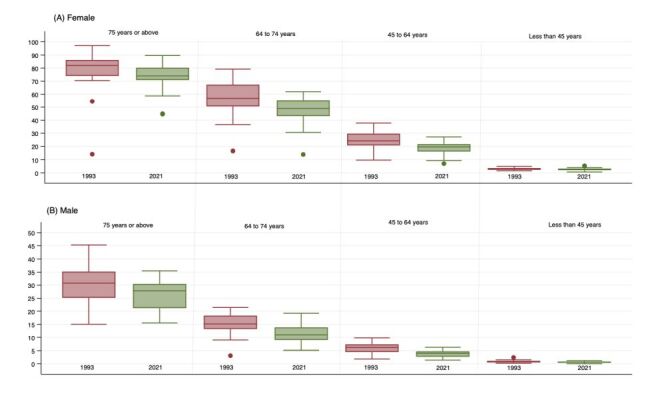
Summary distribution of state/union territory-level prevalence of widowhood among ever-married men and women, 1993, and 2021. The horizontal bar inside the box indicates the median, the lower and upper ends of the boxes are the 25th and 75th percentile, respectively, and represent the IQR. The whiskers indicate data 1.5 times the IQR and the circles indicate outliers. IQR – interquartile range.

**Figure 2:**
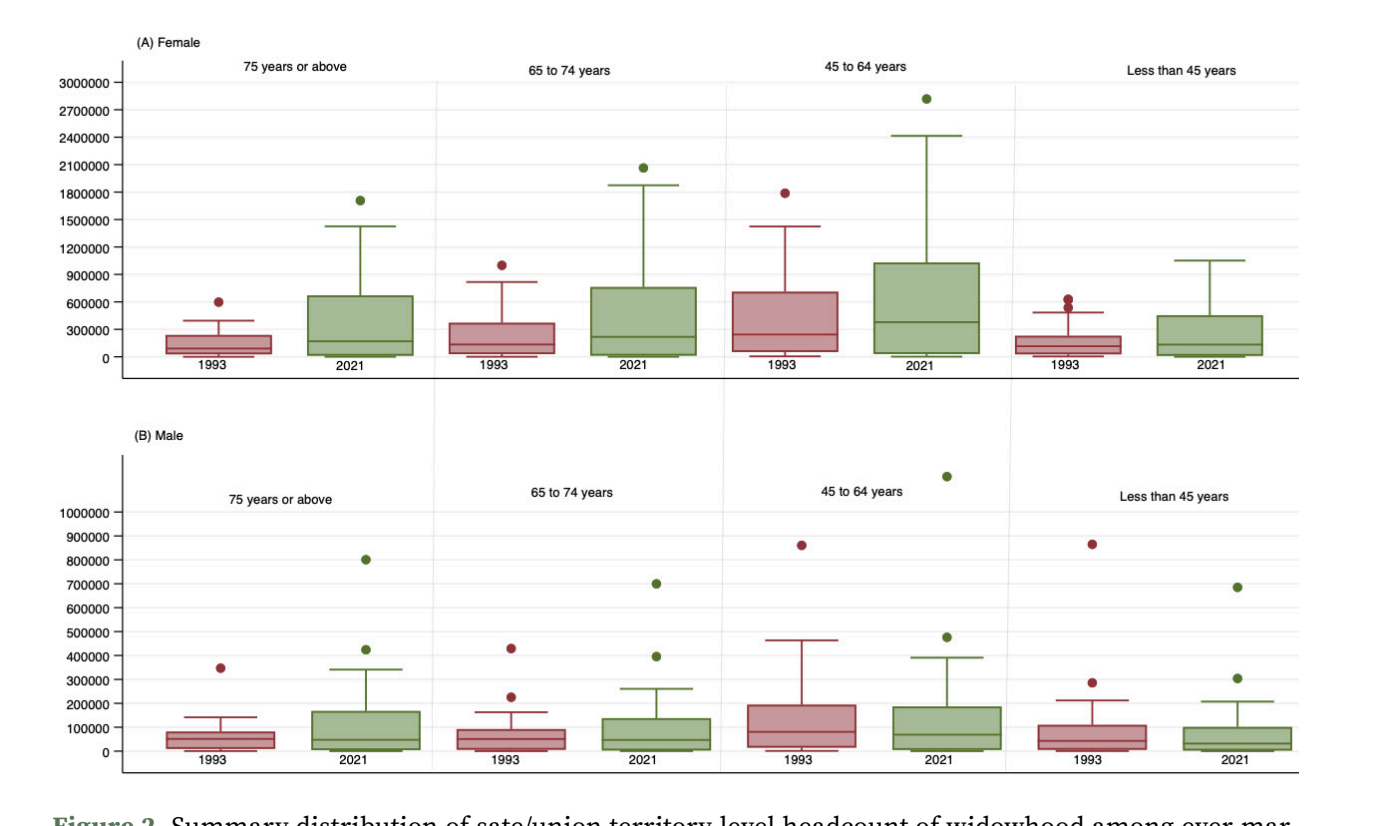
Summary distribution of sate/union territory-level headcount of widowhood among ever-married men and women, 1993, and 2021. The horizontal bar inside the box indicates the median, the lower and upper ends of the boxes are the 25th and 75th percentile, respectively, and represent the IQR. The whiskers indicate data 1.5 times the IQR and the circles indicate outliers. IQR – interquartile range.

**Figure 3:**
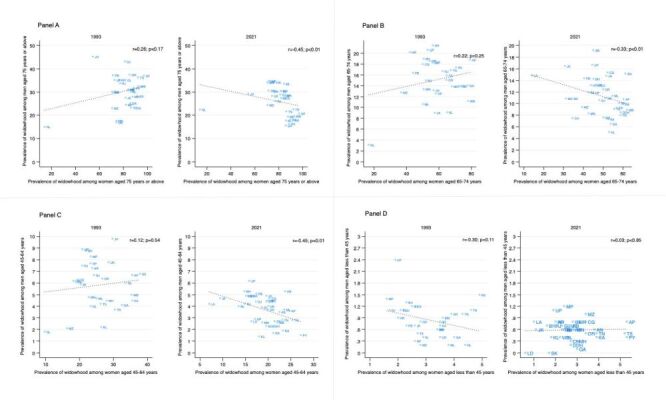
Relationship between prevalence of widowhood among ever-married men and women, 1993, and 2021 across states and union territories of India. **Panel A.** Association between prevalence of widowhood among women and men aged 75 years or above. **Panel B.** Association between prevalence of widowhood among women and men aged 65–74 years. **Panel C.** Association between prevalence of widowhood among women and men aged 45–64 years. **Panel D.** Association between prevalence of widowhood among women and men aged less than 45 years.

We observe a slight convergence in the prevalence of widowhood across states for both men and women of all ages (**Figure 1**). However, there is evidence of divergence of widowhood headcount across states and UTs for both genders (**Figure 2**). In 2021, an inverse correlation emerged between those aged 75 years or above and those aged 45 to 64 years in relation to the ratio of widowhood prevalence amongst males as compared to widowhood prevalence among females. This correlation though weak, was statistically significant (r = −0.45; *P* < 0.01) (**Figure 3**, Panels A–D).

A pronounced North-South divide in widowhood prevalence was evident in 1993, with rates among females conspicuously higher in the southern states and prevalence among males higher in northern states (**Table 3**; Figure S1 in the **Online Supplementary Document**). By 2021, this regional pattern largely persisted as northern states continued to exhibit higher than average widowhood rates among males, and southern states maintained above average widowhood rates for females in three (of four) age groups. Some states that had above-average rates in 1993 fell below national average by 2021. States with consistently below-average rates for both genders are predominately in eastern, western, and northeastern regions, particularly for females aged 65 years or above. However, headcounts have increased across all states (Table S6–7 in the **Online Supplementary Document**). Several states – namely Arunachal Pradesh, Assam, Bihar, Haryana, Jharkhand, Jammu and Kashmir, Madhya Pradesh, Manipur, Meghalaya, Nagaland, Tripura, and Uttarakhand - have shown a worsening trend for either males or females in at least one age group. Notably, Nagaland have shown a worsening trend across all age groups for both genders (Table S8 in the **Online Supplementary Document**).

**Table 3:** Prevalence of widowhood among ever-married men and women in 1993, and 2021, across states and union territories of India

	75 years or above				65 to 74 years				45 to 64 years				Less than 45 years*			
**Location**	**Male**		**Female**		**Male**		**Female**		**Male**		**Female**		**Male**		**Female**	
	**1993**	**2021**	**1993**	**2021**	**1993**	**2021**	**1993**	**2021**	**1993**	**2021**	**1993**	**2021**	**1993**	**2021**	**1993**	**2021**
**India**	32.3	26.3	81.5	75.3	16.7	11.6	61.1	50.1	6.7	4.0	26.6	19.1	1.2	0.7	2.8	2.7
**States**																
Andhra Pradesh	37.1	20.7	97.0	83.4	18.8	10.8	79.2	60.3	6.7	3.6	35.5	24.7	0.8	0.8	3.5	5.3
Arunachal Pradesh	30.0	30.7	71.4	70.9	21.4	19.2	56.2	45.0	7.3	4.9	23.7	16.3	1.0	0.8	3.9	2.0
Assam	28.3	17.9	91.7	77.5	17.4	9.9	70.5	60.1	6.8	3.5	37.8	22.5	1.5	0.6	4.9	2.8
Bihar	30.2	34.1	70.5	73.0	19.0	14.3	50.6	45.1	8.8	5.3	21.9	14.8	1.3	0.7	2.3	1.6
Chhattisgarh	30.8	27.5	83.5	76.5	18.0	15.4	50.0	52.2	6.3	4.7	19.4	20.3	1.2	0.8	2.6	3.4
Goa	25.2	15.5	85.6	83.6	14.1	6.5	77.6	53.7	4.1	1.7	32.5	21.1	0.4	0.1	4.0	3.0
Gujarat	43.2	29.0	81.1	71.2	16.7	10.6	62.3	48.0	6.7	4.4	24.2	17.3	1.1	0.7	2.2	2.3
Haryana	35.3	34.2	76.3	73.2	20.2	13.2	42.0	43.7	6.2	4.5	18.2	19.8	0.9	0.8	2.0	3.0
Himachal Pradesh	31.1	28.5	84.2	76.4	12.6	11.9	58.1	45.0	7.7	2.9	23.2	17.1	0.3	0.8	2.6	2.0
Jharkhand	45.3	29.3	54.5	70.9	13.2	14.8	56.7	51.6	9.8	4.9	29.7	19.6	1.2	0.6	2.5	2.4
Karnataka	31.9	16.4	88.6	79.4	14.0	7.3	72.3	52.6	4.5	1.9	35.5	23.3	0.7	0.4	3.5	3.9
Kerala	30.2	17.7	86.0	81.5	9.1	5.1	65.0	53.0	2.2	1.4	26.4	18.0	0.4	0.4	2.9	1.8
Madhya Pradesh	24.5	30.5	82.0	72.4	18.6	13.2	54.3	46.1	8.3	4.9	23.3	15.6	1.4	1.2	1.6	2.4
Maharashtra	28.1	19.4	87.6	71.7	13.9	7.6	67.9	50.1	4.5	2.3	28.0	19.6	0.6	0.3	3.4	3
Manipur	17.6	30.4	74.0	75.3	13.4	8.3	50.6	42.6	4.8	2.6	22.7	18.7	0.9	0.6	3.6	2.9
Meghalaya	31.2	33.6	84.2	75.1	10.6	11.4	50.0	54.6	4.7	3.7	25.0	23.5	0.2	0.7	3.8	2.6
Mizoram	23.0	24.4	70.5	66.0	12.8	9.4	36.7	39.3	2.1	4.5	16.3	20.3	0.2	1.0	2.8	3.4
Nagaland	15.0	22.4	14.2	44.6	3.1	10.9	16.6	31.2	1.8	4.0	9.7	13.5	0.2	0.6	4.4	2.4
Odisha	37.5	28.5	85.1	74.8	15.1	12.5	64.6	50.0	6.4	3.7	26.7	19.9	0.8	0.5	2.8	3.4
Punjab	37.3	34.6	71.2	68.8	16.4	14.4	44.0	40.4	6.1	5.1	18.9	17.4	0.7	0.7	2.3	2.7
Rajasthan	33.8	26.0	77.2	72.5	15.0	12	52.0	42.5	7.5	4.2	20.8	14.9	1.1	0.7	1.7	1.9
Sikkim	.	23.7	.	72.7	.	10.9	.	34.1	.	4.9	.	15.1	.	0.0	.	1.7
Tamil Nadu	23.2	21.6	86.2	80.6	16.9	9.3	67.3	56.8	6.0	2.8	33.5	23.1	1.0	0.5	4.3	3.9
Telangana	36.3	22.5	92.1	81.1	15.9	9.1	64.8	55.6	4.2	2.8	26.2	25.4	0.5	0.5	2.7	5.2
Tripura	23.0	18.0	89.4	80.0	13.9	8.0	73.3	58.7	3.7	2.7	30.4	21.4	1.1	0.6	4.6	2.3
Uttar Pradesh	35.2	34.9	72.6	70.4	20.6	16.7	53.1	44.5	8.9	6.2	20.8	15.8	2.4	1.1	1.9	1.9
Uttarakhand	16.9	28.4	74.2	76.1	18.4	11.0	56.9	51.1	7.8	4.0	28.5	20.0	0.4	0.8	3.5	2.8
West Bengal	32.6	19.6	92.6	85.2	11.2	8.9	77.9	61.9	5.0	2.6	35.3	22.0	0.6	0.4	3.4	2.2
**Union territories**																
Andaman & Nicobar Islands (UT)	.	28.9	.	84.2	.	15.2	.	61.2	.	5.3	.	23.7	.	0.6	.	3.8
Chandigarh (UT)	.	34.2	.	58.7	.	7.5	.	34.5	.	4.4	.	19.6	.	0.3	.	2.7
Dadra & Nagar Haveli and daman & Diu (UT)	.	25.8	.	71.7	.	8.5	.	58.8	.	2.3	.	20.6	.	0.2	.	2.7
Jammu & Kashmir (UT)	27.6	27.5	81.7	60.4	9.0	16.5	57.5	30.7	5.9	4.7	20.3	9.4	0.7	0.6	2.6	1.1
Ladakh (UT)	.	35.5	.	45.0	.	14.9	.	14.0	.	4.2	.	7.0	.	0.8	.	1.0
Lakshadweep (UT)	.	15.5	.	88.1	.	13.2		48.1	.	2.7	.	16.3	.	0.0	.	0.6
NCT of Delhi (UT)	35.2	30.5	81.7	68.2	18.3	13.3	56.0	46.8	4.0	3.9	23.3	18.6	1.1	0.4	2.1	2.4
Puducherry (UT)	.	28.2	.	89.6	.	10.1		58.0	.	1.5	.	27.2	.	0.4	.	5.3

*The age group of less than 45 includes those aged between 13 and 44.

Meanwhile analysis of data from 1999, 2006, and 2016, shows a consistent decline in widowhood prevalence among men across most states for all four age groups (Table S12–15 in the **Online Supplementary Document**). However, few states experienced an increase between 1999 and 2006 and a subsequent decline by 2016, while others observed a steady increase between 1999 and 2016. For instance, for men in the age group 75 years or above, widowhood prevalence in Madhya Pradesh has increased from 28% in 1999 to 35.9% in 2006 before declining to 29.5% in 2016.

## DISCUSSION

This analysis reveals a paradox: while prevalence of widowhood has declined and converged, its headcount has increased and diverged among various age groups across Indian states and union territories. This paradox can be attributed to population growth, increased longevity, and reduced variability in lifespan for both genders [4,28]. The reduction of gender disparities in widowhood prevalence across age-groups is primarily driven by rapid decline in female widowhood rates. The evidence suggests that increased age at marriage is the primary factor, supported by the widening gap across age groups among females and a contrasting narrowing gap among males [29]. This trend is further substantiated by an increase in higher remarriage rate at young ages and lower remarriage at older ages, and higher survival rates of females in advanced ages [13].

Men tend to experience more pronounced effects of widowhood at older ages, often leading to pre-mature mortality post-widowhood. These sociodemographic changes are increasingly shifting widowhood prevalence towards older ages for females, leading to feminisation and rectangularisation of widowhood in India. However, some states deviate from these overall trends. For instance, heightened feminisation of widowhood is observed in the northeastern states without significant rectangularisation. This may imply lower life expectancy rather than absence of widowhood in later life.

In southern states, high prevalence and headcount of widows can be attributed to sharp decline in fertility, increased longevity, higher sex ratio, and an improved and easily accessible health care system. Conversely, northern states have high prevalence and headcount of widowers, a pattern influenced by strict conservative and inegalitarian gender norms such as purdah, patrilocality, seclusion, and patrilineal inheritance. These views and practices hinder women’s participation in gainful employment opportunities, thereby inhibiting their meaningful contribution in household economy. This societal framework leads to further isolation and neglect of widows especially in older ages, eventually leading to pre-mature mortality of widows. In contrast, these restrictive gender norms are less severe in southern India, where social integration and broader opportunities are more common [9,30,31].

Previous research on widowhood has primarily focused on either the social challenges faced by widows, often examined qualitatively or on the health and mortality impacts of widowhood [5,7,9,28,29]. Although some previous studies provide estimates of widowhood prevalence when assessing its effects, they generally lack the depth required to understand how widowhood spreads and where vulnerable populations are located today [6,11,28,32]. The findings by Jain et al. (2022), showing 40–60% of widows aged 65 to 74-year age group closely aligns with our findings of 59–60% validating the reliability of our research. Similarly, our comparison with census data reveals comparable proportions of currently widowed individuals among ever-married men and women (Table S9 in the **Online Supplementary Document**).

Given the evolving socioeconomic and demographic milieu of the Indian society, declining fertility, rapid ageing, changing economic system, less reliance on traditional sources of income by families, and the increased burden of widowhood at older ages are reshaping the experience of widowhood. There is pressing need to create meaningful employment opportunities for widows in rural areas, as evidence indicates that employment rate is higher among widowed females before age 52 than the married females, but is limited to permanent or salaried jobs [7,10,11,31]. At later ages, family is expected to provide adequate support when their functional and intrinsic capacities are impaired. The changing and eroding traditional joint families and reduced social security and cohesion among different generations are likely to overwhelm the health care systems. In the absence of a partner, the state support and institutional care becomes even more critical. Given weak enforcement of inheritance of late husband’s property for widows, fragmented and lower reach of social security amplifies the plight of the widows in India [33]. Despite the increasing criticality of these issues, India’s support policies for widows remain limited. The Indira Gandhi National Widow Pension Scheme (IGNWPS), introduced in 2009, provides a monthly pension of INR 300 (approximately 6 USD) to widows aged 40 to 60 years living below the poverty line (BPL), with state governments supplementing this based on available resources. Beyond this, there are no dedicated national programmes solely targeting widows, although broader schemes like Antyodaya Yojana and Annapurna Yojana indirectly support them by providing subsidised food for BPL families headed by widows. Some states, such as Rajasthan and Uttar Pradesh, offer additional assistance via programmes like Palanhar, which supports widows’ children, and the Rani Lakshmi Bai Pension Scheme, which aids destitute women. However, while a few programmes provide educational scholarships for children, a comprehensive national framework remains elusive.

The present findings necessitate careful consideration in light of several data-related limitations. First, widowhood status is reported by the household head. While significant misreporting is unlikely, recall bias cannot be entirely ruled out. However, the sensitivity analysis using self-reported widowhood status for reproductive-age women did not substantially alter the estimates. Second, NFHS 5 was conducted partly during the COVID-19 pandemic, which could have affected mortality rates differently across age groups and genders, potentially influencing geographic distribution of widowhood prevalence. However, the consistency of trends with previous rounds, such as NFHS-4, supports the reliability of our estimates. Third, because age data are provided by household heads, there is inherent uncertainty, particularly in age-specific widowhood estimates among older populations. Fourth, given the cross-sectional nature of the survey, widowhood status reflects only surviving spouses, potentially leading to underreporting if individuals die shortly after their spouse’s death. This limitation, however, has a limited impact on identifying regions with high existing widowhood rates. Finally, widowhood is a cumulative process: as widows age, their numbers naturally increase because prior widows remain in the population. While this complicates the assessment of changes in widowhood risk, it does not undermine our goal of identifying areas with high prevalence and rising burdens.

## CONCLUSIONS

Over time prevalence of widowhood has declined while the headcount has increased across the states and union territories, with increasingly concentrated in older age groups. Given India’s rapid population ageing and declining fertility, the well-being of widows, particularly in later ages, necessitates immediate and comprehensive attention. Their reliance on fragmented social security, limited medical care, and geriatric services can exacerbate their vulnerability. Failure to sufficiently invest in proper and reliable social security and affordable medical and geriatric health care will not only comprimise the well-being of the current and future widows but also deterimentally impact intergenerational equity and societal welfare. There is pressing need to create employment opportunities, strengthen implementation, expand support, and adopt a geographically nuanced and politically sensitive approach to address the multifaceted challenge faced by the widows across India.

## Additional material


Online Supplementary Document
