# Supplementary material for: Prevalence of widowhood across states and union territories in India, 1993–2021: a repeated cross-sectional study
Source: J Glob Health. 2026 Feb 28;16:04068. doi: 10.7189/jogh.16.04068 (PMC12926678; doi:10.7189/jogh.16.04068)
Supplement: Online Supplementary Document [file jogh-16-04068-s001.pdf]

Supplement to: Pal A, Kim R, Subramanian S. Prevalence of widowhood across states and union territories in India, 1993–2021: a repeated cross-sectional study. J Glob Health. 2026;16:04068.

Table of Contents

Table/Figure/Method	Title	Page
Table S1	Sample size (n) of ever-married men and women aged 75 years or above and unweighted percentage (%) of widows/widowers among them in 2021, 2016, 2006, 1999, and 1993 across states and union territories of India.	1
Table S2	Sample size (n) of ever-married men and women aged 65 to 74 years and unweighted percentage (%) of widows/widowers among them in 2021, 2016, 2006, 1999, and 1993 across states and union territories of India.	2
Table S3	Sample size (n) of ever-married men and women aged 45 to 64 years and unweighted percentage (%) of widows/widowers among them in 2021, 2016, 2006, 1999, and 1993 across states and union territories of India.	3
Table S5	Sample size (n) of ever-married men and women aged less than 45 years and unweighted percentage (%) of widows/widowers among them in 2021, 2016, 2006, 1999, and 1993 across states and union territories of India.	4
Table S5	Prevalence of widowhood (95% CI) among ever-married men and women in 2021 and 1993 across states and union territories of India.	5
Table S6	Headcount (N) of widowhood among ever-married men and women in 1993, and 2021, across states and union territories of India.	6
Table S7	Headcount (N) of widows/widowers among ever-married men and women by age groups in 2016, 2006, and 1999 across states and union territories of India.	7
Table S8	Standardised Absolute Change (SAC) in widowhood prevalence for men and women for ages 75 or above, 65 to 74 years, 45 to 64 years, and less than 45 years between 1993 and 2021, for all states and union territories of India.	8
Table S9	Comparison of widowhood prevalence and headcount for 1991, 2001, and 2011 census rounds with 1992-93, 1998-99, and 2015-16 rounds of NFHS.	9
Table S10	Prevalence of widowhood (95% CI), widowhood* and their Headcount (N) among ever-married men and women in 2021, 2016, 2006, 1999, and 1993, India.	10
Table S11	Sample size and weighted percentage of ever married self-reported widowhood (widowhood*) and as reported by the head of the household (widowhood), in 2021, 2016, 2006,1999, and 1993 India.	11
Table S12	Prevalence (and 95% CI) of widowhood among ever-married men and women aged 75 years or above in 2016, 2006, and 1999 across states and union territories of India.	12

Table S13	Prevalence (and 95% CI) of widowhood among ever-married men and women aged 65 to 74 years in 2016, 2006, and 1999 across states and union territories of India.	13
Table S14	Prevalence (and 95% CI) of widowhood among ever-married men and women aged 45 to 64 years in 2016, 2006, and 1999 across states and union territories of India.	14
Table S15	Prevalence (and 95% CI) of widowhood among ever-married men and women aged less than 45 years in 2016, 2006, and 1999 across states and union territories of India.	15
Figure S1	Summary distribution of State/Union Territories- level prevalence of widowhood among ever married men and women 2016, 2006, 1999.	16
Figure S2	Relationship between Prevalence of widowhood (%) and its headcount (N) among ever-married women across states and union territories.	17
Text S1	National Family Health Survey (NFHS): summary of survey design.	18
Text S2	Methodology used for calculating the population headcount of widowhood.	19-20
Text S3 (A)	Construction of widowhood Variables	200
Text S3 (B)	The sensitivity analysis	21
Text S4	STATA codes to estimate Widowhood Prevalence and Headcount across States and Union Territories of India, 1993 to 2021.	22-38
Text S5	Explanation of authorship change statement	39

Table S1: Sample size (n) of ever-married men and women aged 75 years or above and unweighted percentage (%) of widows/widowers among them in 2021, 2016, 2006, 1999, and 1993 across states and union territories of India.

	2021				2016				2006				1999				1993			
	Male		female		Male		Female		Male		Female		Male		Female		Male		Female	
	n	%	n	%	n	%	n	%	n	%	n	%	n	%	n	%	n	%	n	%
India	30,080	27.6	30,783	74.1	27,435	29.6	28,118	75.5	4,187	29.1	4,268	80.8	3,923	30.5	3,469	81.7	3,621	32.3	3,355	81.6
States																				
Andhra Pradesh	392	21.2	398	84.2	375	24.3	449	86.9	173	26.6	204	84.8	81	34.6	72	93.1	70	37.1	67	97.0
Arunachal Pradesh	626	34.7	449	70.6	308	30.8	238	66.4	45	31.1	30	66.7	32	37.5	24	83.3	30	30.0	21	71.4
Assam	1,081	19.9	910	78.1	846	24.9	787	85.0	131	28.2	111	91.0	94	13.8	80	85.0	147	27.2	90	90.0
Bihar	1,783	33.6	1,574	72.1	1,917	33.2	1,620	70.3	106	31.1	127	81.1	190	35.8	143	65.7	179	32.4	175	69.1
Chhattisgarh	838	28.6	868	75.9	592	31.8	719	81.2	84	33.3	76	82.9	30	30.0	38	89.5	34	32.4	31	83.9
Goa	89	16.9	107	83.2	67	22.4	98	81.6	104	23.1	153	78.4	60	21.7	85	90.6	111	25.2	146	85.6
Gujarat	1,349	28.2	1,689	70.7	873	28.3	1,057	73.4	75	34.7	118	87.3	143	39.9	158	79.8	141	43.3	170	81.2
Haryana	925	33.8	1,058	72.2	777	32.7	688	66.9	114	36.8	111	69.4	127	35.4	104	77.9	188	34.6	117	77.8
Himachal Pradesh	686	27.1	858	75.2	687	30.9	790	76.2	131	34.4	137	80.3	176	30.1	155	82.6	168	31.6	169	84.6
Jharkhand	863	28.9	784	71.8	1,026	31.5	1,009	78.3	74	31.1	89	78.7	39	41.0	22	77.3	30	43.3	13	53.9
Karnataka	1,356	16.6	1,563	80.1	1,061	21.3	1,280	83.8	212	24.1	213	87.8	192	22.9	199	87.4	163	31.9	193	88.6
Kerala	784	17.6	1,163	81.4	611	19.0	875	79.4	154	27.9	229	89.5	166	22.3	197	83.3	192	30.2	230	86.1
Madhya Pradesh	1,968	31.3	2,058	72.4	2,251	29.5	2,549	73.2	201	33.8	197	77.7	298	28.2	227	79.3	189	27.5	193	82.9
Maharashtra	1,928	19.6	1,978	74.5	1,519	24.0	1,557	78.9	328	25.3	323	85.8	200	31.0	208	85.6	153	28.1	130	87.7
Manipur	461	30.4	413	72.9	656	29.6	667	71.1	190	30.0	169	67.5	70	25.7	71	91.6	34	17.7	54	74.1
Meghalaya	155	26.5	193	74.1	138	27.5	187	74.3	39	20.5	56	87.5	30	43.3	28	85.7	16	31.3	19	84.2
Mizoram	324	26.5	291	61.5	525	27.2	486	64.2	57	26.3	71	70.4	53	32.1	55	65.5	26	23.1	34	70.6
Nagaland	637	23.2	394	49.2	701	20.7	517	60.0	158	19.0	138	65.2	39	23.1	37	78.4	20	15.0	7	14.3
Odisha	1,529	28.4	1,404	75.5	1,526	27.5	1,460	78.4	180	28.3	141	76.6	211	29.4	185	84.9	177	37.9	131	85.5
Punjab	1,243	35.2	1,172	67.7	933	40.8	920	70.9	175	33.7	145	73.1	172	30.8	136	72.8	198	37.4	160	71.3
Rajasthan	1,514	25.5	1,819	73.0	1,654	29.0	1,919	75.5	109	29.4	109	82.6	261	31.4	243	78.6	189	33.9	176	77.3
Sikkim	164	29.9	119	67.2	203	44.8	147	72.1	56	39.3	47	66.0	38	42.1	27	81.5	.	.	.	.
Tamil Nadu	1,348	23.1	1,471	81.0	994	22.2	946	75.2	221	28.5	242	86.8	195	28.2	165	90.9	159	23.3	138	86.2
Telangana	1,085	21.5	1,201	81.4	318	21.1	342	79.5	.	.	.	.	55	10.9	45	77.8	55	36.4	38	92.1
Tripura	310	21.3	340	81.2	168	22.6	207	88.4	75	17.3	56	87.5	73	21.9	59	84.8	65	23.1	85	89.4
Uttar Pradesh	3,694	36.0	3,487	69.8	3,775	37.8	3,707	73.6	439	36.7	390	73.9	416	40.1	299	74.9	403	36.5	313	71.9
Uttarakhand	671	29.1	814	79.0	693	27.7	786	78.8	93	28.0	115	80.9	47	21.3	41	82.9	76	26.3	74	79.7
West Bengal	714	20.2	740	85.4	646	22.6	702	86.9	239	20.9	267	90.3	186	21.0	165	90.9	160	33.8	157	91.7
Union Territories																				
Andaman & Nicobar Islands (UT)	102	29.4	88	81.8	96	29.2	67	73.1	.	.	.	.	.	.	.	.	.	.	.	.
Chandigarh (UT)	55	30.9	38	57.9	44	29.6	39	66.7	.	.	.	.	.	.	.	.	.	.	.	.
Dadra & Nagar Haveli and daman & Diu (UT)	69	24.6	114	76.3	45	33.3	67	83.6	.	.	.	.	.	.	.	.	.	.	.	.
Jammu & Kashmir (UT)	748	26.6	593	59.2	952	32.8	788	70.1	135	25.2	111	81.1	154	35.7	120	76.7	163	31.3	142	81.7
Ladakh (UT)	70	35.7	36	44.4	104	46.2	73	56.2	.	.	.	.	.	.	.	.	.	.	.	.
Lakshadweep (UT)	42	16.7	52	88.5	39	15.4	38	76.3	.	.	.	.	.	.	.	.	.	.	.	.
NCT of Delhi (UT)	311	30.9	322	68.3	183	32.2	166	76.5	89	38.2	93	85.0	95	37.9	81	84.0	85	35.3	82	81.7
Puducherry (UT)	166	23.5	225	85.3	132	15.2	171	81.3	.	.	.	.	.	.	.	.	.	.	.	.

Table S2: Sample size (n) of ever-married men and women aged 65 to 74 years and unweighted percentage (%) of widows/widowers among them in 2021, 2016, 2006, 1999, and 1993 across states and union territories of India.

	2021				2016				2006				1999				1993			
	Male		Female		Male		Female		Male		Female		Male		Female		Male		Female	
	n	%	n	%	n	%	n	%	n	%	n	%	n	%	n	%	n	%	n	%
India	72,495	12.3	66,677	48.8	63,625	13.4	58,523	50.5	9,569	14.9	9,184	59.3	9,323	16.0	8,165	58.8	8,793	15.8	7,442	60.8
States																				
Andhra Pradesh	1,340	11.2	1,168	60.6	1,188	11.6	1,130	64.9	509	14.2	487	66.5	207	19.8	180	72.2	.	.	.	.
Arunachal Pradesh	1,412	21.2	1,095	45.3	789	16.9	536	46.5	81	24.7	62	51.6	103	30.1	64	57.8	56	21.4	32	56.3
Assam	2,589	10.9	2,121	59.1	1,964	12.1	1,697	65.3	245	12.7	226	69.5	211	10.0	142	73.9	239	14.6	208	71.2
Bihar	4,819	14.6	3,589	44.6	4,746	14.6	3,743	45.1	302	15.2	268	61.6	528	17.8	373	49.6	481	18.5	372	48.9
Chhattisgarh	2,364	16.0	2,237	53.3	1,720	18.4	1,768	59.5	266	19.6	270	64.4	109	16.5	108	60.2	108	18.5	85	51.8
Goa	212	7.1	241	52.3	159	10.1	181	62.4	272	14.0	323	64.1	116	9.5	172	73.3	269	14.1	371	77.6
Gujarat	3,455	10.8	3,575	48.1	2,045	13.8	2,229	49.7	272	18.8	263	60.1	323	19.5	345	60.3	364	16.8	343	62.4
Haryana	2,278	12.7	2,270	43.7	1,908	13.5	1,689	40.6	223	19.7	212	48.6	367	21.5	293	45.7	359	20.6	318	42.5
Himachal Pradesh	1,280	11.4	1,258	43.6	1,049	11.3	1,110	47.4	256	14.1	233	60.1	362	15.8	320	54.4	325	12.6	248	59.7
Jharkhand	2,370	15.1	1,995	52.8	2,710	15.6	2,346	55.2	219	14.2	166	62.7	108	19.4	70	61.4	40	12.5	36	55.6
Karnataka	3,609	7.5	3,562	53.6	2,713	9.7	2,775	58.5	435	10.6	494	69.8	392	10.0	434	74.7	433	14.1	391	72.4
Kerala	1,710	5.0	1,939	52.1	1,381	7.4	1,444	52.9	312	6.1	396	68.7	293	9.2	344	61.1	436	9.2	481	65.1
Madhya Pradesh	4,805	13.3	4,272	46.1	5,249	14.4	4,934	49.3	517	15.1	482	54.8	653	16.4	570	52.3	512	18.0	400	56.5
Maharashtra	4,722	7.8	4,957	49.7	3,952	7.9	4,022	50.9	789	11.4	809	56.7	556	12.2	545	64.0	437	14.0	399	67.9
Manipur	866	9.1	868	42.4	1,172	10.1	1,129	45.8	330	12.7	288	49.3	155	16.8	140	60.7	104	13.5	75	50.7
Meghalaya	417	14.4	405	55.8	366	15.9	307	56.4	100	19.0	104	59.6	97	8.3	66	62.1	47	10.6	34	50.0
Mizoram	636	11.5	571	36.1	953	10.9	893	37.6	113	13.3	103	42.7	105	13.3	106	54.7	70	12.9	68	36.8
Nagaland	839	11.7	652	34.2	1,003	9.3	797	35.3	231	7.4	166	38.6	96	10.4	67	41.8	32	3.1	18	16.7
Odisha	3,090	12.3	2,889	51.0	3,258	13.4	2,897	51.1	359	13.4	372	62.1	552	15.8	435	60.9	493	15.0	385	64.9
Punjab	2,833	14.2	2,620	40.3	2,226	17.4	1,965	41.7	349	15.2	344	44.8	495	16.2	385	43.6	443	16.5	313	44.1
Rajasthan	3,924	12.2	3,614	42.8	3,883	11.3	3,553	45.4	303	13.5	267	50.9	611	18.0	572	59.4	546	15.0	394	52.0
Sikkim	287	14.6	233	39.1	368	21.5	289	45.0	140	20.7	122	55.7	97	24.7	82	56.1	.	.	.	.
Tamil Nadu	3,214	9.6	3,214	57.2	2,493	9.0	2,280	54.1	508	15.6	498	69.1	478	15.3	496	68.2	402	16.9	361	67.3
Telangana	3,030	8.8	2,941	56.8	894	10.4	849	58.0	.	.	.	.	142	11.3	103	63.1	169	16.0	131	64.9
Tripura	625	9.3	540	59.4	353	11.9	346	64.2	119	12.6	143	73.4	112	10.7	98	65.3	122	13.9	90	73.3
Uttar Pradesh	8,385	16.8	7,188	44.1	8,499	18.9	7,695	48.9	1,041	22.8	921	53.3	897	21.0	714	49.9	961	20.7	768	53.1
Uttarakhand	1,460	11.2	1,559	54.1	1,624	12.3	1,549	54.0	279	14.3	239	58.2	104	12.5	100	57.0	177	18.1	156	60.3
West Bengal	1,782	8.8	1,575	61.8	1,413	10.1	1,357	64.2	525	10.7	506	69.6	474	12.5	397	70.0	370	10.8	306	77.1
Union Territories																				
Andaman & Nicobar Islands (UT)	240	16.3	204	57.4	.	.	.	.	.	.	.	.	.	.	.	.	.	.	.	.
Chandigarh (UT)	76	7.9	83	34.9	69	11.6	77	42.9	.	.	.	.	.	.	.	.	.	.	.	.
Dadra & Nagar Haveli and daman & Diu (UT)	185	8.7	276	57.3	162	12.4	175	63.4	.	.	.	.	.	.	.	.	.	.	.	.
Jammu & Kashmir (UT)	2,130	16.4	1,502	31.2	2,010	13.7	1,587	41.4	266	18.8	213	47.4	337	19.0	225	53.8	331	8.8	258	58.5
Ladakh (UT)	196	15.8	135	14.1	207	17.9	145	36.6	.	.	.	.	.	.	.	.	.	.	.	.
Lakshadweep (UT)	138	13.0	117	48.7	93	5.4	105	60.0	.	.	.	.	.	.	.	.	.	.	.	.
NCT of Delhi (UT)	781	13.7	783	46.0	515	13.6	424	41.0	208	13.0	207	51.2	243	12.8	219	46.6	223	18.4	189	56.1
Puducherry (UT)	396	8.3	429	57.1	299	8.4	334	60.5	.	.	.	.	.	.	.	.	.	.	.	.

Table S3: Sample size (n) of ever-married men and women aged 45 to 64 years and unweighted percentage (%) of widows/widowers among them in 2021, 2016, 2006, 1999, and 1993 across states and union territories of India.

	2021				2016				2006				1999				1993			
	Male		Female		Male		Female		Male		Female		Male		Female		Male		Female	
	n	%	n	%	n	%	n	%	n	%	n	%	n	%	n	%	n	%	n	%
India	268,577	4.2	283,555	18.6	248,972	4.5	253,160	18.7	39,559	4.3	38,505	24.3	34,173	5.8	32,845	25.3	33,158	6.1	31,955	26.1
States																				
Andhra Pradesh	4,540	3.6	5,174	24.9	4,045	3.7	4,439	26.0	2,221	3.5	2,314	29.0	862	6.2	885	35.8	951	6.7	1,020	35.6
Arunachal Pradesh	7,760	5.1	6,937	16.2	5,928	5.6	4,841	18.3	520	7.3	386	25.4	421	15.2	340	23.2	338	7.4	257	23.7
Assam	12,518	3.8	11,819	22.4	9,724	4.3	9,320	26.0	1,203	4.5	1,029	29.7	1,183	3.7	949	31.7	1,101	5.6	882	37.2
Bihar	13,153	5.4	15,167	14.6	14,092	5.4	15,595	15.2	1,110	6.5	1,118	21.6	1,937	7.9	1,676	20.3	1,672	8.7	1,568	22.0
Chhattisgarh	10,335	5.2	10,787	21.3	8,195	6.0	8,260	21.7	1,129	5.8	1,068	25.6	346	8.7	331	24.2	418	7.4	394	22.6
Goa	861	2.2	967	21.1	673	2.7	696	25.9	1,188	2.4	1,351	28.4	603	5.5	680	32.9	1,548	4.1	1,525	32.5
Gujarat	13,760	4.6	14,255	17.4	9,551	4.6	9,728	17.4	1,175	5.0	1,123	22.5	1,495	6.5	1,362	24.8	1,456	6.8	1,388	24.2
Haryana	8,142	4.4	8,546	19.5	7,820	5.5	7,836	16.6	841	4.0	807	22.1	1,019	4.6	968	16.9	899	6.0	880	18.4
Himachal Pradesh	4,762	3.4	5,025	16.9	3,914	2.9	4,333	19.3	1,065	3.8	1,049	20.4	1,252	5.0	1,293	23.9	1,171	6.3	1,145	23.0
Jharkhand	8,966	5.2	9,642	19.6	10,376	5.7	10,791	20.8	936	5.3	920	26.1	618	6.5	495	25.5	211	8.5	202	28.2
Karnataka	11,654	2.0	12,852	23.3	10,152	2.7	10,577	26.3	2,019	2.5	2,025	31.2	1,773	4.0	1,637	30.9	1,626	4.6	1,650	35.5
Kerala	5,782	1.5	6,751	18.0	6,001	1.8	6,554	16.8	1,251	1.4	1,378	23.6	1,185	2.2	1,225	25.9	1,656	2.3	1,726	26.4
Madhya Pradesh	19,732	4.9	19,989	15.6	21,751	5.1	21,259	16.6	1,946	5.3	1,856	19.6	1,950	7.6	1,894	22.0	1,831	8.3	1,691	22.9
Maharashtra	13,933	2.4	14,830	18.9	11,571	2.5	11,969	19.2	3,257	2.8	3,171	23.6	2,175	3.5	2,070	28.7	1,523	4.6	1,537	28.0
Manipur	3,085	3.7	3,337	18.1	4,776	3.6	4,954	17.6	1,341	3.0	1,374	18.1	571	3.3	601	20.1	392	4.9	405	22.7
Meghalaya	3,185	3.7	3,260	21.7	2,656	4.1	2,615	22.5	576	4.3	558	31.5	342	4.4	373	29.8	314	4.8	264	25.0
Mizoram	2,751	4.6	2,870	19.4	4,300	4.0	4,353	16.3	521	3.3	468	14.7	441	5.2	423	21.5	366	2.2	337	16.3
Nagaland	3,988	4.0	3,841	13.2	3,888	2.9	3,801	15.8	1,164	4.2	936	19.0	317	4.4	273	23.8	381	1.8	237	9.7
Odisha	11,043	4.0	11,616	20.1	12,646	4.3	12,343	17.9	1,434	5.0	1,358	25.2	1,736	6.5	1,701	23.3	1,708	6.2	1,656	27.2
Punjab	8,655	4.9	9,414	17.1	7,775	5.3	7,975	16.1	1,203	5.1	1,120	15.6	1,115	5.5	1,181	17.4	1,213	6.1	1,191	19.0
Rajasthan	14,221	4.4	14,839	15.0	1,4612	4.6	15,282	15.1	1,194	6.1	1,191	22.1	2,480	7.2	2,450	22.0	2,111	7.5	2,067	20.9
Sikkim	1,328	6.5	1,173	15.9	1,804	7.9	1,558	13.5	611	7.4	528	22.4	418	7.9	320	20.0	.	.	.	.
Tamil Nadu	10,793	3.1	12,905	23.5	10,911	2.7	11,031	21.7	2,056	3.1	2,273	30.9	1,771	5.0	1,926	32.7	1,527	6.0	1,635	33.6
Telangana	9,986	2.9	11,676	25.8	2,627	3.8	3,061	25.0	.	.	.	.	502	4.4	528	32.4	589	4.2	587	26.2
Tripura	2,803	3.0	2,757	21.2	1,753	1.8	1,711	22.7	512	2.0	470	31.7	434	3.9	441	31.8	401	3.7	407	30.5
Uttar Pradesh	30,192	6.2	31,929	15.5	30,703	6.6	31,514	16.6	3,654	7.2	3,542	21.0	2,865	10.0	2,781	21.6	3,382	9.4	3,198	20.5
Uttarakhand	4,845	3.7	5,719	21.8	5,611	4.4	6,352	22.5	988	4.3	1,052	26.1	421	5.0	416	25.7	624	6.9	598	30.3
West Bengal	7,718	2.9	7,757	22.5	6,658	2.9	6,303	22.7	2,309	3.3	2,152	29.0	1,764	3.0	1,664	31.3	1,535	5.0	1,448	35.5
Union Territories																				
Andaman & Nicobar Islands (UT)	1,124	5.5	1,125	22.3	1,065	6.3	919	24.8	.	.	.	.	.	.	.	.	.	.	.	.
Chandigarh (UT)	321	4.1	322	19.3	306	5.2	285	16.8	.	.	.	.	.	.	.	.	.	.	.	.
Dadra & Nagar Haveli and daman & Diu (UT)	820	2.4	899	22.6	725	2.8	771	23.0	.	.	.	.	.	.	.	.	.	.	.	.
Jammu & Kashmir (UT)	8,920	4.5	8,471	9.1	7,241	4.1	7,106	13.3	1,023	4.2	962	18.2	1162	6.5	1,028	22.0	1,084	5.4	1,087	20.6
Ladakh (UT)	932	4.1	863	7.3	717	5.2	738	11.3	.	.	.	.	.	.	.	.	.	.	.	.
Lakshadweep (UT)	404	2.7	541	16.5	401	2.5	415	21.0	.	.	.	.	.	.	.	.	.	.	.	.
NCT of Delhi (UT)	4,083	4.1	3,767	18.5	2,637	3.7	2,410	17.0	1,112	3.9	926	19.9	1,015	3.1	934	21.7	1,130	4.1	973	23.3
Puducherry (UT)	1,482	1.6	1,733	24.7	1,367	1.4	1,465	24.1	.	.	.	.	.	.	.	.	.	.	.	.

Table S4: Sample size (n) of ever-married men and women aged less than 45 years and unweighted percentage (%) of widows/widowers among them in 2021, 2016, 2006, 1999, and 1993 across states and union territories of India.

	2021				2016				2006				1999				1993			
	Male		Female		Male		Female		Male		Female		Male		Female		Male		Female	
	n	%	n	%	n	%	n	%	n	%	n	%	n	%	n	%	n	%	n	%
India	352,661	0.8	488,199	2.7	364,566	0.9	489,921	2.7	68,555	0.8	93,544	3.2	67,905	1.1	91,601	3.0	67,407	1.1	91,305	2.9
States																				
Andhra Pradesh	5,511	0.8	8,069	5.3	5,836	0.7	8,347	4.8	4,001	0.3	5,590	4.2	1,723	0.9	2,468	4.5	2,045	0.9	2,760	3.5
Arunachal Pradesh	8,939	0.8	11,950	2.2	7,785	1.1	10,031	2.7	976	1.5	1,189	3.6	9,99	2.6	1,187	4.6	676	1.0	926	4.0
Assam	17,186	0.7	24,317	2.7	14,665	0.8	20,186	3.0	1,896	0.6	2,822	4.5	2,200	0.9	3,227	4.3	2,103	1.2	3,042	5.1
Bihar	18,099	0.8	30,830	1.7	20,923	0.8	33,541	1.9	1,909	1.4	3,194	2.5	4,057	1.4	5,618	2.5	3,955	1.3	5,375	2.3
Chhattisgarh	14,339	0.9	18,071	3.5	13,322	1.1	16,933	3.3	2,218	0.9	2,893	4.2	774	1.2	966	2.8	980	1.4	1,235	3.1
Goa	711	0.1	1,049	3.2	665	0.2	993	3.8	1,601	0.3	2,324	4.4	849	0.4	1,209	3.6	1,874	0.5	2,864	4.1
Gujarat	19,173	0.9	22,956	2.4	14,393	0.9	17,275	2.5	2,536	0.8	2,948	2.4	3,064	1.5	3,788	3.3	3,212	1.1	3,889	2.2
Haryana	12,380	0.9	15,455	3.0	13,031	1.1	15,292	2.4	1,724	0.5	2,282	3.4	2,218	1.4	2,831	2.3	2,514	1.0	3,186	2.0
Himachal Pradesh	5,037	0.7	7,160	2.2	5,086	0.7	7,210	2.6	1,617	0.4	2,219	2.4	1,971	0.9	2,852	2.4	1,898	0.3	3,064	2.5
Jharkhand	12,176	0.6	18,394	2.5	16,069	0.7	21,929	2.8	1,840	1.4	2,533	3.2	1,313	1.7	1,656	2.8	464	1.5	605	2.6
Karnataka	13,989	0.5	20,934	4.2	13,369	0.4	19,509	4.7	3,331	0.6	4,982	4.2	2,893	0.6	4,521	4.2	3,162	0.8	4,655	3.5
Kerala	4,121	0.5	7,137	1.8	4,468	0.4	7,192	2.2	1,476	0.2	2,645	2.0	1,628	0.0	2,893	2.2	2,496	0.4	4,295	2.9
Madhya Pradesh	28,163	1.1	34,938	2.2	37,573	1.1	45,645	2.2	3,995	1.2	5,021	2.7	4,962	1.6	6,300	2.2	4,445	1.6	5,584	1.7
Maharashtra	17,787	0.4	23,937	3.1	16,943	0.4	22,769	3.6	5,379	0.3	7,032	3.5	4,531	0.7	5,735	3.9	3,087	0.7	4,255	3.5
Manipur	3,829	0.8	5,091	3.4	6,673	0.5	8,542	3.2	1,917	0.4	2,661	3.5	985	0.9	1,322	3.3	709	1.0	888	3.6
Meghalaya	6,044	0.6	8,070	2.6	4,197	0.6	5,488	2.8	987	1.4	1,319	4.6	733	1.4	978	2.8	791	0.3	1,013	3.9
Mizoram	3,357	1.0	4,205	3.2	5,904	1.1	7,330	4.0	856	1.3	1,102	3.7	846	1.0	1,057	4.2	673	0.3	917	2.8
Nagaland	3,631	0.7	4,889	2.4	4,634	0.9	6,208	3.3	1,885	0.7	2,430	2.8	600	1.8	750	2.5	689	0.3	963	4.5
Odisha	13,034	0.7	19,261	3.5	16,128	0.6	22,978	3.1	2,374	0.9	3,422	3.2	3,183	1.0	4,295	3.0	3,298	0.8	4,350	2.9
Punjab	11,511	0.8	14,614	2.7	10,507	0.7	12,716	2.4	2,207	0.6	2,736	3.2	2,067	1.0	2,646	2.5	2,361	0.8	3,111	2.3
Rajasthan	22,262	0.7	29,328	2.0	23,865	0.9	30,758	2.0	2,469	1.1	3,171	2.4	5,895	1.4	7,484	1.7	4,432	1.2	5,393	1.8
Sikkim	1,616	0.3	2,069	2.0	2,589	1.0	3,138	2.2	1,172	0.8	1,387	2.3	885	1.1	1,123	1.8	.	.	.	.
Tamil Nadu	10,561	0.5	17,075	4.1	12,934	0.5	19,310	3.4	2,948	0.4	4,426	4.2	3,083	0.8	4,349	4.4	2,650	1.0	3,900	4.3
Telangana	13,750	0.5	19,833	5.2	4,337	0.6	5,997	5.4	.	.	.	.	1,109	0.2	1,510	3.1	1,218	0.6	1,720	2.8
Tripura	3,794	0.6	5,498	2.5	2,551	0.5	3,543	3.1	907	0.2	1,371	3.3	769	0.9	1,136	4.2	706	1.1	1,086	4.6
Uttar Pradesh	42,792	1.2	60,576	2.0	48,151	1.5	66,212	2.1	6,910	1.5	9,688	2.1	6,396	1.9	8,745	2.2	7,254	2.4	9,743	2.0
Uttarakhand	5,583	0.8	8,918	2.9	7,903	0.7	11,604	3.0	1,623	1.0	2,227	2.6	719	1.1	1,092	3.4	1,158	1.0	1,742	3.6
West Bengal	10,009	0.5	15,156	2.5	9,370	0.5	13,479	2.7	3,615	0.4	5,206	3.3	3,133	0.6	4,371	3.6	3,258	0.7	4,495	3.5
Union Territories																				
Andaman & Nicobar Islands (UT)	1,246	0.8	1,600	3.6	1,390	0.9	1,890	2.9	.	.	.	.	.	.	.	.	.	.	.	.
Chandigarh (UT)	456	0.4	540	2.8	475	0.2	524	1.2	.	.	.	.	.	.	.	.	.	.	.	.
Dadra & Nagar Haveli and daman & Diu (UT)	1,639	0.1	1,777	2.9	1,559	0.4	1,567	2.8	.	.	.	.	.	.	.	.	.	.	.	.
Jammu & Kashmir (UT)	10,950	0.6	12,975	1.0	10,242	0.6	12,960	1.4	1,640	1.0	2,176	1.6	2,105	1.0	2,879	2.3	1,928	0.7	2,928	2.7
Ladakh (UT)	1,050	1.0	1,218	1.0	869	0.5	1,060	0.8	.	.	.	.	.	.	.	.	.	.	.	.
Lakshadweep (UT)	434	0.0	752	0.7	481	0.6	707	2.3	.	.	.	.	.	.	.	.	.	.	.	.
NCT of Delhi (UT)	6,169	0.5	7,281	2.4	4,064	0.5	4,456	2.3	2,546	0.8	2,548	2.9	2,215	1.0	2,613	2.7	3,371	1.2	3,321	2.1
Puducherry (UT)	1,333	0.4	2,276	4.2	1,615	0.1	2,602	4.1	.	.	.	.	.	.	.	.	.	.	.	.

Table S5: Prevalence of widowhood (95% CI) among ever-married men and women in 2021 and 1993 across states and union territories of India.

	75 years or above				65 to 74 years				45 to 64 years				Less than 45 years			
	Male		*Female*		*Male*		*Female*		*Male*		*Female*		*Male*		*Female*	
	1993	2021	1993	2021	1993	2021	1993	2021	1993	2021	1993	2021	1993	2021	1993	2021
**India**	32.3 (30.8,33.9)	26.3 (25.8,26.8)	81.5 (80.2,82.8)	75.3 (74.8,75.8)	16.7 (15.9,17.4)	11.6 (11.3,11.8)	61.1 (60.0,62.2)	50.1 (49.7,50.5)	6.7 (6.4,6.9)	4.0 (3.9,4.0)	26.6 (26.1,27.1)	19.1 (18.9,19.2)	1.2 (1.1,1.3)	0.7 (0.7,0.8)	2.8 (2.7,2.9)	2.7 (2.7,2.8)
**States**																
**Andhra Pradesh**	37.1 (25.7,48.5)	20.7 (16.7,24.7)	97 (92.9,101.1)	83.4 (79.8,87.1)	18.8 (13.9,23.7)	10.8 (9.1,12.5)	79.2 (73.7,84.7)	60.3 (57.5,63.1)	6.7 (5.1,8.3)	3.6 (3,4.1)	35.5 (32.6,38.5)	24.7 (23.5,25.8)	0.8 (0.4,1.2)	0.8 (0.6,1)	3.5 (2.8,4.2)	5.3 (4.8,5.8)
**Arunachal Pradesh**	30.0 (13.3,46.6)	30.7 (27.1,34.3)	71.4 (51.6,91.2)	70.9 (66.7,75.1)	21.4 (10.5,32.2)	19.2 (17.2,21.3)	56.2 (38.7,73.7)	45 (42,47.9)	7.3 (4.6,10.1)	4.9 (4.4,5.4)	23.7 (18.5,28.9)	16.3 (15.4,17.1)	1 (0.2,1.7)	0.8 (0.6,1)	3.9 (2.7,5.2)	2 (1.8,2.3)
**Assam**	28.3 (21,35.6)	17.9 (15.6,20.2)	91.7 (85.9,97.4)	77.5 (74.8,80.3)	17.4 (12.6,22.2)	9.9 (8.7,11)	70.5 (64.2,76.7)	60.1 (58,62.2)	6.8 (5.3,8.3)	3.5 (3.2,3.8)	37.8 (34.6,41.1)	22.5 (21.8,23.3)	1.5 (1,2.1)	0.6 (0.5,0.7)	4.9 (4.2,5.7)	2.8 (2.6,3)
**Bihar**	30.2 (23.4,36.9)	34.1 (31.9,36.3)	70.5 (63.7,77.2)	73 (70.8,75.2)	19 (15.5,22.6)	14.3 (13.3,15.3)	50.6 (45.5,55.7)	45.1 (43.4,46.7)	8.8 (7.5,10.2)	5.3 (4.9,5.7)	21.9 (19.9,24)	14.8 (14.2,15.3)	1.3 (0.9,1.6)	0.7 (0.6,0.8)	2.3 (1.9,2.7)	1.6 (1.5,1.8)
**Chhattisgarh**	30.8 (15,46.6)	27.5 (24.5,30.5)	83.5 (70.2,96.8)	76.5 (73.7,79.3)	18 (10.7,25.3)	15.4 (14,16.9)	50 (39.3,60.7)	52.2 (50.1,54.3)	6.3 (3.9,8.6)	4.7 (4.3,5.1)	19.4 (15.5,23.4)	20.3 (19.5,21.1)	1.2 (0.5,1.9)	0.8 (0.7,1)	2.6 (1.7,3.5)	3.4 (3.1,3.6)
**Goa**	25.2 (17.1,33.3)	15.5 (7.9,23.1)	85.6 (79.9,91.3)	83.6 (76.6,90.7)	14.1 (9.9,18.2)	6.5 (3.1,9.8)	77.6 (73.3,81.8)	53.7 (47.4,60)	4.1 (3.1,5.1)	1.7 (0.9,2.6)	32.5 (30.1,34.8)	21.1 (18.6,23.7)	0.4 (0.1,0.7)	0.1 (0,0.4)	4 (3.3,4.8)	3 (2,4.1)
**Gujarat**	43.2 (35,51.4)	29 (26.6,31.4)	81.1 (75.2,87)	71.2 (69.1,73.4)	16.7 (12.9,20.6)	10.6 (9.6,11.6)	62.3 (57.2,67.5)	48 (46.3,49.6)	6.7 (5.5,8)	4.4 (4.1,4.8)	24.2 (21.9,26.4)	17.3 (16.7,17.9)	1.1 (0.7,1.4)	0.7 (0.6,0.9)	2.2 (1.7,2.6)	2.3 (2.1,2.5)
**Haryana**	35.3 (28.4,42.1)	34.2 (31.2,37.3)	76.3 (68.6,84)	73.2 (70.5,75.8)	20.2 (16.1,24.4)	13.2 (11.8,14.6)	42 (36.6,47.4)	43.7 (41.7,45.8)	6.2 (4.6,7.8)	4.5 (4,4.9)	18.2 (15.6,20.7)	19.8 (19,20.6)	0.9 (0.6,1.3)	0.8 (0.6,1)	2 (1.5,2.5)	3 (2.7,3.3)
**Himachal Pradesh**	31.1 (24,38.1)	28.5 (25.1,31.8)	84.2 (78.7,89.7)	76.4 (73.5,79.2)	12.6 (9,16.2)	11.9 (10.1,13.6)	58.1 (51.9,64.2)	45 (42.2,47.7)	7.7 (6.2,9.2)	2.9 (2.4,3.3)	23.2 (20.8,25.7)	17.1 (16.1,18.2)	0.3 (0.1,0.6)	0.8 (0.5,1)	2.6 (2,3.1)	2 (1.7,2.3)
**Jharkhand**	45.3 (27.2,63.4)	29.3 (26.3,32.3)	54.5 (26.3,82.7)	70.9 (67.7,74.1)	13.2 (2.6,23.9)	14.8 (13.3,16.2)	56.7 (40.2,73.1)	51.6 (49.4,53.8)	9.8 (5.7,13.8)	4.9 (4.5,5.4)	29.7 (23.4,36.1)	19.6 (18.8,20.4)	1.2 (0.2,2.3)	0.6 (0.4,0.7)	2.5 (1.2,3.7)	2.4 (2.1,2.6)
**Karnataka**	31.9 (24.7,39)	16.4 (14.4,18.3)	88.6 (84.1,93)	79.4 (77.4,81.4)	14 (10.8,17.3)	7.3 (6.5,8.2)	72.3 (67.9,76.8)	52.6 (50.9,54.2)	4.5 (3.5,5.5)	1.9 (1.7,2.2)	35.5 (33.2,37.8)	23.3 (22.6,24.1)	0.7 (0.4,1)	0.4 (0.3,0.6)	3.5 (2.9,4)	3.9 (3.7,4.2)
**Kerala**	30.2 (23.6,36.7)	17.7 (15,20.4)	86 (81.6,90.5)	81.5 (79.3,83.8)	9.1 (6.4,11.8)	5.1 (4,6.1)	65 (60.8,69.3)	53 (50.8,55.2)	2.2 (1.5,3)	1.4 (1.1,1.7)	26.4 (24.3,28.5)	18 (17.1,18.9)	0.4 (0.1,0.6)	0.4 (0.2,0.6)	2.9 (2.4,3.4)	1.8 (1.4,2.1)
**Madhya Pradesh**	24.5 (18.3,30.6)	30.5 (28.5,32.6)	82 (76.6,87.4)	72.4 (70.5,74.3)	18.6 (15.2,22)	13.2 (12.2,14.1)	54.3 (49.4,59.2)	46.1 (44.6,47.6)	8.3 (7,9.6)	4.9 (4.6,5.2)	23.3 (21.2,25.3)	15.6 (15.1,16.1)	1.4 (1,1.7)	1.2 (1,1.3)	1.6 (1.3,2)	2.4 (2.2,2.5)
**Maharashtra**	28.1 (20.9,35.2)	19.4 (17.6,21.2)	87.6 (82,93.3)	71.7 (69.7,73.7)	13.9 (10.7,17.2)	7.6 (6.8,8.4)	67.9 (63.3,72.5)	50.1 (48.7,51.5)	4.5 (3.5,5.6)	2.3 (2,2.5)	28 (25.7,30.2)	19.6 (19,20.3)	0.6 (0.3,0.9)	0.3 (0.2,0.4)	3.4 (2.9,4)	3 (2.7,3.2)
**Manipur**	17.6 (4.6,30.6)	30.4 (26.2,34.6)	74 (62.2,85.8)	75.3 (71.1,79.4)	13.4 (6.8,20)	8.3 (6.5,10.2)	50.6 (39.2,62)	42.6 (39.3,45.9)	4.8 (2.7,6.9)	2.6 (2.1,3.2)	22.7 (18.6,26.8)	18.7 (17.3,20)	0.9 (0.2,1.7)	0.6 (0.4,0.9)	3.6 (2.3,4.8)	2.9 (2.5,3.4)
**Meghalaya**	31.2 (7.7,54.7)	33.6 (26.2,41.1)	84.2 (67.3,101)	75.1 (68.9,81.2)	10.6 (1.7,19.5)	11.4 (8.3,14.4)	50 (32.9,67)	54.6 (49.7,59.4)	4.7 (2.4,7.1)	3.7 (3.1,4.4)	25 (19.7,30.2)	23.5 (22,25)	0.2 (0,0.6)	0.7 (0.5,0.9)	3.8 (2.6,5)	2.6 (2.2,2.9)
**Mizoram**	23 (6.5,39.5)	24.4 (19.7,29.1)	70.5 (55,86.1)	66 (60.5,71.4)	12.8 (4.9,20.7)	9.4 (7.2,11.7)	36.7 (25.2,48.3)	39.3 (35.3,43.3)	2.1 (0.6,3.6)	4.5 (3.8,5.3)	16.3 (12.3,20.2)	20.3 (18.8,21.8)	0.2 (0,0.7)	1 (0.6,1.3)	2.8 (1.7,3.9)	3.4 (2.9,4)
**Nagaland**	15 (0,31)	22.4 (19.2,25.7)	14.2 (0,42.2)	44.6 (39.7,49.5)	3.1 (0,9.2)	10.9 (8.8,13)	16.6 (0,34.3)	31.2 (27.6,34.7)	1.8 (0.4,3.1)	4 (3.4,4.6)	9.7 (5.9,13.4)	13.5 (12.5,14.6)	0.2 (0,0.6)	0.6 (0.3,0.8)	4.4 (3.1,5.7)	2.4 (2,2.9)
**Odisha**	37.5 (30.4,44.7)	28.5 (26.2,30.7)	85.1 (79,91.2)	74.8 (72.5,77.1)	15.1 (11.9,18.2)	12.5 (11.3,13.7)	64.6 (59.8,69.4)	50 (48.1,51.8)	6.4 (5.2,7.6)	3.7 (3.3,4)	26.7 (24.6,28.9)	19.9 (19.1,20.6)	0.8 (0.5,1.1)	0.5 (0.4,0.6)	2.8 (2.3,3.3)	3.4 (3.1,3.6)
**Punjab**	37.3 (30.6,44.1)	34.6 (32,37.3)	71.2 (64.2,78.2)	68.8 (66.2,71.5)	16.4 (13,19.9)	14.4 (13.1,15.6)	44 (38.5,49.5)	40.4 (38.5,42.3)	6.1 (4.7,7.4)	5.1 (4.6,5.6)	18.9 (16.7,21.2)	17.4 (16.7,18.2)	0.7 (0.4,1.1)	0.7 (0.5,0.8)	2.3 (1.7,2.8)	2.7 (2.4,2.9)
**Rajasthan**	33.8 (27,40.6)	26 (23.8,28.3)	77.2 (71,83.4)	72.5 (70.4,74.5)	15 (12,18)	12 (11,13)	52 (47,56.9)	42.5 (40.9,44.1)	7.5 (6.4,8.6)	4.2 (3.8,4.5)	20.8 (19.1,22.6)	14.9 (14.3,15.4)	1.1 (0.8,1.4)	0.7 (0.6,0.8)	1.7 (1.4,2.1)	1.9 (1.8,2.1)
**Sikkim**	.	23.7 (17.2,30.3)	.	72.7 (64.7,80.8)	.	10.9 (7.2,14.5)	.	34.1 (28,40.2)	.	4.9 (3.8,6.1)	.	15.1 (13.1,17.2)	.	0 (0,0.1)	.	1.7 (1.2,2.3)
**Tamil Nadu**	23.2 (16.6,29.8)	21.6 (19.4,23.8)	86.2 (80.4,92)	80.6 (78.6,82.6)	16.9 (13.2,20.5)	9.3 (8.3,10.3)	67.3 (62.4,72.1)	56.8 (55.1,58.5)	6 (4.8,7.2)	2.8 (2.5,3.1)	33.5 (31.2,35.8)	23.1 (22.4,23.9)	1 (0.6,1.4)	0.5 (0.4,0.7)	4.3 (3.6,4.9)	3.9 (3.6,4.2)
**Telangana**	36.3 (23.5,49.1)	22.5 (20,25)	92.1 (83.4,100.7)	81.1 (78.9,83.3)	15.9 (10.4,21.5)	9.1 (8.1,10.2)	64.8 (56.6,73)	55.6 (53.8,57.4)	4.2 (2.6,5.8)	2.8 (2.4,3.1)	26.2 (22.6,29.7)	25.4 (24.6,26.2)	0.5 (0.1,0.9)	0.5 (0.4,0.6)	2.7 (2,3.5)	5.2 (4.9,5.5)
**Tripura**	23 (12.7,33.4)	18 (13.7,22.3)	89.4 (82.8,95.9)	80 (75.7,84.2)	13.9 (7.7,20.1)	8 (5.9,10.2)	73.3 (64.1,82.5)	58.7 (54.5,62.8)	3.7 (1.8,5.6)	2.7 (2.1,3.3)	30.4 (25.9,34.9)	21.4 (19.8,22.9)	1.1 (0.3,1.9)	0.6 (0.4,0.9)	4.6 (3.3,5.8)	2.3 (1.9,2.7)
**Uttar Pradesh**	35.2 (30.5,39.8)	34.9 (33.4,36.5)	72.6 (67.6,77.5)	70.4 (68.9,71.9)	20.6 (18,23.1)	16.7 (15.9,17.5)	53.1 (49.6,56.6)	44.5 (43.3,45.6)	8.9 (7.9,9.8)	6.2 (5.9,6.5)	20.8 (19.4,22.2)	15.8 (15.4,16.2)	2.4 (2,2.7)	1.1 (1,1.2)	1.9 (1.6,2.1)	1.9 (1.8,2)
**Uttarakhand**	16.9 (8.4,25.4)	28.4 (25,31.8)	74.2 (64.2,84.2)	76.1 (73.1,79)	18.4 (12.7,24.1)	11 (9.4,12.6)	56.9 (49.1,64.7)	51.1 (48.6,53.6)	7.8 (5.7,9.9)	4 (3.5,4.6)	28.5 (24.9,32.2)	20 (19,21.1)	0.4 (0,0.7)	0.8 (0.5,1)	3.5 (2.7,4.4)	2.8 (2.5,3.2)
**West Bengal**	32.6 (25.3,39.9)	19.6 (16.7,22.5)	92.6 (88.5,96.7)	85.2 (82.7,87.8)	11.2 (8,14.4)	8.9 (7.6,10.2)	77.9 (73.3,82.6)	61.9 (59.5,64.3)	5 (3.9,6.1)	2.6 (2.3,3)	35.3 (32.9,37.8)	22 (21,22.9)	0.6 (0.3,0.9)	0.4 (0.3,0.6)	3.4 (2.9,4)	2.2 (2,2.5)
**Union Territories**																
**Jammu & Kashmir**	27.6 (20.7,34.5)	27.5 (24.3,30.7)	81.7 (75.3,88)	60.4 (56.5,64.4)	9 (5.9,12.1)	16.5 (14.9,18.1)	57.5 (51.4,63.5)	30.7 (28.4,33)	5.9 (4.5,7.3)	4.7 (4.2,5.1)	20.3 (17.9,22.7)	9.4 (8.8,10.1)	0.7 (0.3,1.1)	0.6 (0.4,0.7)	2.6 (2,3.1)	1.1 (0.9,1.2)
**NCT of Delhi**	35.2 (25,45.5)	30.5 (25.4,35.6)	81.7 (73.2,90.1)	68.2 (63.1,73.2)	18.3 (13.2,23.4)	13.3 (10.9,15.7)	56 (48.9,63.1)	46.8 (43.3,50.3)	4 (2.9,5.2)	3.9 (3.3,4.5)	23.3 (20.6,25.9)	18.6 (17.3,19.8)	1.1 (0.8,1.5)	0.4 (0.2,0.6)	2.1 (1.6,2.6)	2.4 (2,2.7)
**Andaman & Nicobar Islands**	.	28.9 (20.1,37.8)	.	84.2 (76.6,91.9)	.	15.2 (10.7,19.8)	.	61.2 (54.5,67.9)	.	5.3 (4,6.6)	.	23.7 (21.2,26.1)	.	0.6 (0.2,1)	.	3.8 (2.8,4.7)
**Chandigarh**	.	34.2 (21.6,46.9)	.	58.7 (42.8,74.5)	.	7.5 (1.5,13.5)	.	34.5 (24.2,44.7)	.	4.4 (2.1,6.6)	.	19.6 (15.2,23.9)	.	0.3 (0,0.8)	.	2.7 (1.3,4.1)
**Dadra & Nagar Haveli and daman & Diu**	.	25.8 (15.4,36.1)	.	71.7 (63.4,80)	.	8.5 (4.5,12.6)	.	58.8 (53,64.6)	.	2.3 (1.3,3.3)	.	20.6 (18,23.3)	.	0.2 (0,0.4)	.	2.7 (2,3.5)
**Ladakh**	.	35.5 (24.2,46.8)	.	45 (28.6,61.5)	.	14.9 (9.9,19.9)	.	14 (8.1,19.9)	.	4.2 (2.9,5.5)	.	7 (5.3,8.7)	.	0.8 (0.3,1.4)	.	1 (0.4,1.6)
**Lakshadweep**	.	15.5 (4.4,26.7)	.	88.1 (79.2,97)	.	13.2 (7.6,18.9)	.	48.1 (39,57.2)	.	2.7 (1.1,4.2)	.	16.3 (13.2,19.4)	.	0 (0,0)	.	0.6 (0,1.1)
**Puducherry**	.	28.2 (21.3,35.1)	.	89.6 (85.7,93.6)	.	10.1 (7.1,13.1)	.	58 (53.3,62.6)	.	1.5 (0.9,2.1)	.	27.2 (25.1,29.3)	.	0.4 (0,0.8)	.	5.3 (4.4,6.2)

Table S6: Headcount (N) of widowhood among ever-married men and women in 1993, and 2021, across states and union territories of India.

	**75 years or above**				**65 to 74 years**				**45 to 64 years**				**Less than 45 years**			
	Male		Female		Male		Female		Male		Female		Male		Female	
	1993	2021	1993	2021	1993	2021	1993	2021	1993	2021	1993	2021	1993	2021	1993	2021
**India**	17,54,533	39,24,912	41,51,674	1,29,97,465	20,05,253	33,97,347	69,52,607	1,57,81,424	39,20,369	50,27,236	1,40,25,051	2,37,18,301	25,84,177	27,67,866	54,26,085	94,65,338
**States**																
**Andhra Pradesh**	64,162	1,02,359	1,67,173	4,73,110	99,614	1,44,372	4,11,180	8,01,934	1,87,103	2,04,238	9,73,699	14,45,733	90,004	1,29,641	3,33,726	7,90,120
**Arunachal Pradesh**	1,285	2,603	2,231	4,908	1,503	3,125	2,548	6,580	4,227	5,230	9,464	14,109	2,024	2,603	7,363	5,435
**Assam**	44,291	51,743	90,293	2,05,022	41,335	52,712	1,52,023	3,14,332	93,199	1,14,505	3,90,765	6,33,924	72,568	64,862	2,29,546	2,66,405
**Bihar**	1,41,437	4,23,910	3,44,384	8,97,615	2,25,263	3,95,337	5,06,086	10,63,637	4,63,419	4,75,974	9,96,297	14,06,804	2,85,586	2,07,094	4,83,640	5,19,947
**Chhattisgarh**	33,485	76,116	73,547	2,43,074	50,541	95,575	1,23,138	3,49,760	80,602	1,44,645	2,14,868	5,86,796	63,572	79,484	1,15,319	2,64,265
**Goa**	1,351	3,424	6,286	24,022	1,609	2,708	13,783	30,386	3,659	3,837	26,016	47,257	880	575	7,871	12,192
**Gujarat**	1,01,111	1,92,630	2,38,394	6,40,652	88,727	1,44,090	3,51,804	7,73,070	1,94,401	2,95,356	6,05,371	10,70,307	1,20,908	1,64,127	1,98,738	3,75,654
**Haryana**	65,854	95,762	89,652	2,58,196	62,849	72,871	1,28,148	2,78,263	62,673	1,10,697	1,66,662	4,65,039	48,127	71,539	86,300	2,08,151
**Himachal Pradesh**	15,636	36,295	46,948	1,34,165	10,554	21,179	39,151	93,388	27,702	24,018	79,134	1,37,833	3,656	15,097	29,683	38,050
**Jharkhand**	51,151	88,920	26,593	2,08,157	16,819	95,777	71,684	3,23,346	85,904	1,51,398	2,43,232	5,88,301	42,806	54,916	76,941	2,17,942
**Karnataka**	85,184	1,31,927	2,91,944	8,26,805	87,689	1,25,542	4,59,791	10,11,338	1,43,609	1,35,919	10,43,439	15,96,239	79,662	92,882	3,74,553	7,30,352
**Kerala**	59,235	1,11,049	2,10,747	8,18,145	35,848	56,449	3,17,038	7,68,613	45,976	65,485	5,06,206	8,57,883	20,694	34,388	1,79,405	1,48,530
**Madhya Pradesh**	81,928	2,51,245	2,86,698	7,03,141	1,32,303	2,13,890	3,71,733	7,56,312	2,97,975	3,90,731	7,11,609	11,56,751	2,11,999	3,03,837	2,15,188	4,97,189
**Maharashtra**	1,30,701	3,40,995	3,61,132	14,25,705	1,62,705	2,60,396	8,16,961	20,63,878	2,52,061	3,00,074	14,23,980	24,14,692	1,23,177	1,46,872	6,22,937	9,99,529
**Manipur**	1,596	10,164	11,087	25,175	3,267	4,313	10,023	25,332	5,986	5,899	26,596	41,363	3,772	4,243	11,865	16,411
**Meghalaya**	1,413	3,976	4,711	13,244	1,240	2,584	4,763	15,573	5,020	8,176	20,267	50,104	1,145	6,328	15,361	20,572
**Mizoram**	606	3,074	2,526	8,968	798	1,814	2,504	8,777	957	4,817	6,038	20,606	409	2,771	3,661	8,021
**Nagaland**	555	5,246	193	7,327	162	2,753	550	7,128	1,534	6,077	4,624	17,937	750	1,898	11,088	6,569
**Odisha**	75,330	1,96,335	1,26,370	5,02,991	74,341	1,31,929	2,80,374	5,62,588	1,40,858	1,69,623	5,35,457	8,83,299	59,290	62,860	1,86,360	4,03,568
**Punjab**	72,594	1,43,096	1,16,553	2,95,936	62,842	1,11,941	1,34,267	3,37,204	86,000	1,48,368	2,40,986	5,02,543	35,779	60,637	98,473	1,94,066
**Rajasthan**	88,187	1,98,105	1,95,306	7,42,950	99,151	1,89,704	2,80,156	7,11,737	2,59,550	2,92,587	6,47,031	9,91,587	1,45,183	1,77,386	1,86,343	4,12,031
**Sikkim**	.	1,817	.	4,205	.	1,028	.	3,248	.	3,012	.	7,583	.	117	.	2,572
**Tamil Nadu**	74,345	2,39,585	2,49,198	11,03,025	1,19,899	1,96,753	4,84,255	13,95,760	2,18,994	2,50,338	11,99,045	22,07,695	1,09,926	1,09,761	4,73,424	8,01,939
**Telangana**	49,355	83,744	90,016	3,60,827	58,469	75,400	2,08,038	4,98,554	73,087	91,498	4,13,084	8,80,197	35,002	52,648	1,65,143	4,98,390
**Tripura**	5,745	8,306	30,334	45,946	5,713	6,395	25,068	45,499	6,805	11,360	51,618	80,655	6,208	8,338	26,696	27,813
**Uttar Pradesh**	3,46,978	8,00,546	5,97,151	17,07,287	4,28,997	6,99,531	9,99,425	18,74,391	8,60,388	11,47,960	17,87,346	28,18,070	8,64,683	6,84,813	6,28,117	10,51,537
**Uttarakhand**	10,929	37,422	45,289	1,18,820	20,576	25,310	65,293	1,41,146	44,124	40,757	1,41,071	1,99,939	6,128	23,407	59,092	79,517
**West Bengal**	1,28,404	1,99,967	3,94,552	9,92,487	92,369	1,78,582	6,20,664	12,62,642	2,37,490	2,83,360	14,13,330	21,24,194	1,11,185	1,51,071	5,37,329	6,90,378
**Union Territories**																
**Andaman & Nicobar Islands (UT)**	.	1,115	.	3,501	.	1,023	.	4,355	.	2,253	.	9,200	.	724	.	3645
**Chandigarh (UT)**	.	5,975	.	7,531	.	1,469	.	8,285	.	4,431	.	18,500	.	1,129	.	7,116
**Dadra & Nagar Haveli and daman & Diu (UT)**	.	628	.	2,502	.	481	.	5,195	.	834	.	6,575	.	450	.	3,691
**Jammu & Kashmir (UT)**	9,285	28,739	23,500	56,347	5,197	40,882	28,697	60,315	14,539	58,249	45,817	1,02,378	5,549	20,263	21,002	28,410
**Ladakh (UT)**	.	641	.	448	.	597	.	454	.	959	.	1,347	.	509	.	458
**Lakshadweep (UT)**	.	115	.	931	.	260	.	909	.	186	.	1,391	.	0	.	117
**NCT of Delhi (UT)**	12,402	43,162	28,866	1,13,189	14,873	37,876	43,460	1,56,783	22,528	72,318	1,02,001	2,91,999	33,505	29,252	40,920	1,18,386
**Puducherry (UT)**	.	4,178	.	21,113	.	2,700	.	20,714	.	2,066	.	39,475	.	1,347	.	16,368

Note: The age group of less than 45 includes those aged between 13 and 44

Table S7: Headcount (N) of widows/widowers among ever-married men and women by age groups in 2016, 2006, and 1999 across states and union territories of India.

	75 years or above						65 to 74 years						45 to 64 years						Less than 45 years					
	Male			Female			Male			Female			Male			Female			Male			Female		
	2016	2006	1999	2016	2006	1999	2016	2006	1999	2016	2006	1999	2016	2006	1999	2016	2006	1999	2016	2006	1999	2016	2006	1999
**States**																								
Andhra Pradesh	115,847	142,841	110,399	522,359	511,295	308,797	153,993	216,203	157,060	925,238	1,084,660	581,313	190,466	300,477	240,374	1,335,058	1,877,076	1,377,813	113,960	89,553	122,998	744,312	952,587	624,101
Arunachal Pradesh	1,795	2,217	1,592	3,253	3,276	3,151	2,097	2,912	4,073	4,677	5,334	5,776	5,335	6,619	10,289	14,567	15,428	11,988	3,269	5,276	7,424	6,717	9,470	11,014
Assam	55,776	65,769	17,371	193,263	181,305	99,834	57,660	53,545	24,442	301,155	284,827	143,203	103,630	106,485	66,057	607,649	527,688	409,523	70,972	47,184	60,375	240,465	320,015	227,333
Bihar	335,427	185,228	160,796	666,254	523,735	264,770	352,952	230,892	218,410	946,394	878,288	499,704	434,114	428,468	415,722	1,248,718	1,145,079	892,130	205,474	33,1795	273,141	514,259	636,369	472,176
Chhattisgarh	62,578	39,317	32,367	198,748	89,910	144,010	96,927	77,176	63,350	333,574	290,437	265,717	129,323	111,340	123,096	466,570	420,529	316,439	89,224	64,679	69,011	231,627	266,910	138,648
Goa	3,853	2,306	2,109	17,836	12,424	14,791	2,885	3,672	1,686	28,115	22,577	23,297	3,544	3,039	6,096	35,551	39,980	40,174	538	903	1,003	12,182	15,459	10,247
Gujarat	149,933	83,491	124,820	494,564	344,854	328,483	153,149	154,206	135,919	671,625	547,865	522,104	259,690	211,969	245,361	925,976	838,531	823,456	186,054	142,162	213,075	373,390	338,960	397,434
Haryana	80,606	66,728	51,481	147,343	127,494	110,468	74,969	65,857	88,855	217,732	178,187	175,606	129,386	60,413	62,209	378,739	293,785	208,692	106,523	32,433	72,505	174,399	180,870	108,831
Himachal Pradesh	31,377	23,566	18,321	105,262	54,478	54,115	16,083	17,634	19,471	91,808	76,929	69,079	18,584	21,692	26,241	130,668	112,521	112,898	10,986	7,846	13,930	41,672	36,810	33,013
Jharkhand	93,163	45,929	39,630	230,349	144,267	49,925	96,558	59,770	51,676	349,557	225,880	121,809	150,640	120,219	115,094	543,708	527,804	348,017	62,016	132,080	114,628	236,263	253,840	166,388
Karnataka	112,953	117,398	92,136	607,276	448,475	434,306	134,335	100,080	80,502	853,603	858,494	779,630	152,376	128,999	171,970	1,429,796	1,499,073	1,180,756	60,302	101,294	71,176	732,228	708,295	579,270
Kerala	94,546	84,067	78,063	573,328	418,301	398,126	70,102	35,066	55,092	619,236	575,105	480,992	78,741	37,254	58,558	800,762	656,917	717,309	21,742	13,033	0	163,571	149,244	184,733
Madhya Pradesh	215,306	160,312	137,595	620,783	359,028	347,607	209,232	180,952	168,113	773,493	660,552	571,310	345,958	312,046	274,250	1,017,849	817,999	747,424	282,847	319,912	273,424	465,470	487,929	323,251
Maharashtra	329,248	197,907	197,241	1,178,205	757,037	632,255	225,856	216,618	217,613	1,801,499	1,306,591	1,308,846	247,619	278,121	238,112	2,103,179	1,852,797	1,945,658	156,157	70,469	212,863	1,229,828	935,187	1,007,823
Manipur	7,415	6,385	3,942	21,352	13,862	16,979	4,061	4,460	5,632	22,077	17,219	21,355	6,274	5,362	4,819	32,361	29,500	29,652	2,811	2,253	4,256	16,494	15,015	13,962
Meghalaya	3,461	2,699	3,958	12,809	14,232	8,607	4,014	5,568	2,307	13,858	18,576	13,926	8,474	8,603	5,133	45,732	48,529	36,956	4,158	10,381	6,265	17,745	23,560	11,630
Mizoram	2,386	1,764	2,003	8,076	6,196	4,606	1,907	1,654	1,567	7,523	5,659	7,240	3,620	2,219	2,923	13,466	8,490	10,970	2,851	2,905	2,002	11,039	7,205	6,804
Nagaland	4,563	3,319	2,799	9,697	9,788	9,910	2,715	1,755	2,829	8,766	6,520	9,458	3,317	5,419	4,682	17,574	16,918	20,874	2,866	2,442	6,726	10,023	7,736	7,916
Odisha	144,670	116,846	89,928	421,500	245,641	262,428	125,940	97,099	122,797	503,183	533,749	426,917	171,975	183,292	187,418	674,280	731,487	627,092	61,714	103,610	95,056	338,893	338,869	255,373
Punjab	128,362	95,131	70,512	232,977	178,725	158,920	126,041	80,630	106,895	294,902	268,521	256,976	148,527	109,809	94,915	414,967	293,262	301,662	65,013	50,595	61,971	161,594	205,926	129,637
Rajasthan	188,652	11,1670	103,810	604,856	325,679	289,594	152,463	132,634	135,591	649,983	501,306	491,929	255,848	282,498	256,281	845,819	931,989	746,651	173,288	199,078	219,233	351,380	381,887	234,420
Sikkim	2,502	1,764	1,011	3,303	2,574	1,692	1,841	2,207	1,485	3,321	5,529	3,443	3,576	3,822	2,462	5,508	8,745	4,572	1,604	1,561	1,373	2,861	3,298	1,884
Tamil Nadu	192,172	167,481	152,827	606,794	527,088	455,454	150,338	172,942	195,353	1,005,958	949,589	994,058	236,691	195,460	271,783	1,752,058	1,710,478	1,749,218	95,848	58,211	121,662	757,412	628,183	774,165
Telangana	79,416	.	23,244	322,727	.	159,385	106,067	.	62,438	590,289	.	286,615	108,731	.	99,329	872,042	.	736,138	64,202	.	17,243	574,449	.	260,447
Tripura	6,442	6,792	7,995	40,988	25,894	29,573	7,448	6,898	5,993	43,190	55,136	37,421	5,747	5,597	9,543	74,528	75,202	79,663	5,090	2,384	7,220	31,813	32,159	35,680
Uttar Pradesh	687,611	478,709	498,350	1,394,377	925,983	818,447	677,884	747,426	541,860	1,843,280	1,708,627	1,217,012	971,388	991,912	971,160	2,343,059	2,296,210	2,016,317	700,492	775,023	748,879	949,695	925,965	826,399
Uttarakhand	27,659	15,881	14,248	87,291	61,341	58,920	24,998	23,994	15,805	114,742	94,917	84,607	37,063	29,732	38,273	194,358	178,360	167,684	15,403	21,365	24,822	72,612	53,594	71,266
West Bengal	195,431	115,234	129,673	971,565	643,624	482,579	177,209	140,714	181,895	1,282,279	1,045,529	971,045	257,567	208,221	198,538	1,879,749	1,830,991	1,819,938	142,229	86,192	120,058	689,507	783,151	734,338
**Union Territories**																								
Andaman & Nicobar Islands (UT)	816	.	.	1,952	.	.	1,019	.	.	3,354	.	.	1,795	.	.	7,049	.	.	310	.	.	3,016	.	.
Chandigarh (UT)	3,888	.	.	8,497	.	.	2,345	.	.	10,254	.	.	4,728	.	.	13,426	.	.	618	.	.	2,915	.	.
Dadra & Nagar Haveli and daman & Diu (UT)	656	.	.	1,896	.	.	577	.	.	3,771	.	.	1,147	.	.	6,294	.	.	509	.	.	2,522	.	.
Jammu & Kashmir (UT)	43,181	24,444	26,279	84,880	65,480	53,105	31,916	32,791	30,146	95,159	73,896	65,400	42,947	33,704	41,381	131,149	124,619	118,157	18,034	26,882	22,223	38,228	34,979	44,905
Ladakh (UT)	1,229	.	.	1,142	.	.	761	.	.	1,367	.	.	909	.	.	2,020	.	.	194	.	.	385	.	.
Lakshadweep (UT)	105	.	.	541	.	.	74	.	.	1,257	.	.	220	.	.	1,417	.	.	169	.	.	414	.	.
NCT of Delhi (UT)	32,700	30,627	28,681	82,174	67,581	64,796	63,639	22,738	24,432	123,618	97,061	94,403	45,740	41,050	28,590	224,885	153,510	181,824	20,743	27,985	37,828	85,994	80,446	82,074
Puducherry (UT)	1,611	.	.	9149	.	.	2,146	.	.	17,377	.	.	2,357	.	.	28,633	.	.	154	.	.	17,075	.	.

Table S8: Standardised Absolute Change (SAC) in widowhood prevalence for men and women for ages 75 or above, 65 to 74 years, 45 to 64 years, and less than 45 years between 1993 and 2021, for all states and union territories of India.

	Male Standardized Absolute Change (SAC) (1993-2021)				Female Standardized Absolute Change (SAC) (1993-2021)			
	75 years or above	65 to 74 years	45 to 64 years	less than 45 years	75 years or above	65 to 74 years	45 to 64 years	less than 45 years
India	-0.2	-0.2	-0.1	0.0	-0.2	-0.4	-0.3	0.0
**States**								
Andhra Pradesh	-0.6	-0.3	-0.1	0.0	-0.5	-0.7	-0.4	-0.1
Arunachal Pradesh	0.0	-0.1	-0.1	0.0	0.0	-0.4	-0.3	0.1
Assam	-0.4	-0.3	-0.1	0.0	-0.5	-0.4	-0.5	0.1
Bihar	0.1	-0.2	-0.1	0.0	0.1	-0.2	-0.3	0.0
Chhattisgarh	-0.1	-0.1	-0.1	0.0	-0.3	0.1	0.0	0.0
Goa	-0.3	-0.3	-0.1	0.0	-0.1	-0.9	-0.4	0.0
Gujarat	-0.5	-0.2	-0.1	0.0	-0.4	-0.5	-0.2	0.0
Haryana	0.0	-0.3	-0.1	0.0	-0.1	0.1	0.1	0.0
Himachal Pradesh	-0.1	0.0	-0.2	0.0	-0.3	-0.5	-0.2	0.0
Jharkhand	-0.6	0.1	-0.2	0.0	0.6	-0.2	-0.4	0.0
Karnataka	-0.6	-0.2	-0.1	0.0	-0.3	-0.7	-0.4	0.0
Kerala	-0.4	-0.1	0.0	0.0	-0.2	-0.4	-0.3	0.0
Madhya Pradesh	0.2	-0.2	-0.1	0.0	-0.3	-0.3	-0.3	0.0
Maharashtra	-0.3	-0.2	-0.1	0.0	-0.6	-0.6	-0.3	0.0
Manipur	0.5	-0.2	-0.1	0.0	0.0	-0.3	-0.1	0.0
Meghalaya	0.1	0.0	0.0	0.0	-0.3	0.2	-0.1	0.0
Mizoram	0.0	-0.1	0.1	0.0	-0.2	0.1	0.1	0.0
Nagaland	0.3	0.3	0.1	0.0	1.1	0.5	0.1	0.1
Odisha	-0.3	-0.1	-0.1	0.0	-0.4	-0.5	-0.2	0.0
Punjab	-0.1	-0.1	0.0	0.0	-0.1	-0.1	-0.1	0.0
Rajasthan	-0.3	-0.1	-0.1	0.0	-0.2	-0.3	-0.2	0.0
Sikkim	.	.	.	.	.	.	.	.
Tamil Nadu	-0.1	-0.3	-0.1	0.0	-0.2	-0.4	-0.4	0.0
Telangana	-0.5	-0.2	-0.1	0.0	-0.4	-0.3	0.0	-0.1
Tripura	-0.2	-0.2	0.0	0.0	-0.3	-0.5	-0.3	0.1
Uttar Pradesh	0.0	-0.1	-0.1	0.0	-0.1	-0.3	-0.2	0.0
Uttarakhand	0.4	-0.3	-0.1	0.0	0.1	-0.2	-0.3	0.0
West Bengal	-0.5	-0.1	-0.1	0.0	-0.3	-0.6	-0.5	0.0
**Union Territories**								
Andaman & Nicobar Islands (UT)	.	.	.	.	.	.	.	.
Chandigarh (UT)	.	.	.	.	.	.	.	.
Dadra & Nagar Haveli and daman & Diu (UT)	.	.	.	.	.	.	.	.
Jammu & Kashmir (UT)	0.0	0.3	0.0	0.0	-0.8	-1.0	-0.4	0.1
Ladakh (UT)	.	.	.	.	.	.	.	.
Lakshadweep (UT)	.	.	.	.	.	.	.	.
NCT of Delhi (UT)	-0.2	-0.2	0.0	0.0	-0.5	-0.3	-0.2	0.0
Puducherry (UT)	.	.	.	.	.	.	.	.

Table S9: Comparison of widowhood prevalence and headcount for 1991, 2001, and 2011 census rounds with 1992-93, 1998-99, and 2015-16 rounds of NFHS.

	Census	NFHS	Census Headcount	NFHS Headcount
**1991**	8.3	9.6	34,549,336	40,819,749
**2001**	8.5	9.8	43,869,913	54,370,120
**2011**	8.7	9.4	55,286,392	69,032,126

1991 corresponds to 1992-1993 in NFHS
2001 corresponds to 1998-99 in NFHS
2011 corresponds to 2015-16 in NFHS

Table S10: Prevalence of widowhood (95% CI), widowhood* and their Headcount (N) among ever-married men and women in 2021, 2016, 2006, 1999, and 1993, India.

**Prevalence**		Widowhood (2021)	widowhood* (2021)	Widowhood (2016)	widowhood* (2016)	Widowhood (2006)	widowhood* (2006)	Widowhood (1999)	Widowhood (1993)
45 to 64 years	Male	4.0 (3.9,4.0)	4.0 (3.9,4.0)	4.1 (4.0,4.1)	4.1 (4.0,4.2)	5.0 (4.8,5.2)	4.9 (4.7,5.2)	5.9 (5.7,6.2)	6.7 (6.4,6.9)
	Female	19.1 (18.9,19.2)	19.2 (19.0,19.3)	19.3 (19.2,19.5)	19.4 (19.2,19.5)	24.3 (23.8,24.7)	24.3 (23.8,24.7)	26.4 (26.0,26.9)	26.6 (26.1,27.1)
Less than 45 years	Male	0.7 (0.7,0.8)	0.7 (0.7,0.8)	0.8 (0.8,0.8)	0.8 (0.7,0.8)	0.9 (0.8,1.0)	0.9 (0.8,1.0)	1.1 (1.0,1.2)	1.2 (1.1,1.3)
	Female	2.7 (2.7,2.8)	2.8 (2.8,2.8)	2.8 (2.8,2.9)	2.8 (2.8,2.9)	3.1 (3.0,3.2)	3.1 (3.0,3.2)	3.1 (3.0,3.2)	2.8 (2.7,2.9)
**Headcount**									
45 to 64 years	Male	5,027,236	5,025,762	4,568,047	4,567,427	4,233,838	4,208,073	4,270,659	3,920,369
	Female	23,718,301	23,860,304	20,619,159	20,663,171	19,063,496	19,049,166	17,779,644	14,025,051
Less than 45 years	Male	2,767,866	2,734,950	2,748,362	2,734,704	2,729,483	2,731,386	3,002,369	2,584,177
	Female	9,465,338	9,689,571	9,302,447	9,391,927	8,813,919	8,782,258	7,775,165	5,426,085

widowhood* represents the sensitivity analysis. Widowhood* is computed by considering the self-reported marital status among men and women of reproductive ages.

Table S11: Sample size and weighted percentage of ever married self-reported widowhood (widowhood*) and as reported by the head of the household (widowhood), in 2021, 2016, 2006,1999, and 1993 India.

	**15 to 44 years**				**45 to 49**				**50 to 54 years**				**Total**			
	Widowhood reported by Head of the Household		Self-reported Widowhood		Widowhood reported by Head of the Household		Self-reported Widowhood		Widowhood reported by Head of the Household		Self-reported Widowhood		Widowhood reported by Head of the Household		Self-reported Widowhood	
	Sample Size	Weighted Percentage	Sample Size	Weighted Percentage	Sample Size	Weighted Percentage	Sample Size	Weighted Percentage	Sample Size	Weighted Percentage	Sample Size	Weighted Percentage	Sample Size	Weighted Percentage	Sample Size	Weighted Percentage
**Male**																
2021	351,966	0.8	45,912	0.6	81,424	1.9	10,601	1.4	65,660	3.0	8,434	2.5	723,813	4.1	64,947	1.0
2016	363,714	0.8	52,439	0.7	76,176	2.0	10,950	2.2	60,551	3.4	8,460	2.6	704,598	4.1	71,849	1.1
2006	68,498	0.9	34,980	1.0	13,173	2.4	6,444	2.3	9,365	3.5	4,459	2.9	121,870	4.4	45,883	1.4
1999	67,864	1.1	.	.	11,429	2.5	.	.	8,696	4.8	.	.	115,324	4.8	.	.
1993	67,477	1.2	.	.	10,518	2.9	.	.	8,518	5.7	.	.	113,244	5.0	.	.
**Female**																
2021	487,520	2.7	459,696	2.8	85,749	10.8	83,134	11.2	70,724	16.1	.	.	869,214	14.3	5,42,830	4.1
2016	489,033	2.8	456,097	2.9	75,131	11.1	71,792	11.0	69,639	16.1	.	.	829,722	13.91	527,889	4.0
2006	93,422	3.1	84,031	3.3	11,028	12.0	9,693	11.1	10,983	19.9	.	.	145,501	14.5	93,724	4.1
1999	91,467	3.1	81,756	3.3	10,058	13.7	8,547	13.4	7,612	22.1	.	.	136,080	14.0	90,303	4.2
1993	91,113	2.8	80,999	3.0	9,789	12.6	8,507	12.6	7,021	22.2	.	.	134,352	13.5	89,777	3.8

Table S12: Prevalence (and 95% CI) of widowhood among ever-married men and women aged 75 years or above in 2016, 2006, and 1999 across states and union territories of India.

	Male			Female		
	2016	2006	1999	2016	2006	1999
India	28.2 (27.6,28.7)	29.4 (28.0,30.8)	31.0 (29.5,32.4)	77.5 (77.0,78.0)	82.1 (81.0,83.3)	81.9 (80.6,83.2)
**States**						
Andhra Pradesh	24.2 (19.8,28.5)	25.6 (19.1,32.1)	34.8 (24.4,45.2)	87.1 (84,90.2)	84.6 (79.6,89.6)	93.1 (87.2,99)
Arunachal Pradesh	32.8 (27.6,38.1)	31.3 (17.6,45)	37.4 (20.4,54.5)	71.2 (65.4,76.9)	66.8 (49.7,83.9)	83.4 (68.2,98.6)
Assam	24.2 (21.3,27.1)	29.8 (22,37.7)	13.9 (6.8,20.9)	84.8 (82.3,87.3)	90.5 (85,95.9)	84.1 (76,92.1)
Bihar	32.1 (30,34.2)	31.9 (23,40.8)	35.6 (28.8,42.5)	71 (68.8,73.2)	78.3 (71.1,85.5)	65.5 (57.7,73.3)
Chhattisgarh	32.0 (28.3,35.8)	32.1 (22.1,42.2)	30 (13.3,46.7)	80.6 (77.7,83.5)	80.8 (71.9,89.7)	89.4 (79.4,99.3)
Goa	25.7 (15.1,36.2)	22.1 (14.1,30.1)	21.5 (11,32)	81.1 (73.3,88.9)	78.2 (71.6,84.7)	90.5 (84.3,96.8)
Gujarat	26.9 (23.9,29.8)	34.7 (23.8,45.5)	39.9 (31.8,47.9)	71.6 (68.9,74.4)	87.3 (81.2,93.3)	79.7 (73.5,86)
Haryana	33.6 (30.2,36.9)	36.8 (27.9,45.7)	35.4 (27.1,43.8)	66.5 (63,70.1)	69 (60.4,77.6)	77.9 (69.9,85.9)
Himachal Pradesh	27 (23.7,30.3)	34.9 (26.7,43.1)	29.4 (22.7,36.2)	75.9 (73,78.9)	77.6 (70.6,84.6)	83.6 (77.7,89.4)
Jharkhand	32.3 (29.5,35.2)	30.4 (19.8,41)	41.1 (25.5,56.8)	78.8 (76.3,81.3)	76.2 (67.3,85.1)	77.1 (59.1,95.1)
Karnataka	19.3 (16.9,21.7)	24 (18.3,29.8)	22.8 (16.9,28.8)	83.1 (81,85.1)	87.7 (83.3,92.1)	87.4 (82.8,92)
Kerala	19.4 (16.3,22.5)	27.9 (20.8,35)	22.6 (16.2,29)	79.3 (76.6,82)	89.5 (85.5,93.4)	83.1 (77.8,88.3)
Madhya Pradesh	29.5 (27.6,31.4)	35.9 (29.3,42.6)	28.3 (23.1,33.4)	72.3 (70.5,74)	80.6 (75.1,86.2)	78.1 (72.7,83.5)
Maharashtra	24.9 (22.7,27.1)	22.5 (17.9,27)	31.3 (24.8,37.7)	80.3 (78.3,82.3)	84.3 (80.3,88.3)	86.6 (82,91.3)
Manipur	27.8 (24.4,31.3)	30.1 (23.6,36.7)	25.6 (15.3,35.9)	70.2 (66.7,73.7)	67.9 (60.8,74.9)	91.5 (85,98)
Meghalaya	29 (21.4,36.6)	21.8 (8.7,34.9)	43.1 (25,61.1)	74.5 (68.2,80.7)	85.3 (76,94.7)	85.4 (72,98.7)
Mizoram	22.6 (19,26.1)	26 (14.5,37.5)	33.5 (20.6,46.3)	65.7 (61.5,70)	70 (59.3,80.8)	65.2 (52.5,77.9)
Nagaland	21.4 (18.3,24.4)	19.8 (13.5,26)	24.9 (11.1,38.7)	58.9 (54.7,63.1)	63.6 (55.5,71.6)	78.3 (64.9,91.8)
Odisha	26.5 (24.3,28.8)	30 (23.3,36.7)	30.3 (24.1,36.6)	77.6 (75.4,79.7)	76.1 (69.1,83.2)	85.3 (80.2,90.4)
Punjab	40.6 (37.5,43.8)	33.7 (26.7,40.7)	30.2 (23.4,37.1)	70.5 (67.6,73.5)	73.2 (65.9,80.4)	72.2 (64.7,79.8)
Rajasthan	28.9 (26.7,31.1)	29.7 (21.1,38.3)	31.6 (25.9,37.2)	75.4 (73.5,77.3)	82.8 (75.7,89.9)	79 (73.9,84.1)
Sikkim	42.8 (36,49.7)	38.7 (25.8,51.6)	42.1 (26.2,58)	73 (65.8,80.2)	64.9 (51.1,78.7)	82 (67.3,96.8)
Tamil Nadu	23.8 (21.2,26.5)	27.5 (21.6,33.4)	30.2 (23.7,36.6)	76.3 (73.6,79)	86.1 (81.7,90.4)	89.4 (84.7,94.1)
Telangana	20.5 (16.1,25)	.	10.8 (2.5,19.1)	79.7 (75.4,83.9)	.	77.6 (65.3,89.9)
Tripura	19.1 (13.1,25)	18.1 (9.3,26.9)	21.7 (12.2,31.2)	87.8 (83.4,92.3)	87.4 (78.6,96.1)	84.8 (75.6,94)
Uttar Pradesh	38 (36.4,39.5)	36.7 (32.2,41.2)	39.9 (35.2,44.6)	73.6 (72.2,75)	74.4 (70,78.7)	75 (70.1,79.9)
Uttarakhand	28.5 (25.2,31.9)	27.4 (18.2,36.5)	20 (8.4,31.5)	77.7 (74.7,80.6)	80.7 (73.5,87.9)	80.1 (67.7,92.5)
West Bengal	21.2 (18.0,24.3)	18.9 (13.9,23.8)	23.0 (17.0,29.1)	88.4 (86.0,90.7)	90.0 (86.4,93.6)	90.1 (85.5,94.6)
**Union Territories**						
Andaman & Nicobar Islands (UT)	28.5 (19.4,37.6)	.	.	75.3 (64.9,85.7)	.	.
Chandigarh (UT)	29.1 (15.5,42.7)	.	.	66.5 (51.5,81.5)	.	.
Dadra & Nagar Haveli and daman & Diu (UT)	37.5 (23.2,51.8)	.	.	79.1 (69.3,88.9)	.	.
Jammu & Kashmir (UT)	31.5 (28.6,34.5)	25.6 (18.2,33)	35.2 (27.6,42.8)	68.8 (65.5,72)	80.7 (73.4,88.1)	76.6 (69,84.2)
Ladakh (UT)	46.8 (37.2,56.5)	.	.	52.8 (41.3,64.3)	.	.
Lakshadweep (UT)	15.5 (3.9,27)	.	.	77.1 (63.5,90.6)	.	.
NCT of Delhi (UT)	30.3 (23.6,37)	39.7 (29.4,49.9)	37.5 (27.8,47.3)	75.7 (69.2,82.3)	84 (76.5,91.5)	83.7 (75.7,91.8)
Puducherry (UT)	19.1 (12.4,25.9)	.	.	81.4 (75.6,87.3)	.	.

Table S13: Prevalence (and 95% CI) of widowhood among ever-married men and women aged 65 to 74 years in 2016, 2006, and 1999 across states and union territories of India.

	Male			Female		
	2016	2006	1999	2016	2006	1999
India	12.6 (12.4,12.9)	15.3 (14.6,16.0)	16.0 (15.3,16.8)	52.3 (51.9,52.7)	60.4 (59.4,61.4)	60.1 (59.1,61.2)
**States**						
Andhra Pradesh	11.5 (9.7,13.3)	14.5 (11.4,17.6)	19.8 (14.3,25.2)	64.2 (61.4,67)	67.1 (63,71.3)	72.2 (65.6,78.8)
Arunachal Pradesh	16.9 (14.2,19.5)	24.8 (15.4,34.3)	29.5 (20.6,38.4)	48.2 (43.9,52.4)	51.2 (38.6,63.7)	57.9 (45.7,70.1)
Assam	12.1 (10.7,13.6)	13.5 (9.2,17.8)	9.3 (5.4,13.2)	65.4 (63.1,67.7)	69.2 (63.1,75.2)	72.4 (65.1,79.8)
Bihar	15.1 (14,16.1)	16.1 (11.9,20.3)	17.8 (14.5,21)	45.2 (43.6,46.8)	59.8 (53.9,65.7)	49.5 (44.5,54.6)
Chhattisgarh	18.9 (17.1,20.8)	20.1 (15.3,25)	16.5 (9.5,23.5)	58.7 (56.4,61)	65.6 (60,71.3)	60 (50.8,69.3)
Goa	9.2 (4.7,13.8)	14.2 (10.0,18.4)	9.1 (3.8,14.4)	64.3 (57.3,71.3)	64.2 (59,69.4)	73.1 (66.5,79.7)
Gujarat	13.3 (11.8,14.8)	18.7 (14.1,23.4)	19.5 (15.2,23.8)	48.1 (46,50.2)	60 (54.1,66)	60.3 (55.1,65.5)
Haryana	14.4 (12.8,15.9)	19.8 (14.6,25.1)	21.4 (17.2,25.6)	41.1 (38.8,43.5)	49 (42.2,55.7)	45.7 (40,51.4)
Himachal Pradesh	9.5 (7.7,11.3)	15 (10.6,19.4)	15.8 (12,19.6)	47.1 (44.2,50)	61.4 (55.1,67.6)	53.9 (48.4,59.4)
Jharkhand	15 (13.6,16.3)	13.7 (9.1,18.3)	19.6 (12.1,27.1)	54.1 (52.1,56.1)	61.7 (54.2,69.1)	61.6 (50.1,73)
Karnataka	10 (8.9,11.2)	10.5 (7.6,13.4)	9.9 (6.9,12.8)	57.2 (55.4,59.1)	69.8 (65.7,73.8)	74.6 (70.5,78.7)
Kerala	7.2 (5.8,8.5)	6.0 (3.4,8.7)	9.3 (6,12.7)	53.4 (50.8,56)	68.6 (64.1,73.2)	60.3 (55.1,65.5)
Madhya Pradesh	13.9 (13,14.9)	15 (11.9,18.1)	16.1 (13.3,18.9)	48.7 (47.3,50.1)	55.5 (51.1,60)	52.9 (48.8,57)
Maharashtra	7.3 (6.5,8.2)	10.4 (8.3,12.5)	12.2 (9.4,14.9)	50.9 (49.4,52.5)	56.3 (52.8,59.7)	64 (60,68.1)
Manipur	9.8 (8.1,11.5)	12.8 (9.2,16.4)	16.7 (10.8,22.6)	46.6 (43.7,49.6)	49.2 (43.4,55)	60.5 (52.4,68.7)
Meghalaya	15.2 (11.5,18.9)	20 (12.1,27.9)	8 (2.6,13.4)	53.7 (48.1,59.3)	58.7 (49.1,68.2)	62 (50.2,73.8)
Mizoram	10.9 (8.9,12.9)	12.9 (6.7,19.2)	13.7 (7.1,20.3)	38.3 (35.1,41.5)	42.4 (32.8,52)	54.5 (45,64)
Nagaland	9.9 (8,11.7)	8.4 (4.8,12.0)	10.4 (4.2,16.5)	35.6 (32.2,38.9)	36.7 (29.4,44.1)	43 (31,54.9)
Odisha	12.6 (11.4,13.7)	13.3 (9.8,16.8)	15.7 (12.6,18.7)	50.1 (48.3,51.9)	62 (57.1,67)	60.9 (56.3,65.5)
Punjab	18.4 (16.8,20)	15.2 (11.4,18.9)	16.1 (12.9,19.4)	43.1 (40.9,45.3)	44.7 (39.5,50)	43.4 (38.4,48.3)
Rajasthan	11.2 (10.2,12.2)	13.4 (9.5,17.2)	18 (14.9,21)	45.8 (44.2,47.5)	50.7 (44.7,56.7)	59.7 (55.7,63.7)
Sikkim	20.1 (16,24.2)	21.9 (15.1,28.8)	24.4 (15.8,33)	41.8 (36.1,47.5)	56.3 (47.5,65.1)	56.4 (45.6,67.2)
Tamil Nadu	8.7 (7.6,9.8)	14 (11.0,17.0)	16 (12.7,19.3)	55.3 (53.3,57.4)	67.6 (63.5,71.7)	67.4 (63.3,71.6)
Telangana	11 (8.9,13)	.	11.5 (6.2,16.7)	57.1 (53.8,60.4)	.	62.9 (53.6,72.3)
Tripura	12.1 (8.7,15.5)	12 (6.1,17.9)	10.6 (4.9,16.4)	63.5 (58.4,68.6)	72.7 (65.4,80.0)	65.6 (56.1,75)
Uttar Pradesh	18.7 (17.8,19.5)	23.9 (21.3,26.5)	20.7 (18.1,23.4)	49 (47.8,50.1)	54.0 (50.8,57.3)	49.7 (46.1,53.4)
Uttarakhand	11.8 (10.2,13.4)	14.4 (10.2,18.5)	11.8 (5.6,18.1)	52.8 (50.3,55.3)	58 (51.7,64.3)	53.4 (43.6,63.2)
West Bengal	9.8 (8.3,11.4)	10.2 (7.6,12.8)	13.1 (10.1,16.2)	64.3 (61.7,66.8)	71.8 (67.9,75.7)	71.6 (67.2,76.1)
**Union Territories**						
Andaman & Nicobar Islands (UT)	19.9 (14.2,25.5)	.	.	64.6 (57.3,71.9)	.	.
Chandigarh (UT)	12.4 (4.6,20.3)	.	.	43.1 (31.9,54.2)	.	.
Dadra & Nagar Haveli and daman & Diu (UT)	8.8 (4.4,13.2)	.	.	54.9 (47.5,62.3)	.	.
Jammu & Kashmir (UT)	13 (11.5,14.5)	18.9 (14.1,23.6)	19 (14.8,23.2)	41.9 (39.4,44.3)	46.6 (39.8,53.3)	52.8 (46.3,59.3)
Ladakh (UT)	15.9 (10.9,20.9)	.	.	35.1 (27.3,42.9)	.	.
Lakshadweep (UT)	4.6 (0.3,9)	.	.	62.3 (53,71.6)	.	.
NCT of Delhi (UT)	19.2 (15.8,22.6)	13.2 (8.5,17.8)	12.7 (8.5,16.9)	44.1 (39.4,48.9)	49.9 (43.1,56.8)	46.7 (40,53.3)
Puducherry (UT)	11 (7.4,14.6)	.	.	65.4 (60.3,70.5)	.	.

Table S14: Prevalence (and 95% CI) of widowhood among ever-married men and women aged 45 to 64 years in 2016, 2006, and 1999 across states and union territories of India.

	Male			Female		
	2016	2006	1999	2016	2006	1999
India	4.1 (4.0,4.2)	5.0 (4.8,5.2)	5.9 (5.7,6.2)	19.3 (19.2,19.5)	24.3 (23.8,24.7)	26.6 (26.1.9,27.1)
**States**						
Andhra Pradesh	3.7 (3.1,4.3)	4.4 (3.5,5.2)	6.2 (4.5,7.8)	25.6 (24.4,26.9)	28.6 (26.7,30.4)	35.9 (32.7,39.1)
Arunachal Pradesh	5.3 (4.7,5.8)	7.4 (5.2,9.7)	15.2 (11.8,18.7)	18.9 (17.8,20)	25.2 (20.9,29.5)	23.1 (18.6,27.6)
Assam	4 (3.6,4.4)	4.8 (3.5,6)	3.9 (2.8,5)	26.2 (25.3,27.1)	29.4 (26.6,32.2)	31.2 (28.2,34.1)
Bihar	5.6 (5.2,5.9)	7.2 (5.6,8.7)	7.8 (6.6,9)	15.4 (14.9,16)	20.2 (17.8,22.5)	20.2 (18.3,22.1)
Chhattisgarh	5.3 (4.8,5.8)	6 (4.6,7.4)	8.6 (5.6,11.6)	20.2 (19.3,21)	25.7 (23.1,28.3)	24.1 (19.5,28.7)
Goa	2.2 (1.1,3.4)	2.2 (1.4,3)	5.3 (3.5,7.2)	24.6 (21.4,27.8)	28.2 (25.8,30.6)	32.7 (29.1,36.2)
Gujarat	4.4 (4,4.8)	5 (3.7,6.2)	6.5 (5.2,7.7)	16.9 (16.2,17.7)	22.5 (20,24.9)	24.8 (22.5,27.1)
Haryana	5.2 (4.7,5.7)	3.9 (2.6,5.3)	4.6 (3.3,5.9)	16.5 (15.7,17.3)	21.9 (19.1,24.8)	16.9 (14.5,19.2)
Himachal Pradesh	2.8 (2.3,3.4)	3.9 (2.8,5.1)	5.6 (4.3,6.9)	19.2 (18,20.3)	21.2 (18.7,23.7)	23.3 (21,25.6)
Jharkhand	5.5 (5.1,5.9)	5.7 (4.2,7.2)	6.5 (4.5,8.4)	20.4 (19.7,21.2)	27 (24.2,29.9)	25.4 (21.6,29.3)
Karnataka	2.6 (2.3,2.9)	2.4 (1.7,3.1)	4 (3,4.9)	25.7 (24.9,26.6)	31 (29,33)	30.8 (28.6,33.1)
Kerala	1.6 (1.3,2)	1.3 (0.7,2)	2.1 (1.3,2.9)	16.7 (15.8,17.6)	23.5 (21.3,25.8)	25.9 (23.5,28.4)
Madhya Pradesh	5.1 (4.8,5.4)	6.3 (5.3,7.4)	7.4 (6.2,8.6)	16.5 (16,17)	18.9 (17.1,20.6)	21.7 (19.9,23.6)
Maharashtra	2.2 (1.9,2.5)	3.1 (2.5,3.7)	3.4 (2.6,4.1)	20 (19.2,20.7)	22.5 (21,23.9)	28.9 (26.9,30.8)
Manipur	3.3 (2.8,3.8)	3.1 (2.1,4)	3.3 (1.8,4.8)	17.6 (16.5,18.6)	18.1 (16.1,20.1)	20.1 (16.9,23.3)
Meghalaya	4.2 (3.5,5)	4.6 (2.9,6.4)	4.3 (2.1,6.4)	24.3 (22.6,25.9)	30.2 (26.4,34)	29.5 (24.9,34.1)
Mizoram	4.2 (3.6,4.8)	3.1 (1.6,4.6)	5.1 (3.1,7.2)	16.5 (15.4,17.6)	14.6 (11.4,17.9)	21.2 (17.3,25.1)
Nagaland	2.7 (2.2,3.2)	4.9 (3.6,6.1)	4.4 (2.1,6.7)	15.7 (14.6,16.9)	19.2 (16.7,21.7)	23.8 (18.7,28.8)
Odisha	4.1 (3.7,4.4)	5.5 (4.3,6.7)	6.7 (5.5,7.9)	17.5 (16.8,18.2)	24.9 (22.6,27.2)	23.4 (21.4,25.4)
Punjab	5.4 (4.9,5.9)	5 (3.7,6.2)	5.5 (4.2,6.9)	16 (15.2,16.8)	15.6 (13.5,17.7)	17.2 (15,19.3)
Rajasthan	4.5 (4.2,4.8)	6.2 (4.8,7.6)	7.1 (6.1,8.1)	15.1 (14.5,15.7)	22.2 (19.8,24.6)	21.8 (20.2,23.5)
Sikkim	7.4 (6.2,8.6)	7.8 (5.7,9.9)	7.9 (5.3,10.5)	14.1 (12.4,15.8)	22.4 (18.8,25.9)	20 (15.6,24.4)
Tamil Nadu	2.7 (2.4,3)	3.3 (2.6,4.1)	5.1 (4.1,6.2)	21.3 (20.6,22.1)	29 (27.1,30.8)	32.1 (30,34.2)
Telangana	3.4 (2.7,4)	.	4.4 (2.6,6.2)	25.2 (23.7,26.8)	.	32.4 (28.4,36.4)
Tripura	1.6 (1,2.2)	2.0 (0.7,3.2)	3.7 (1.9,5.5)	22.8 (20.8,24.8)	31.5 (27.3,35.7)	31.9 (27.5,36.2)
Uttar Pradesh	6.6 (6.3,6.9)	8.1 (7.2,9)	9.8 (8.7,10.9)	16.5 (16.1,16.9)	20.8 (19.5,22.2)	21.9 (20.3,23.4)
Uttarakhand	4.4 (3.9,5)	4.3 (3,5.5)	5.2 (3.1,7.4)	22.4 (21.3,23.4)	26.2 (23.5,28.8)	24.5 (20.4,28.7)
West Bengal	2.7 (2.3,3.1)	2.9 (2.3,3.6)	3.2 (2.4,4.1)	22.5 (21.5,23.6)	29.6 (27.6,31.5)	32.4 (30.1,34.6)
**Union Territories**						
Andaman & Nicobar Islands (UT)	4.8 (3.5,6.1)	.	.	25.4 (22.6,28.2)	.	.
Chandigarh (UT)	5 (2.6,7.5)	.	.	16.5 (12.2,20.9)	.	.
Dadra & Nagar Haveli and daman & Diu (UT)	3 (1.8,4.3)	.	.	19 (16.3,21.8)	.	.
Jammu & Kashmir (UT)	4.1 (3.7,4.6)	4.2 (3,5.4)	6.5 (5.1,7.9)	13.8 (13,14.6)	18.1 (15.7,20.6)	21.9 (19.4,24.5)
Ladakh (UT)	4.9 (3.3,6.5)	.	.	11.3 (9,13.6)	.	.
Lakshadweep (UT)	2.9 (1.2,4.6)	.	.	20.1 (16.3,24)	.	.
NCT of Delhi (UT)	2.7 (2.1,3.4)	3.9 (2.8,5.1)	3 (1.9,4)	16 (14.5,17.4)	18.9 (16.4,21.5)	21.7 (19.1,24.4)
Puducherry (UT)	1.9 (1.1,2.6)	.	.	23.9 (21.7,26.1)	.	.

Table S15: Prevalence (and 95% CI) of widowhood among ever-married men and women aged less than 45 years in 2016, 2006, and 1999 across states and union territories of India.

	Male			Female		
	2016	2006	1999	2016	2006	1999
India	0.8 (0.8,0.8)	0.9 (0.8,1.0)	1.1 (1.0,1.2)	2.8 (2.8,2.9)	3.1 (3.0,3.2)	3.1 (3.0,3.2)
**States**						
Andhra Pradesh	0.7 (0.5,0.9)	0.3 (0.1,0.5)	0.8 (0.4,1.3)	4.7 (4.3,5.2)	4.2 (3.7,4.8)	4.5 (3.6,5.3)
Arunachal Pradesh	1.0 (0.8,1.3)	1.6 (0.8,2.4)	2.5 (1.5,3.4)	2.5 (2.2,2.8)	3.6 (2.5,4.7)	4.6 (3.4,5.8)
Assam	0.8 (0.6,0.9)	0.6 (0.2,1)	1 (0.6,1.4)	3 (2.7,3.2)	4.5 (3.7,5.2)	3.9 (3.2,4.6)
Bihar	0.8 (0.6,0.9)	1.6 (1,2.1)	1.3 (0.9,1.7)	1.8 (1.7,1.9)	2.6 (2.1,3.2)	2.4 (2,2.8)
Chhattisgarh	1.0 (0.8,1.2)	0.8 (0.5,1.2)	1.1 (0.4,1.9)	3.1 (2.8,3.3)	4.3 (3.5,5)	2.7 (1.7,3.8)
Goa	0.1 (0.0,0.0)	0.2 (0,0.4)	0.3 (0,0.7)	3.5 (2.3,4.6)	4.4 (3.6,5.2)	3.6 (2.5,4.6)
Gujarat	0.9 (0.7,1.1)	0.7 (0.4,1.1)	1.5 (1,1.9)	2.3 (2.1,2.5)	2.4 (1.8,3)	3.3 (2.7,3.8)
Haryana	1.1 (0.9,1.3)	0.5 (0.1,0.8)	1.3 (0.8,1.8)	2.3 (2.1,2.5)	3.3 (2.6,4.1)	2.3 (1.7,2.8)
Himachal Pradesh	0.6 (0.4,0.8)	0.4 (0.1,0.8)	1 (0.5,1.4)	2.2 (1.9,2.6)	2.4 (1.7,3)	2.3 (1.8,2.9)
Jharkhand	0.6 (0.5,0.8)	1.5 (0.9,2.1)	1.6 (0.9,2.3)	2.7 (2.5,2.9)	3.2 (2.5,3.9)	2.8 (2,3.6)
Karnataka	0.3 (0.2,0.4)	0.5 (0.3,0.8)	0.5 (0.2,0.8)	4.3 (4,4.6)	4.2 (3.6,4.7)	4.2 (3.6,4.8)
Kerala	0.2 (0.1,0.4)	0.2 (0,0.4)	0 (0,0)	1.9 (1.6,2.2)	1.9 (1.4,2.4)	2.1 (1.6,2.7)
Madhya Pradesh	1.1 (1,1.2)	1.5 (1.1,1.9)	1.5 (1.2,1.9)	2.2 (2,2.3)	2.8 (2.3,3.2)	2.1 (1.8,2.5)
Maharashtra	0.4 (0.3,0.5)	0.2 (0.1,0.3)	0.7 (0.5,1)	3.7 (3.4,3.9)	3.5 (3.1,3.9)	4 (3.5,4.5)
Manipur	0.4 (0.3,0.6)	0.4 (0.1,0.7)	0.9 (0.3,1.5)	3.2 (2.9,3.6)	3.3 (2.6,3.9)	3.3 (2.3,4.2)
Meghalaya	0.5 (0.3,0.8)	1.6 (0.8,2.4)	1.3 (0.5,2.1)	2.8 (2.3,3.2)	4.2 (3.1,5.3)	2.7 (1.7,3.7)
Mizoram	1.1 (0.8,1.3)	1.2 (0.5,2)	1 (0.3,1.6)	5 (4.5,5.5)	3.7 (2.6,4.8)	4.0 (2.8,5.2)
Nagaland	0.8 (0.6,1.1)	0.7 (0.3,1.1)	1.8 (0.7,2.9)	3.3 (2.9,3.8)	2.6 (2,3.3)	2.5 (1.4,3.6)
Odisha	0.5 (0.4,0.6)	0.9 (0.5,1.3)	1 (0.6,1.3)	3 (2.7,3.2)	3.2 (2.6,3.8)	2.9 (2.4,3.4)
Punjab	0.8 (0.6,0.9)	0.6 (0.3,0.9)	1 (0.6,1.4)	2.4 (2.1,2.7)	3.1 (2.5,3.8)	2.5 (1.9,3.1)
Rajasthan	0.8 (0.7,0.9)	1 (0.6,1.4)	1.4 (1.1,1.7)	1.9 (1.8,2.1)	2.4 (1.8,2.9)	1.7 (1.4,2)
Sikkim	1.0 (0.6,1.4)	0.8 (0.3,1.3)	1.1 (0.4,1.8)	2.2 (1.7,2.7)	2.2 (1.4,3)	1.8 (1,2.5)
Tamil Nadu	0.4 (0.3,0.5)	0.3 (0.1,0.6)	0.7 (0.4,1)	3.2 (3,3.5)	4.1 (3.5,4.7)	4.8 (4.1,5.4)
Telangana	0.5 (0.3,0.7)	.	0.1 (0,0.4)	5.2 (4.6,5.7)	.	3.1 (2.2,3.9)
Tripura	0.4 (0.2,0.7)	0.2 (0,0.5)	0.8 (0.2,1.5)	3 (2.4,3.6)	3.2 (2.3,4.1)	4.2 (3.1,5.4)
Uttar Pradesh	1.4 (1.3,1.5)	1.6 (1.3,1.9)	1.8 (1.5,2.2)	2 (1.9,2.1)	2.1 (1.8,2.4)	2.2 (1.9,2.5)
Uttarakhand	0.5 (0.4,0.7)	0.9 (0.4,1.4)	1.0 (0.3,1.8)	2.8 (2.5,3.1)	2.6 (1.9,3.2)	3.1 (2,4.1)
West Bengal	0.5 (0.3,0.6)	0.3 (0.1,0.5)	0.5 (0.2,0.8)	2.4 (2.1,2.7)	3.2 (2.7,3.7)	3.4 (2.9,4)
**Union Territories**						
Andaman & Nicobar Islands (UT)	0.2 (0,0.5)	.	.	2.8 (2,3.5)	.	.
Chandigarh (UT)	0.1 (0,0.5)	.	.	1.2 (0.2,2.1)	.	.
Dadra & Nagar Haveli and daman & Diu (UT)	0.2 (0,0.5)	.	.	1.9 (1.2,2.6)	.	.
Jammu & Kashmir (UT)	0.6 (0.4,0.7)	1 (0.5,1.5)	1 (0.6,1.4)	1.4 (1.2,1.6)	1.5 (1,2.1)	2.2 (1.7,2.7)
Ladakh (UT)	0.3 (0,0.8)	.	.	0.9 (0.3,1.5)	.	.
Lakshadweep (UT)	0.9 (0,1.7)	.	.	2.1 (1.1,3.2)	.	.
NCT of Delhi (UT)	0.3 (0.1,0.5)	0.6 (0.3,0.9)	1 (0.5,1.4)	1.8 (1.4,2.2)	2.6 (2,3.3)	2.7 (2,3.3)
Puducherry (UT)	0.0 (0.0,0.1)	.	.	5 (4.1,5.8)	.	.

Figure S1: Summary distribution of State/Union Territories- level prevalence of widowhood among ever married men and women 2016, 2006, 1999.

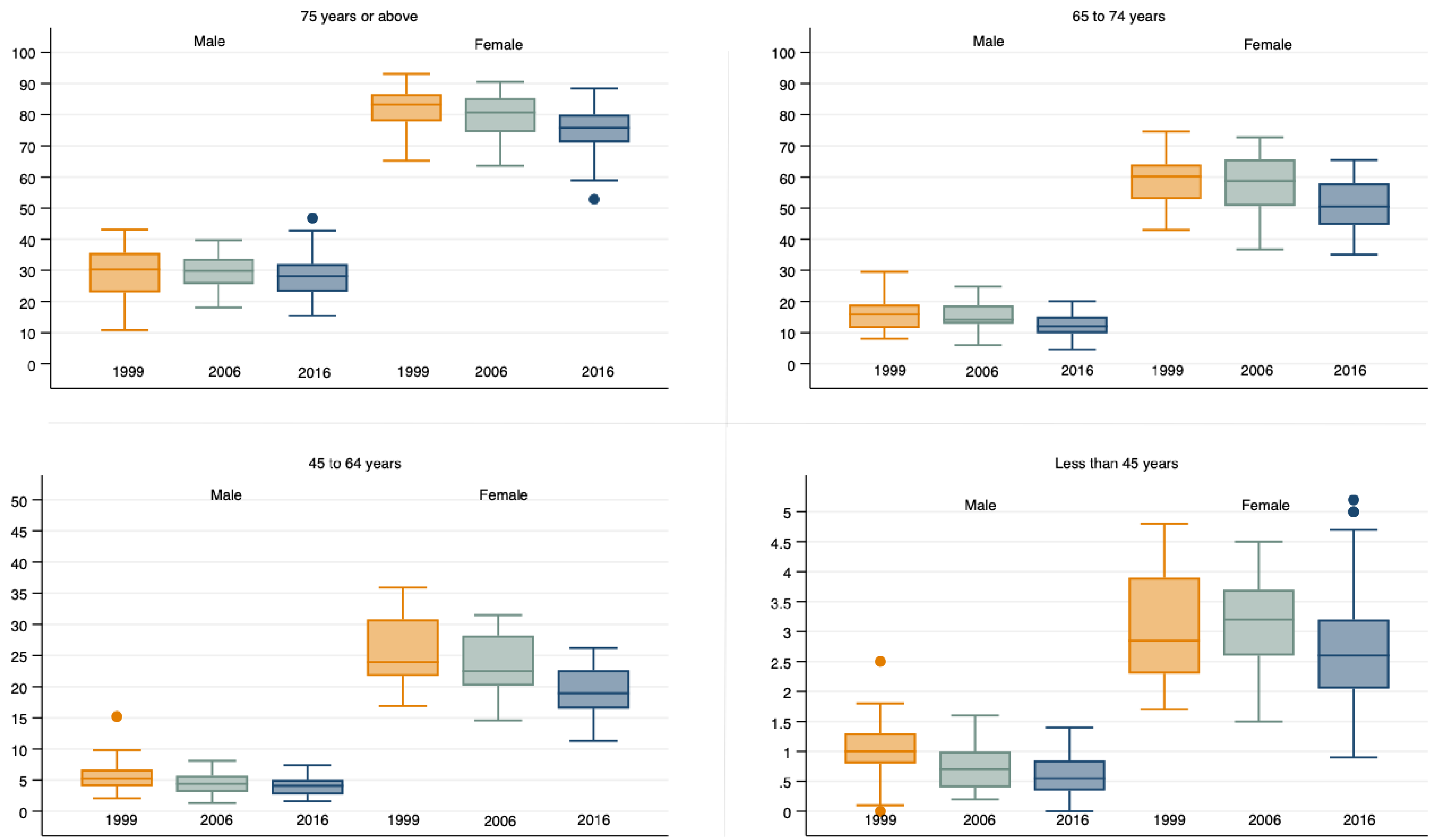

Figure S2: Relationship between Prevalence of widowhood (%) and its headcount (N) among ever-married women across states and union territories.

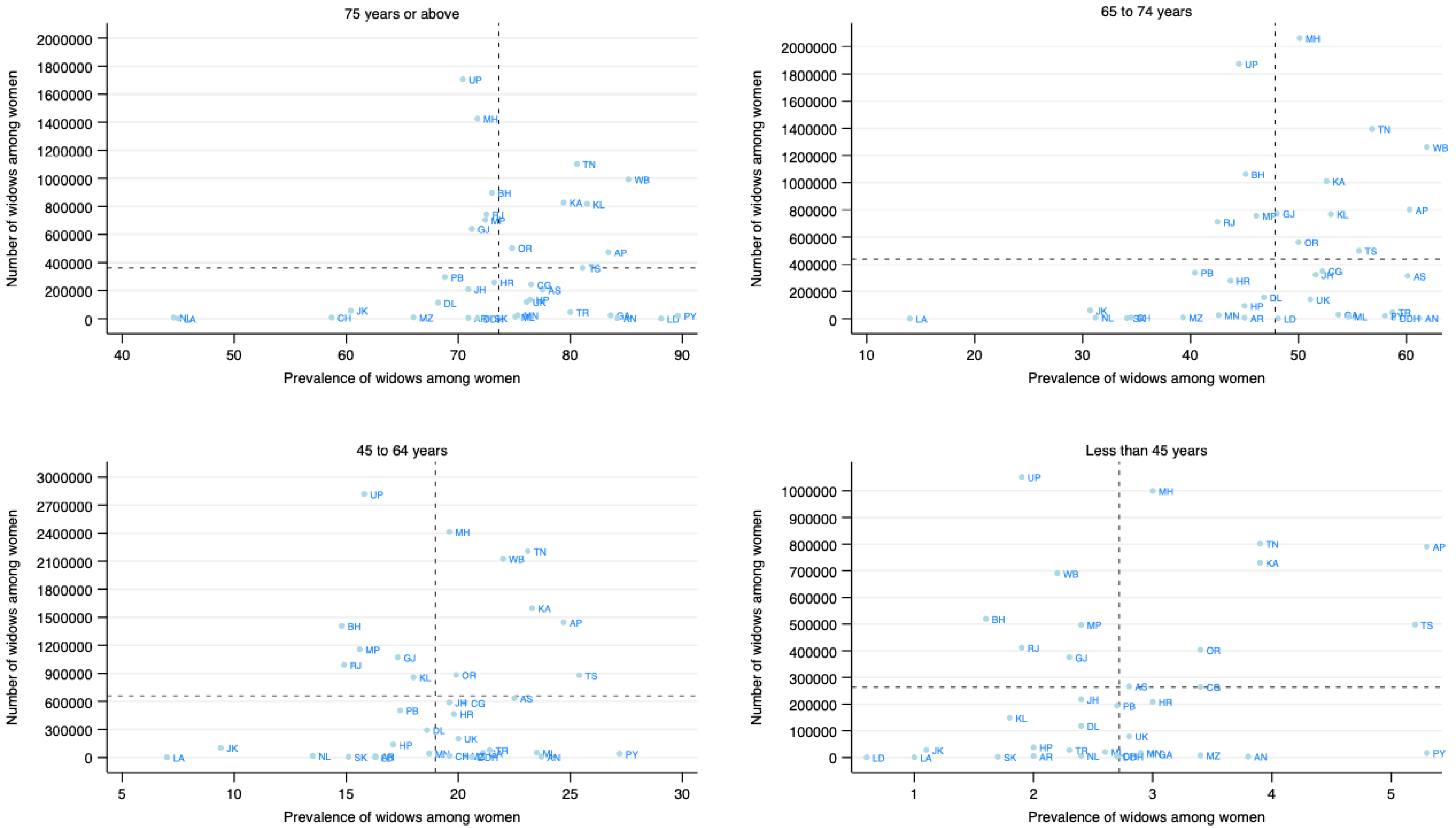

Text S1: National Family Health Survey (NFHS): summary of survey design.

NFHS-1 (1992-1993)
The sample size consisted of 3000 eligible women for states with a population of 25 million or less in 1991, 4000 for the largest states, and 1000 each for the six north-eastern states (Arunachal Pradesh, Manipur, Meghalaya, Mizoram, Nagaland, and Tripura). Separate samples for urban and rural areas were drawn in proportion to the population size. Villages were stratified before selection based on various factors such as size, distance from the nearest town, proportion of non-agricultural workers, SC/ST population, and female literacy. However, not each variable was used for all states. A house listing was conducted in all selected villages. For urban areas, a three-stage sampling design was implemented: (1) Selection of self-selecting cities (with large populations) (2) Selection of towns that are district headquarters, (3) Selection of other towns. Within the selected towns, two census blocks were chosen, followed by the selection of households within each block. Three stage sample design was used: selection of towns with PPS, followed by selection of two census blocks per selected towns, followed by selection households in each selected block.

NFHS-2 (1998-1999) and NFHS-3 (2005-2006)
The National Family Health Survey (NFHS-2) encompassed over 99 percent of India's population across all 26 states, excluding the union territories. This household survey targeted approximately 90,000 ever-married women aged 15–49. The sample size was determined based on the state's population size, available resources, and the need for estimates at aggregate levels, including urban/rural, region, and metropolitan cities. The initial target sample size was 4,000 completed interviews with eligible women in states with a 1991 population of more than 25 million, 3,000 completed interviews with eligible women in states with a 1991 population between 2 and 25 million, and 1,500 completed interviews with eligible women in states with a population of less than 2 million. A uniform sample design was adopted in all the states. In each state, the rural sample was selected in two stages: the selection of Primary Sampling Units (PSUs), which are villages, with probability proportional to population size (PPS) at the first stage, followed by the random selection of 9 households within each PSU in the second stage. In urban areas, a three-stage procedure was followed. In the first stage, wards were selected with PPS sampling. In the next stage, one census enumeration block (CEB) was randomly selected from each sample ward (except in Jammu and Kashmir, where two CEBs were randomly selected from each sample ward). In the final stage, households were randomly selected within each sample CEB. For NFHS-3, the sampling frame was based on the 2001 census data.

NFHS-4 (2015-2016) and NFHS-5 (2019-2021)

The NFHS-4 and NFHS-5 surveys were designed to provide district-level estimates, utilizing a stratified two-stage sampling method. The 2011 census served as the sampling frame for the selection of Primary Sampling Units (PSUs), which were defined as villages in rural areas and Census Enumeration Blocks (CEBs) in urban areas. PSUs with fewer than 40 households were combined with the nearest PSU. In rural areas, villages were selected using probability proportional to size (PPS) sampling within each stratum. Each rural stratum was divided into six approximately equal substrata, created by crossing three substrata based on the estimated number of households in each village with two substrata based on the percentage of the population belonging to scheduled castes and scheduled tribes (SCs/STs). PSUs within each explicit stratum were sorted by the literacy rate of women aged 6 years and above, and final PSUs were selected using PPS sampling. In urban areas, CEB information was obtained from the Office of the Registrar General and Census Commissioner in New Delhi. CEBs were sorted by the percentage of the SC/ST population, and sample CEBs were selected using PPS sampling. Before the main survey, a complete household mapping and listing operation was conducted in each selected rural and urban PSU. PSUs with at least 300 households were segmented into segments of approximately 100-150 households. Two of the segments were randomly selected for the survey using systematic sampling with probability proportional to segment size. Therefore, an NFHS-4 cluster is either a PSU or a segment of a PSU. In the second stage, in every selected rural and urban cluster, 22 households were randomly selected with systematic sampling.

Text S2: Methodology used for calculating the population headcount of widowhood.

To calculate the headcount, we followed Integrated Public Use Microdata Series Population Weight (IPUMS POPWT) methodology[1] with a few adjustments to the formulas.

Age Groupings	Male Value	Female Value	Tables Census Projections 2001, 2011	Source
13 to 44	370,384,000 (NFHS5); 351,778,000 (NFHS4); 302,508,000 (NFHS3); 267,130,143 (NFHS2); 211,080,150 (NFHS1)	345,933,000 (NFHS5); 330,207,000 (NFHS4); 281,723,000 (NFHS3); 249,343,800 (NFHS2); 196,632,176 (NFHS1)	The following tables were used: Table 18 & 20 (2006;2016;2021)	Census of India 199, 2001(NFHS2; NFHS1); Census 2001 Projections (NFHS3); Census 2011 Projections (NFHS4; NFHS5)
45 to 64	126,986,000 (NFHS5); 110,876,000 (NFHS4); 85,166,000 (NFHS3); 71,888,863 (NFHS2); 58,708,435 (NFHS1)	124,375,000 (NFHS5); 106,718,000 (NFHS4); 78,550,000 (NFHS3); 67,277,798 (NFHS2); 52,760,433 (NFHS1)	The following tables were used: Table 18 & 20 (2006;2016;2021)	Census of India 199, 2001(NFHS2; NFHS1); Census 2001 Projections (NFHS3); Census 2011 Projections (NFHS4; NFHS5)
65 to 74	29,369,000 (NFHS5); 26,200,000 (NFHS4); 18,878,000 (NFHS3); 16,999,791 (NFHS2); 12,029,580 (NFHS1)	31,487,000 (NFHS5); 27,837,000 (NFHS4); 20,488,000 (NFHS3); 17,515,808 (NFHS2); 11,383,000 (NFHS1)	The following tables were used: Table 18 & 20 (2006;2016;2021)	Census of India 199, 2001(NFHS2; NFHS1); Census 2001 Projections (NFHS3); Census 2011 Projections (NFHS4; NFHS5)
75 or above	14,906,000 (NFHS5); 12,199,000 (NFHS4); 8,144,000 (NFHS3); 7,182,189 (NFHS2); 5,426,908 (NFHS1)	17,264,000 (NFHS5); 13,528,000 (NFHS4); 8,628,000 (NFHS3); 7,407,754 (NFHS2); 5,093,176 (NFHS1)	The following tables were used: Table 18 & 20 (2006;2016;2021)	Census of India 199, 2001(NFHS2; NFHS1); Census 2001 Projections (NFHS3); Census 2011 Projections (NFHS4; NFHS5)

After deriving the 13-44 years, 45-64 years, 65-74 years and 75 years or above population for all-India, this paper used the formula given by IPUMS to derive the population weight (POPWT) which was used to calculate the headcount.

$$popwt = \left(\frac{wt}{sum(wt)}\right) \times Pop$$

- "*wt*" represents the individual weight divided by 1,000,000 in the DHS microdata.
- "*sum(wt)*" represents the total value of wt
- " *Pop*" represents the total all-India estimated for given gender and age- group.

Weighting the data using popwt instead of the given individual weights, this paper was able to derive the widowhood estimates for all-India and its states/union territories.

References

1. IPUMS DHS. POPWT Expansion factor. [Internet]. September 9, 2022. Available from: https://www.idhsdata.org/idhs/population_weights.shtml

2. National Commission on Population, Ministry of Health and Family Welfare, Government of India. Population Projections for India and States 2011-2036: Report of the Technical Group on Population Projections. [Internet]. 2020: Available from: https://main.mohfw.gov.in/sites/default/files/Population%20Projection%20Report%202011-2036%20-%20upload_compressed_0.pdf

3. Office of the Registrar General & Census Commissioner, India, Ministry of Home Affairs, Government of India. 1993 [Internet]. 1991: Available From : https://censusindia.gov.in/census.website/data/census-tables#

Text S3:

(A) Construction of widowhood Variables

We searched for the keyword "marital", and "married" in the stata file of NFHS individual level data (person file). For this search, we found hv115, sh08, ha60, hb60, hv020 and hv116 as variables that offer information about marital status of individuals. Out of the forementioned variables, ha60 and hb60 provided information only under three categories of - never in union, others and missing. The variables sh08, and hv115 provided information on marital status under wider categories of "never married, currently married, married but gauna not performed, widowed, divorced, separated, deserted, don't know, and missing", and "never married, married, widowed, divorced, not living together, don't know, and missing", respectively.

In contrast to sh08, the in the variable hv115, the information of never married and married but gauna not performed is combined as never married. Also, unlike sh08, the category of "not living together" in the variable hv115 includes information of divorced and separated, both.

Therefore, except for these differences in categorisation of marital status and partnership; there is no difference between the variables sh08 and hv115. Most essentially, the both provide "widowhood" as a separate distinct category; offering the same relevance to computationally derive widowhood prevalence,

The variable hv020 contains the information on ever-married sample. The variable hv116 contains information on (currently, formerly, never married, and missing). Whereas, the variable hv116 combines information of don't (hv115) know and missing from hv115 and classifies it as missing.

We dropped the don't know, missing and never married samples for the analysis of the widowhood in the study using the variables hv116. In the remaining complete case ever married sample, we create a dichotomous variable of "widowhood" that takes the vale 1 in case the reported marital status of the individual is widowed and 0 otherwise.

The variable sh08 is available as hs09 in NFHS2 (1999) and is not available in NFHS1 (1993).

(B) The sensitivity analysis

The sensitivity analysis is performed to check whether there is discrepancy in the self-reported marital status and the marital status reported by the head of the household in the reproductive ages (15 to 49 for females and 15-54 for males). To check this we used the variables s301, and v501 in IR file; and sm213, and mv501 in MR file. We found no difference between the reporting in the s301 and v501 in IR file, and sm213 and mv501 in MR file. Thereafter, we combined the two file with the PR file. And generated a new variable widow1, we found there is negligible difference in self-reported marital status and the marital status reported by the head of the household.

Text S4: STATA codes to estimate Widowhood Prevalence and Headcount across States and Union Territories of India 1993-2021.

```
*** NFHS1***
use "/Volumes/PAL007/Paper/Widowhood/NFHS1- reclustered_PR_file.dta", clear
tab1 hv115 hv116, m
tab1 hv115 hv116, nol m
drop if (hv116==0 | hv116==3 | hv116==.)
ge wt= hv005/1000000
recode hv115 (3=1 "widowed") (.=.) (else=0 "not widowed"), ge(widow)
drop if hv104==.
drop if (hv105==6 | hv105==7 |hv105==8 |hv105==9 |hv105==10 |hv105==11 |hv105==12)
recode hv105 (13/44=1 "less than 45") (45/64=2 "45 t0 64") (65/74=3 "65 to 74") (75/max=4 "75+") (else=.),
ge(age_grp)
ge v=.
replace v=1 if age_grp==1 & hv104==1
replace v=2 if age_grp==2 & hv104==1
replace v=3 if age_grp==3 & hv104==1
replace v=4 if age_grp==4 & hv104==1
replace v=5 if age_grp==1 & hv104==2
replace v=6 if age_grp==2 & hv104==2
replace v=7 if age_grp==3 & hv104==2
replace v=8 if age_grp==4 & hv104==2
ta v
ge pop=.
replace pop= 211080150 if v==1
replace pop= 58708435 if v==2
replace pop= 12029580 if v==3
replace pop= 5426908 if v==4
replace pop= 196632176 if v==5
replace pop= 52760433 if v==6
replace pop= 11383000 if v==7
replace pop= 5093176 if v==8
ta pop
ta v
egen sumwt = sum(wt), by(v)
ge popwt= (wt/sumwt)*pop

save "/Users/IITMandi/Downloads/Widowhood_NFHS1_5June2024.dta"

***NFHS2***
use "/Volumes/PAL007/Paper/Widowhood/NFHS2- reclustered_PR_file.dta", clear
tab1 hv115 hv116, m
tab1 hv115 hv116, nol m
drop if (hv116==0 | hv116==.)
ge wt= hv005/1000000
recode hv115 (3=1 "widowed") (.=.) (else=0 "not widowed"), ge(widow)
drop if (hv105==6 | hv105==7 |hv105==8 |hv105==9 |hv105==10 |hv105==11 |hv105==12)
drop if hv105==.
recode hv105 (13/44=1 "less than 45") (45/64=2 "45 t0 64") (65/74=3 "65 to 74") (75/max=4 "75+") (else=.),
ge(age_grp)
ge v=.
replace v=1 if age_grp==1 & hv104==1
replace v=2 if age_grp==2 & hv104==1
replace v=3 if age_grp==3 & hv104==1
replace v=4 if age_grp==4 & hv104==1
replace v=5 if age_grp==1 & hv104==2
replace v=6 if age_grp==2 & hv104==2
replace v=7 if age_grp==3 & hv104==2
replace v=8 if age_grp==4 & hv104==2
ta v
ge pop=.
replace pop= 267130143 if v==1
replace pop= 71888863 if v==2
replace pop= 16999791 if v==3
replace pop= 7182189 if v==4
replace pop= 249343800 if v==5
replace pop= 67277798 if v==6
```

```
replace pop= 17515808 if v==7
replace pop= 7407754 if v==8
ta pop
ta v
egen sumwt = sum(wt), by(v)
ge popwt= (wt/sumwt)*pop

save "/Users/IITMandi/Downloads/Widowhood_NFHS2_5June2024.dta"

***NFHS3***
use "/Volumes/PAL007/NFHS DATA/NFHS3/DATA IN STATA FORMAT/IAPR50DT/IAPR50FL.DTA", clear
tab1 hv115 hv116, m
tab1 hv115 hv116, nol m
drop if (hv116==0 | hv116==.)
ge wt= hv005/1000000
recode hv115 (3=1 "widowed") (.=.) (else=0 "not widowed"), ge(widow)
drop if (hv105==10 | hv105==11 | hv105==12 |hv105==98 |hv105==.)
recode hv105 (13/44=1 "less than 45") (45/64=2 "45 t0 64") (65/74=3 "65 to 74") (75/max=4 "75+") (else=.),
ge(age_grp)
ge v=.
replace v=1 if age_grp==1 & hv104==1
replace v=2 if age_grp==2 & hv104==1
replace v=3 if age_grp==3 & hv104==1
replace v=4 if age_grp==4 & hv104==1
replace v=5 if age_grp==1 & hv104==2
replace v=6 if age_grp==2 & hv104==2
replace v=7 if age_grp==3 & hv104==2
replace v=8 if age_grp==4 & hv104==2
ta v
ge pop=.
replace pop= 302508000 if v==1
replace pop= 85166000 if v==2
replace pop= 18878000 if v==3
replace pop= 8144000 if v==4
replace pop= 281723000 if v==5
replace pop= 78550000 if v==6
replace pop= 20488000 if v==7
replace pop= 8628000 if v==8
ta pop
ta v
egen sumwt = sum(wt), by(v)
ge popwt= (wt/sumwt)*pop

save "/Users/IITMandi/Downloads/Widowhood_NFHS3_5June2024.dta"

***NFHS4***
use "/Volumes/PAL007/NFHS DATA/nfhs4/stata/IAPR71DT/IAPR71FL.DTA", clear
replace hv024=37 if shdistri==3 | shdistri==4
label define hv024 37 "ladakh", add
replace hv024=38 if hv024==8 | hv024==9
label define hv024 38 "dadra & nagar haveli and daman & diu", add
label define hv024 8 "", modify
label define hv024 9 "", modify
tab1 hv115 hv116, m
tab1 hv115 hv116, nol m
drop if (hv116==0 | hv116==.)
ge wt= hv005/1000000
recode hv115 (3=1 "widowed") (.=.) (else=0 "not widowed"), ge(widow)
recode hv105 (13/44=1 "less than 45") (45/64=2 "45 t0 64") (65/74=3 "65 to 74") (75/max=4 "75+") (else=.),
ge(age_grp)
ge v=.
replace v=1 if age_grp==1 & hv104==1
replace v=2 if age_grp==2 & hv104==1
replace v=3 if age_grp==3 & hv104==1
replace v=4 if age_grp==4 & hv104==1
replace v=5 if age_grp==1 & hv104==2
replace v=6 if age_grp==2 & hv104==2
replace v=7 if age_grp==3 & hv104==2
replace v=8 if age_grp==4 & hv104==2
ta v
```

```
ge pop=.
replace pop= 351778000 if v==1
replace pop= 110876000 if v==2
replace pop= 26200000 if v==3
replace pop= 12199000 if v==4
replace pop= 330207000 if v==5
replace pop= 106718000 if v==6
replace pop= 27837000 if v==7
replace pop= 13528000 if v==8
ta pop
ta v
egen sumwt = sum(wt), by(v)
ge popwt= (wt/sumwt)*pop

save "/Users/IITMandi/Downloads/Widowhood_NFHS4_5June_2024.dta"

***NFHS5***
use "/Volumes/PAL007/NFHS DATA/NFHS 5/IAPR7AFL.DTA", clear
tab1 hv115 hv116, m
tab1 hv115 hv116, nol m
drop if (hv116==0 | hv116==.)
ge wt= hv005/1000000
recode hv115 (3=1 "widowed") (.=.) (else=0 "not widowed"), ge(widow)
recode hv105 (13/44=1 "less than 45") (45/64=2 "45 t0 64") (65/74=3 "65 to 74") (75/max=4 "75+") (else=.),
ge(age_grp)
ge v=.
replace v=1 if age_grp==1 & hv104==1
replace v=2 if age_grp==2 & hv104==1
replace v=3 if age_grp==3 & hv104==1
replace v=4 if age_grp==4 & hv104==1
replace v=5 if age_grp==1 & hv104==2
replace v=6 if age_grp==2 & hv104==2
replace v=7 if age_grp==3 & hv104==2
replace v=8 if age_grp==4 & hv104==2
ta v
ge pop=.
replace pop= 370384000 if v==1
replace pop= 126986000 if v==2
replace pop= 29369000 if v==3
replace pop= 14906000 if v==4
replace pop= 345933000 if v==5
replace pop= 124375000 if v==6
replace pop= 31487000 if v==7
replace pop= 17264000 if v==8
ta pop
ta v
egen sumwt = sum(wt), by(v)
ge popwt= (wt/sumwt)*pop
save "/Users/IITMandi/Downloads/Widowhood_NFHS5_5June2024.dta"


***Table1*** (sample size(n) and unweighted percentage of widows)

///NFHS5///
use "/Users/IITMandi/Downloads/Widowhood_NFHS5_5June2024.dta", clear
ta age_grp widow if hv104==1
ta age_grp widow if hv104==1, r nof
ta age_grp widow if hv104==2
ta age_grp widow if hv104==2, r nof

///NFHS4///
use "/Users/IITMandi/Downloads/Widowhood_NFHS4_5June_2024.dta", clear
ta age_grp widow if hv104==1
ta age_grp widow if hv104==1, r nof
ta age_grp widow if hv104==2
ta age_grp widow if hv104==2, r nof

///NFHS3///
use "/Users/IITMandi/Downloads/Widowhood_NFHS3_5June2024.dta", clear
ta age_grp widow if hv104==1
```

```
ta age_grp widow if hv104==1, r nof
ta age_grp widow if hv104==2
ta age_grp widow if hv104==2, r nof

///NFHS2///
use "/Users/IITMandi/Downloads/Widowhood_NFHS2_5June2024.dta", clear
ta age_grp widow if hv104==1
ta age_grp widow if hv104==1, r nof
ta age_grp widow if hv104==2
ta age_grp widow if hv104==2, r nof

///NFHS1///
use "/Users/IITMandi/Downloads/Widowhood_NFHS1_5June2024.dta", clear
ta age_grp widow if hv104==1
ta age_grp widow if hv104==1, r nof
ta age_grp widow if hv104==2
ta age_grp widow if hv104==2, r nof

***Table2***

///NFHS5///
///Prelavence (95%CI)///
use "/Users/IITMandi/Downloads/Widowhood_NFHS5_5June2024.dta", clear
mean widow [aw=wt] if hv104==1, over (age_grp)
mean widow [aw=wt] if hv104==2, over (age_grp)
///Headcoun (N)///
ta age_grp widow[iw=popwt] if hv104==1
ta age_grp widow[iw=popwt] if hv104==2

///NFHS4///
///Prelavence (95%CI)///
use "/Users/IITMandi/Downloads/Widowhood_NFHS4_5June_2024.dta", clear
mean widow [aw=wt] if hv104==1, over (age_grp)
mean widow [aw=wt] if hv104==2, over (age_grp)
///Headcoun (N)///
ta age_grp widow[iw=popwt] if hv104==1
ta age_grp widow[iw=popwt] if hv104==2

///NFHS3///
///Prelavence (95%CI)///
use "/Users/IITMandi/Downloads/Widowhood_NFHS3_5June2024.dta", clear
mean widow [aw=wt] if hv104==1, over (age_grp)
mean widow [aw=wt] if hv104==2, over (age_grp)
///Headcoun (N)///
ta age_grp widow[iw=popwt] if hv104==1
ta age_grp widow[iw=popwt] if hv104==2

///NFHS2///
///Prelavence (95%CI)///
use "/Users/IITMandi/Downloads/Widowhood_NFHS2_5June2024.dta", clear
mean widow [aw=wt] if hv104==1, over (age_grp)
mean widow [aw=wt] if hv104==2, over (age_grp)
///Headcoun (N)///
ta age_grp widow[iw=popwt] if hv104==1
ta age_grp widow[iw=popwt] if hv104==2

///NFHS1///
///Prelavence (95%CI)///
use "/Users/IITMandi/Downloads/Widowhood_NFHS1_5June2024.dta", clear
mean widow [aw=wt] if hv104==1, over (age_grp)
mean widow [aw=wt] if hv104==2, over (age_grp)

///Headcoun (N)///
ta age_grp widow[iw=popwt] if hv104==1
ta age_grp widow[iw=popwt] if hv104==2

***Table3***
///Prevalence of widowhood (95% CI) among ever-married men and women in 2021, and 1993, across states and
union territories of India///
```

```
///NFHS5///
use "/Users/IITMandi/Downloads/Widowhood_NFHS5_5June2024.dta", clear
// 1 IS MALE //
mean widow [aw=wt] if (hv104==1 & age_grp==1), over(hv024)
mean widow [aw=wt] if (hv104==1 & age_grp==2), over(hv024)
mean widow [aw=wt] if (hv104==1 & age_grp==3), over(hv024)
mean widow [aw=wt] if (hv104==1 & age_grp==4), over(hv024)
// 2 IS FEMALE //
mean widow [aw=wt] if (hv104==2 & age_grp==1), over(hv024)
mean widow [aw=wt] if (hv104==2 & age_grp==2), over(hv024)
mean widow [aw=wt] if (hv104==2 & age_grp==3), over(hv024)
mean widow [aw=wt] if (hv104==2 & age_grp==4), over(hv024)

///NFHS1///
use "/Users/IITMandi/Downloads/Widowhood_NFHS1_5June2024.dta", clear

// 1 IS MALE//
mean widow [aw=wt] if (hv104==1 & age_grp==1), over(newstate)
mean widow [aw=wt] if (hv104==1 & age_grp==2), over(newstate)
mean widow [aw=wt] if (hv104==1 & age_grp==3), over(newstate)
mean widow [aw=wt] if (hv104==1 & age_grp==4), over(newstate)
// 2 IS FEMALE//
mean widow [aw=wt] if (hv104==2 & age_grp==1), over(newstate)
mean widow [aw=wt] if (hv104==2 & age_grp==2), over(newstate)
mean widow [aw=wt] if (hv104==2 & age_grp==3), over(newstate)
mean widow [aw=wt] if (hv104==2 & age_grp==4), over(newstate)


***Table4***
/// Headcount (N) of widowhood among ever-married men and women in 2021, and 1993, across states and union territories of India///

///NFHS5///
use "/Users/IITMandi/Downloads/Widowhood_NFHS5_5June2024.dta", clear
ta hv024 widow[iw=popwt] if (hv104==1 & age_grp==1)
ta hv024 widow[iw=popwt] if (hv104==1 & age_grp==2)
ta hv024 widow[iw=popwt] if (hv104==1 & age_grp==3)
ta hv024 widow[iw=popwt] if (hv104==1 & age_grp==4)
ta hv024 widow[iw=popwt] if (hv104==2 & age_grp==1)
ta hv024 widow[iw=popwt] if (hv104==2 & age_grp==2)
ta hv024 widow[iw=popwt] if (hv104==2 & age_grp==3)
ta hv024 widow[iw=popwt] if (hv104==2 & age_grp==4)

///NFHS1///
use "/Users/IITMandi/Downloads/Widowhood_NFHS1_5June2024.dta", clear
ta newstate widow[iw=popwt] if (hv104==1 & age_grp==1)
ta newstate widow[iw=popwt] if (hv104==1 & age_grp==2)
ta newstate widow[iw=popwt] if (hv104==1 & age_grp==3)
ta newstate widow[iw=popwt] if (hv104==1 & age_grp==4)
ta newstate widow[iw=popwt] if (hv104==2 & age_grp==1)
ta newstate widow[iw=popwt] if (hv104==2 & age_grp==2)
ta newstate widow[iw=popwt] if (hv104==2 & age_grp==3)
ta newstate widow[iw=popwt] if (hv104==2 & age_grp==4)

***Supplementary Table1***
///Sample size (n) of ever-married men and women aged 75years or above and unweighted percentage (%) of widows among them in 2021, 2016, 2006, 1999, and 1993 across states and union territories India///

///NFHS5///
use "/Users/IITMandi/Downloads/Widowhood_NFHS5_5June2024.dta", clear
ta hv024 widow if (hv104==1 & age_grp==4)
ta hv024 widow if (hv104==1 & age_grp==4), r nof
ta hv024 widow if (hv104==2 & age_grp==4)
ta hv024 widow if (hv104==2 & age_grp==4), r nof

///NFHS4///
use "/Users/IITMandi/Downloads/Widowhood_NFHS4_5June_2024.dta", clear
ta hv024 widow if (hv104==1 & age_grp==4)
ta hv024 widow if (hv104==1 & age_grp==4), r nof
ta hv024 widow if (hv104==2 & age_grp==4)
```

```
ta hv024 widow if (hv104==2 & age_grp==4), r nof

///NFHS3///
use "/Users/IITMandi/Downloads/Widowhood_NFHS3_5June2024.dta", clear
ta hv024 widow if (hv104==1 & age_grp==4)
ta hv024 widow if (hv104==1 & age_grp==4), r nof
ta hv024 widow if (hv104==2 & age_grp==4)
ta hv024 widow if (hv104==2 & age_grp==4), r nof

///NFHS2///
use "/Users/IITMandi/Downloads/Widowhood_NFHS2_5June2024.dta", clear
ta newstate widow if (hv104==1 & age_grp==4)
ta newstate widow if (hv104==1 & age_grp==4), r nof
ta newstate widow if (hv104==2 & age_grp==4)
ta newstate widow if (hv104==2 & age_grp==4), r nof

///NFHS1///
use "/Users/IITMandi/Downloads/Widowhood_NFHS1_5June2024.dta", clear
ta newstate widow if (hv104==1 & age_grp==4)
ta newstate widow if (hv104==1 & age_grp==4), r nof
ta newstate widow if (hv104==2 & age_grp==4)
ta newstate widow if (hv104==2 & age_grp==4), r nof

**Supplementary Table2***
///Sample size (n) of ever-married men and women aged 65 TO 74 years and unweighted percentage (%) of widows among them in 2021, 2016, 2006, 1999, and 1993 across states and union territories India///

///NFHS5///
use "/Users/IITMandi/Downloads/Widowhood_NFHS5_5June2024.dta", clear
ta hv024 widow if (hv104==1 & age_grp==3)
ta hv024 widow if (hv104==1 & age_grp==3), r nof
ta hv024 widow if (hv104==2 & age_grp==3)
ta hv024 widow if (hv104==2 & age_grp==3), r nof

///NFHS4///
use "/Users/IITMandi/Downloads/Widowhood_NFHS4_5June_2024.dta", clear
ta hv024 widow if (hv104==1 & age_grp==3)
ta hv024 widow if (hv104==1 & age_grp==3), r nof
ta hv024 widow if (hv104==2 & age_grp==3)
ta hv024 widow if (hv104==2 & age_grp==3), r nof

///NFHS3///
use "/Users/IITMandi/Downloads/Widowhood_NFHS3_5June2024.dta", clear
ta hv024 widow if (hv104==1 & age_grp==3)
ta hv024 widow if (hv104==1 & age_grp==3), r nof
ta hv024 widow if (hv104==2 & age_grp==3)
ta hv024 widow if (hv104==2 & age_grp==3), r nof

///NFHS2///
use "/Users/IITMandi/Downloads/Widowhood_NFHS2_5June2024.dta", clear
ta newstate widow if (hv104==1 & age_grp==3)
ta newstate widow if (hv104==1 & age_grp==3), r nof
ta newstate widow if (hv104==2 & age_grp==3)
ta newstate widow if (hv104==2 & age_grp==3), r nof

///NFHS1///
use "/Users/IITMandi/Downloads/Widowhood_NFHS1_5June2024.dta", clear
ta newstate widow if (hv104==1 & age_grp==3)
ta newstate widow if (hv104==1 & age_grp==3), r nof
ta newstate widow if (hv104==2 & age_grp==3)
ta newstate widow if (hv104==2 & age_grp==3), r nof

**Supplementary Table3***
///Sample size (n) of ever-married men and women aged 45 TO 64 years and unweighted percentage (%) of widows among them in 2021, 2016, 2006, 1999, and 1993 across states and union territories India///

///NFHS5///
use "/Users/IITMandi/Downloads/Widowhood_NFHS5_5June2024.dta", clear
ta hv024 widow if (hv104==1 & age_grp==2)
ta hv024 widow if (hv104==1 & age_grp==2), r nof
```

```
ta hv024 widow if (hv104==2 & age_grp==2)
ta hv024 widow if (hv104==2 & age_grp==2), r nof

///NFHS4///
use "/Users/IITMandi/Downloads/Widowhood_NFHS4_5June_2024.dta", clear
ta hv024 widow if (hv104==1 & age_grp==2)
ta hv024 widow if (hv104==1 & age_grp==2), r nof
ta hv024 widow if (hv104==2 & age_grp==2)
ta hv024 widow if (hv104==2 & age_grp==2), r nof

///NFHS3///
use "/Users/IITMandi/Downloads/Widowhood_NFHS3_5June2024.dta", clear
ta hv024 widow if (hv104==1 & age_grp==2)
ta hv024 widow if (hv104==1 & age_grp==2), r nof
ta hv024 widow if (hv104==2 & age_grp==2)
ta hv024 widow if (hv104==2 & age_grp==2), r nof

///NFHS2///
use "/Users/IITMandi/Downloads/Widowhood_NFHS2_5June2024.dta", clear
ta newstate widow if (hv104==1 & age_grp==2)
ta newstate widow if (hv104==1 & age_grp==2), r nof
ta newstate widow if (hv104==2 & age_grp==2)
ta newstate widow if (hv104==2 & age_grp==2), r nof

///NFHS1///
use "/Users/IITMandi/Downloads/Widowhood_NFHS1_5June2024.dta", clear
ta newstate widow if (hv104==1 & age_grp==2)
ta newstate widow if (hv104==1 & age_grp==2), r nof
ta newstate widow if (hv104==2 & age_grp==2)
ta newstate widow if (hv104==2 & age_grp==2), r nof

***Supplementary Table4***
///Sample size (n) of ever-married men and women aged less than 45 years and unweighted percentage (%) of
widows among them in 2021, 2016, 2006, 1999, and 1993 across states and union territories India///

///NFHS5///
use "/Users/IITMandi/Downloads/Widowhood_NFHS5_5June2024.dta", clear
ta hv024 widow if (hv104==1 & age_grp==1)
ta hv024 widow if (hv104==1 & age_grp==1), r nof
ta hv024 widow if (hv104==2 & age_grp==1)
ta hv024 widow if (hv104==2 & age_grp==1), r nof

///NFHS4///
use "/Users/IITMandi/Downloads/Widowhood_NFHS4_5June_2024.dta", clear
ta hv024 widow if (hv104==1 & age_grp==1)
ta hv024 widow if (hv104==1 & age_grp==1), r nof
ta hv024 widow if (hv104==2 & age_grp==1)
ta hv024 widow if (hv104==2 & age_grp==1), r nof

///NFHS3///
use "/Users/IITMandi/Downloads/Widowhood_NFHS3_5June2024.dta", clear
ta hv024 widow if (hv104==1 & age_grp==1)
ta hv024 widow if (hv104==1 & age_grp==1), r nof
ta hv024 widow if (hv104==2 & age_grp==1)
ta hv024 widow if (hv104==2 & age_grp==1), r nof

///NFHS2///
use "/Users/IITMandi/Downloads/Widowhood_NFHS2_5June2024.dta", clear
ta newstate widow if (hv104==1 & age_grp==1)
ta newstate widow if (hv104==1 & age_grp==1), r nof
ta newstate widow if (hv104==2 & age_grp==1)
ta newstate widow if (hv104==2 & age_grp==1), r nof

///NFHS1///
use "/Users/IITMandi/Downloads/Widowhood_NFHS1_5June2024.dta", clear
ta newstate widow if (hv104==1 & age_grp==1)
ta newstate widow if (hv104==1 & age_grp==1), r nof
ta newstate widow if (hv104==2 & age_grp==1)
ta newstate widow if (hv104==2 & age_grp==1), r nof
```

```
***Supplementary Table5***
///Prevalence (and 95% CI) of widowhood among ever-married men and women aged 75 or above, 65 to 74, 45 to 64
and less than 45 in 2021, and 1993 across states and union territories India///

///NFHS5///
use "/Users/IITMandi/Downloads/Widowhood_NFHS5_5June2024.dta", clear
// 1 IS MALE//
mean widow [aw=wt] if (hv104==1 & age_grp==4), over(hv024)
mean widow [aw=wt] if (hv104==1 & age_grp==3), over(hv024)
mean widow [aw=wt] if (hv104==1 & age_grp==2), over(hv024)
mean widow [aw=wt] if (hv104==1 & age_grp==1), over(hv024)
// 2 IS FEMALE//
mean widow [aw=wt] if (hv104==2 & age_grp==4), over(hv024)
mean widow [aw=wt] if (hv104==2 & age_grp==3), over(hv024)
mean widow [aw=wt] if (hv104==2 & age_grp==2), over(hv024)
mean widow [aw=wt] if (hv104==2 & age_grp==1), over(hv024)

///NFHS1//
use "/Users/IITMandi/Downloads/Widowhood_NFHS1_5June2024.dta", clear
// 1 IS MALE//
mean widow [aw=wt] if (hv104==1 & age_grp==4), over(newstate)
mean widow [aw=wt] if (hv104==1 & age_grp==3), over(newstate)
mean widow [aw=wt] if (hv104==1 & age_grp==2), over(newstate)
mean widow [aw=wt] if (hv104==1 & age_grp==1), over(newstate)

// 2 IS FEMALE//
mean widow [aw=wt] if (hv104==2 & age_grp==4), over(newstate)
mean widow [aw=wt] if (hv104==2 & age_grp==3), over(newstate)
mean widow [aw=wt] if (hv104==2 & age_grp==2), over(newstate)
mean widow [aw=wt] if (hv104==2 & age_grp==1), over(newstate)


***Supplementary Table 6***
///Headcount (N) of widowhood among ever-married men and women by age groups in 2016, 2006,and 1999 across
states and union territories India ///

///NFHS4///
use "/Users/IITMandi/Downloads/Widowhood_NFHS4_5June_2024.dta", clear
ta hv024 widow[iw=popwt] if (hv104==1 & age_grp==1)
ta hv024 widow[iw=popwt] if (hv104==1 & age_grp==2)
ta hv024 widow[iw=popwt] if (hv104==1 & age_grp==3)
ta hv024 widow[iw=popwt] if (hv104==1 & age_grp==4)
ta hv024 widow[iw=popwt] if (hv104==2 & age_grp==1)
ta hv024 widow[iw=popwt] if (hv104==2 & age_grp==2)
ta hv024 widow[iw=popwt] if (hv104==2 & age_grp==3)
ta hv024 widow[iw=popwt] if (hv104==2 & age_grp==4)

///NFHS3///
use "/Users/IITMandi/Downloads/Widowhood_NFHS3_5June2024.dta", clear
ta hv024 widow[iw=popwt] if (hv104==1 & age_grp==1)
ta hv024 widow[iw=popwt] if (hv104==1 & age_grp==2)
ta hv024 widow[iw=popwt] if (hv104==1 & age_grp==3)
ta hv024 widow[iw=popwt] if (hv104==1 & age_grp==4)
ta hv024 widow[iw=popwt] if (hv104==2 & age_grp==1)
ta hv024 widow[iw=popwt] if (hv104==2 & age_grp==2)
ta hv024 widow[iw=popwt] if (hv104==2 & age_grp==3)
ta hv024 widow[iw=popwt] if (hv104==2 & age_grp==4)

///NFHS2///
use "/Users/IITMandi/Downloads/Widowhood_NFHS2_5June2024.dta", clear
ta newstate widow[iw=popwt] if (hv104==1 & age_grp==1)
ta newstate widow[iw=popwt] if (hv104==1 & age_grp==2)
ta newstate widow[iw=popwt] if (hv104==1 & age_grp==3)
ta newstate widow[iw=popwt] if (hv104==1 & age_grp==4)
ta newstate widow[iw=popwt] if (hv104==2 & age_grp==1)
ta newstate widow[iw=popwt] if (hv104==2 & age_grp==2)
ta newstate widow[iw=popwt] if (hv104==2 & age_grp==3)
ta newstate widow[iw=popwt] if (hv104==2 & age_grp==4)

***Supplementary Table9***
```

```
// Sensitivity analysis of widohood in the ages 45 to 64 and less than 45, in the year 2021, 2016, 2006.
//NFHS5//

//IR_File//
use "/Users/IITMandi/Downloads/IAIR7EDT/IAIR7EFL.DTA", clear
tab1 s301 v501, m
tab1 s301 v501, nol m
recode s301 (0=0 "never_married") (1=1 "currenlt_married") (2=0) (3=3 "widowed") (4=4 "divorced") (5=5
"no_longer_living_together") (6=5), ge(marital_status)
tab1 v501 marital_status
** checking the difference in reporting **
ge x=.
replace x=1 if marital_status!=v501
rename (v001 v002 v003) (hv001 hv002 hvidx)
keep hv001 hv002 hvidx v501
save "/Users/IITMandi/Downloads/IR_SA.dta"

//MR_File//
use "/Volumes/AVNISH/NFHS_Data/NFHS5/IAMR7EDT/IAMR7EFL.DTA"
tab1 sm213 mv501, m
tab1 sm213 mv501, nol m
recode sm213 (0=0 "never_married") (1=1 "currenlt_married") (2=0) (3=3 "widowed") (4=4 "divorced") (5=5
"no_longer_living_together") (6=5), ge(marital_status)
tab1 mv501 marital_status
** checking the difference in reporting  **
ge x=.
replace x=1 if marital_status!=mv501
rename (mv001 mv002 mv003 mv501) (hv001 hv002 hvidx v501)
keep hv001 hv002 hvidx v501
save "/Users/IITMandi/Downloads/MR_SA.dta"

// adding IR+MR File //
use "/Users/IITMandi/Downloads/IR_SA.dta", clear
merge 1:1 hv001 hv002 hvidx using "/Users/IITMandi/Downloads/MR_SA.dta"
drop _merge
save "/Users/IITMandi/Downloads/IR+MR_SA.dta"

// adding PR and (IR+MR)//
use "/Volumes/AVNISH/NFHS_Data/NFHS5/IAPR7EDT/IAPR7EFL.DTA"
merge 1:1 hv001 hv002 hvidx using "/Users/IITMandi/Downloads/IR+MR_SA.dta"
save "/Users/IITMandi/Downloads/IR+MR+PR_SA.dta"

use"/Users/IITMandi/Downloads/IR+MR+PR_SA.dta", clear
clonevar mar=hv115
replace mar=v501 if _merge==3
tab1 hv115 hv116, m
tab1 hv115 hv116, nol m
drop if (hv116==0 | hv116==. | hv116==.a)
ge wt= hv005/1000000
recode mar (3=1 "widowed") (.=.) (else=0 "not widowed"), ge(widow1)
recode hv115 (3=1 "widowed") (.=.) (else=0 "not widowed"), ge(widow)
recode hv105 (13/44=1 "less than 45") (45/64=2 "45 t0 64") (65/74=3 "65 to 74") (75/max=4 "75+") (else=.),
ge(age_grp)
ge v=.
replace v=1 if age_grp==1 & hv104==1
replace v=2 if age_grp==2 & hv104==1
replace v=3 if age_grp==3 & hv104==1
replace v=4 if age_grp==4 & hv104==1
replace v=5 if age_grp==1 & hv104==2
replace v=6 if age_grp==2 & hv104==2
replace v=7 if age_grp==3 & hv104==2
replace v=8 if age_grp==4 & hv104==2
ta v
ge pop=.
replace pop= 370384000 if v==1
replace pop= 126986000 if v==2
replace pop= 29369000 if v==3
replace pop= 14906000 if v==4
replace pop= 345933000 if v==5
replace pop= 124375000 if v==6
```

```
replace pop= 31487000 if v==7
replace pop= 17264000 if v==8
ta pop
ta v
egen sumwt = sum(wt), by(v)
ge popwt= (wt/sumwt)*pop

mean widow [aw=wt] if hv104==1, over (age_grp)
mean widow1 [aw=wt] if hv104==1, over (age_grp)
mean widow [aw=wt] if hv104==2, over (age_grp)
mean widow1 [aw=wt] if hv104==2, over (age_grp)

///Headcoun (N)///
ta age_grp widow[iw=popwt] if hv104==1
ta age_grp widow1[iw=popwt] if hv104==1
ta age_grp widow[iw=popwt] if hv104==2
ta age_grp widow1[iw=popwt] if hv104==2


/// NFHS4///

//IR_File//
use "/Volumes/AVNISH/NFHS_Data/NFHS4/IAIR74DT/IAIR74FL.DTA", clear
tab1 s301 v501, m
tab1 s301 v501, nol m
recode s301 (0=0 "never_married") (1=1 "currenlt_married") (2=0) (3=3 "widowed") (4=4 "divorced") (5=5
"no_longer_living_together") (6=5), ge(marital_status)
tab1 v501 marital_status
** checking the difference in reporting **
ge x=.
replace x=1 if marital_status!=v501
rename (v001 v002 v003) (hv001 hv002 hvidx)
keep hv001 hv002 hvidx v501
save "/Volumes/AVNISH/NFHS_Data/NFHS4/IR_SA_NFHS4.dta"

//MR_File//
use "/Volumes/AVNISH/NFHS_Data/NFHS4/IAMR74DT/IAMR74FL.DTA", clear
tab1 sm213 mv501, m
tab1 sm213 mv501, nol m
recode sm213 (0=0 "never_married") (1=1 "currenlt_married") (2=0) (3=3 "widowed") (4=4 "divorced") (5=5
"no_longer_living_together") (6=5), ge(marital_status)
tab1 mv501 marital_status
** checking the difference in reporting  **
ge x=.
replace x=1 if marital_status!=mv501
rename (mv001 mv002 mv003 mv501) (hv001 hv002 hvidx v501)
keep hv001 hv002 hvidx v501
save "/Volumes/AVNISH/NFHS_Data/NFHS4/MR_SA_NFHS4.dta"

// adding IR+MR File //
use "/Volumes/AVNISH/NFHS_Data/NFHS4/IR_SA_NFHS4.dta", clear
merge 1:1 hv001 hv002 hvidx using "/Volumes/AVNISH/NFHS_Data/NFHS4/MR_SA_NFHS4.dta"
drop _merge
save "/Volumes/AVNISH/NFHS_Data/NFHS4/IR+MR_SA_NFHS4.dta"

// adding PR and (IR+MR)//
use "/Volumes/AVNISH/NFHS_Data/NFHS4/IAPR74DT/IAPR74FL.DTA", clear
merge 1:1 hv001 hv002 hvidx using "/Volumes/AVNISH/NFHS_Data/NFHS4/IR+MR_SA_NFHS4.dta"
save "/Volumes/AVNISH/NFHS_Data/NFHS4/IR+MR+PR_SA_NFHS4.dta"


use "/Volumes/AVNISH/NFHS_Data/NFHS4/IR+MR+PR_SA_NFHS4.dta", clear
replace hv024=37 if shdistri==3 | shdistri==4
label define hv024 37 "ladakh", add
replace hv024=38 if hv024==8 | hv024==9
label define hv024 38 "dadra & nagar haveli and daman & diu", add
label define hv024 8 "", modify
label define hv024 9 "", modify

clonevar mar=hv115
```

```
replace mar=v501 if _merge==3
tab1 hv115 hv116, m
tab1 hv115 hv116, nol m
drop if (hv116==0 | hv116==.)

ge wt= hv005/1000000
recode mar (3=1 "widowed") (.=.) (else=0 "not widowed"), ge(widow1)
recode hv115 (3=1 "widowed") (.=.) (else=0 "not widowed"), ge(widow)
recode hv105 (13/44=1 "less than 45") (45/64=2 "45 t0 64") (65/74=3 "65 to 74") (75/max=4 "75+") (else=.),
ge(age_grp)

ge v=.
replace v=1 if age_grp==1 & hv104==1
replace v=2 if age_grp==2 & hv104==1
replace v=3 if age_grp==3 & hv104==1
replace v=4 if age_grp==4 & hv104==1
replace v=5 if age_grp==1 & hv104==2
replace v=6 if age_grp==2 & hv104==2
replace v=7 if age_grp==3 & hv104==2
replace v=8 if age_grp==4 & hv104==2
ta v
ge pop=.
replace pop= 351778000 if v==1
replace pop= 110876000 if v==2
replace pop= 26200000 if v==3
replace pop= 12199000 if v==4
replace pop= 330207000 if v==5
replace pop= 106718000 if v==6
replace pop= 27837000 if v==7
replace pop= 13528000 if v==8
ta pop
ta v
egen sumwt = sum(wt), by(v)
ge popwt= (wt/sumwt)*pop

mean widow [aw=wt] if hv104==1, over (age_grp)
mean widow1 [aw=wt] if hv104==1, over (age_grp)
mean widow [aw=wt] if hv104==2, over (age_grp)
mean widow1 [aw=wt] if hv104==2, over (age_grp)

///Headcoun (N)///
ta age_grp widow[iw=popwt] if hv104==1
ta age_grp widow1[iw=popwt] if hv104==1
ta age_grp widow[iw=popwt] if hv104==2
ta age_grp widow1[iw=popwt] if hv104==2

///NFHS3//

//IR_File//
use "/Volumes/AVNISH/NFHS_Data/NFHS3/IAIR52DT/IAIR52FL.dta", clear
tab1 s301 v501, m
tab1 s301 v501, nol m
recode s301 (0=0 "never_married") (1=1 "currenlt_married") (2=0) (3=3 "widowed") (4=4 "divorced") (5=5
"no_longer_living_together") (6=5), ge(marital_status)
tab1 v501 marital_status
** checking the difference in reporting **
ge x=.
replace x=1 if marital_status!=v501
rename (v001 v002 v003) (hv001 hv002 hvidx)
keep hv001 hv002 hvidx v501
save "/Volumes/AVNISH/NFHS_Data/NFHS3/IR_SA_NFHS3.dta"

//MR_File//
use "/Volumes/AVNISH/NFHS_Data/NFHS3/IAMR52DT/IAMR52FL.dta", clear
tab1 sm401 mv501, m
tab1 sm401 mv501, m
recode sm401 (0=0 "never_married") (1=1 "currenlt_married") (2=0) (3=3 "widowed") (4=4 "divorced") (5=5
"no_longer_living_together") (6=5), ge(marital_status)
tab1 mv501 marital_status
** checking the difference in reporting  **
```

```
ge x=.
replace x=1 if marital_status!=mv501
rename (mv001 mv002 mv003 mv501) (hv001 hv002 hvidx v501)
keep hv001 hv002 hvidx v501
save"/Volumes/AVNISH/NFHS_Data/NFHS3/MR_SA_NFHS3.dta"

// adding IR+MR File //
use "/Volumes/AVNISH/NFHS_Data/NFHS3/IR_SA_NFHS3.dta", clear
merge 1:1 hv001 hv002 hvidx using "/Volumes/AVNISH/NFHS_Data/NFHS3/MR_SA_NFHS3.dta"
drop _merge
save "/Volumes/AVNISH/NFHS_Data/NFHS3/IR+MR_SA_NFHS3.dta"

// adding PR and (IR+MR)//
use "/Volumes/AVNISH/NFHS_Data/NFHS3/IAPR52DT/IAPR52FL.DTA", clear
merge 1:1 hv001 hv002 hvidx using "/Volumes/AVNISH/NFHS_Data/NFHS3/IR+MR_SA_NFHS3.dta"
save "/Volumes/AVNISH/NFHS_Data/NFHS3/IR+MR+PR_SA_NFHS3.dta"

use "/Volumes/AVNISH/NFHS_Data/NFHS3/IR+MR+PR_SA_NFHS3.dta", clear
tab1 hv115 hv116, m
tab1 hv115 hv116, nol m
drop if (hv116==0 | hv116==.)
tab hv115, m
tab hv105,m
clonevar mar=hv115
replace mar=v501 if _merge==3
tab1 hv115 hv116, m
tab1 hv115 hv116, nol m
drop if (hv116==0 | hv116==.)
ge wt= hv005/1000000
recode mar (3=1 "widowed") (.=.) (else=0 "not widowed"), ge(widow1)
recode hv115 (3=1 "widowed") (.=.) (else=0 "not widowed"), ge(widow)
drop if (hv105==10 | hv105==11 | hv105==12 | hv105==98 | hv105==.)
recode hv105 (13/44=1 "less than 45") (45/64=2 "45 t0 64") (65/74=3 "65 to 74") (75/max=4 "75+") (else=.),
ge(age_grp)

ge v=.
replace v=1 if age_grp==1 & hv104==1
replace v=2 if age_grp==2 & hv104==1
replace v=3 if age_grp==3 & hv104==1
replace v=4 if age_grp==4 & hv104==1
replace v=5 if age_grp==1 & hv104==2
replace v=6 if age_grp==2 & hv104==2
replace v=7 if age_grp==3 & hv104==2
replace v=8 if age_grp==4 & hv104==2
ta v
ge pop=.
replace pop= 302508000 if v==1
replace pop= 85166000 if v==2
replace pop= 18878000 if v==3
replace pop= 8144000 if v==4
replace pop= 281723000 if v==5
replace pop= 78550000 if v==6
replace pop= 20488000 if v==7
replace pop= 8628000 if v==8
ta pop
ta v
egen sumwt = sum(wt), by(v)
ge popwt= (wt/sumwt)*pop

mean widow [aw=wt] if hv104==1, over (age_grp)
mean widow1 [aw=wt] if hv104==1, over (age_grp)
mean widow [aw=wt] if hv104==2, over (age_grp)
mean widow1 [aw=wt] if hv104==2, over (age_grp)

///Headcoun (N)///
ta age_grp widow[iw=popwt] if hv104==1
ta age_grp widow1[iw=popwt] if hv104==1
ta age_grp widow[iw=popwt] if hv104==2
ta age_grp widow1[iw=popwt] if hv104==2
```

```
***Supplementary Table10**
/// comparison of widowhood reported by the head of the household and the self-reported widowhood in the ages 
15 to 44, 45 to 49 and 50 to 54 in 2021, 2016, 2006, 1999, and 1993.

//NFHS_5 //
// IR file //

use "/Volumes/Avnish256/NFHS_Data/NFHS_5/IAIR7EDT-2/IAIR7EFL.DTA", clear
tab v501, m
tab v501, nol m
tab v012, m
ge wt= v005/1000000
drop if v501==0
recode v501 (3=1 "widowed") (else=0 "not_widowed"), ge (widow_ir)
recode v012 (15/44=1 "15-44") (45/49=2 "45-49"), ge (age_grp_ir)
ta age_grp_ir widow_ir /// sample size //
ta age_grp_ir widow_ir [aw=wt], r nof // weighted widowhood //

// MR File //
use "/Volumes/Avnish256/NFHS_Data/NFHS_5/IAMR7EDT/IAMR7EFL.DTA", clear
tab mv501, m
tab mv501, nol m
tab mv012, m
ge wt= mv005/1000000
drop if mv501==0
recode mv501 (3=1 "widowed") (else=0 "not_widowed"), ge (widow_mr)
recode mv012 (15/44=1 "15-44") (45/49=2 "45-49") (50/54=3 "50-54"), ge (age_grp_mr)
ta age_grp_mr widow_mr /// sample size //
ta age_grp_mr widow_mr [aw=wt], r nof // weighted widowhood //

// PR File //

use "/Volumes/Avnish256/NFHS_Data/NFHS_5/IAPR7EDT/IAPR7EFL.DTA", clear
tab hv115, m
tab hv115, nol m
drop if (hv115==0 | hv115==8 | hv115==.)
tab hv105, m
ge wt= hv005/1000000
recode hv105 (15/44=1 "15-44") (45/49=2 "45_49") (50/54=3 "50-54"), ge (age_grp_pr)
recode hv115 (3=1 "widowed") (else=0 "not_widowed"), ge (widow_pr)
ta age_grp_pr widow_pr if hv104==1 // sample size male //
ta age_grp_pr widow_pr [aw=wt] if hv104==1, r nof // weighted widowhood male//
ta age_grp_pr widow_pr if hv104==2 // sample size female //
ta age_grp_pr widow_pr [aw=wt] if hv104==2, r nof // weighted widowhood female//

***
// NFHS_4 //

//IR File //
use "/Volumes/Avnish256/NFHS_Data/NFHS_4/IAIR74DT/IAIR74FL.DTA", clear
tab v501, m
tab v501, nol m
tab v012, m
ge wt= v005/1000000
drop if v501==0
recode v501 (3=1 "widowed") (else=0 "not_widowed"), ge (widow_ir)
recode v012 (15/44=1 "15-44") (45/49=2 "45-49"), ge (age_grp_ir)
ta age_grp_ir widow_ir /// sample size //
ta age_grp_ir widow_ir [aw=wt], r nof // weighted widowhood //


//MR File //
use "/Volumes/Avnish256/NFHS_Data/NFHS_4/IAMR74DT/IAMR74FL.DTA", clear
tab mv501, m
tab mv501, nol m
tab mv012, m
ge wt= mv005/1000000
drop if mv501==0
recode mv501 (3=1 "widowed") (else=0 "not_widowed"), ge (widow_mr)
recode mv012 (15/44=1 "15-44") (45/49=2 "45-49") (50/54=3 "50-54"), ge (age_grp_mr)
```

```
ta age_grp_mr widow_mr /// sample size //
ta age_grp_mr widow_mr [aw=wt], r nof // weighted widowhood //

// PR file //
use "/Volumes/Avnish256/NFHS_Data/NFHS_4/IAPR74DT/IAPR74FL.DTA", clear
tab hv115, m
tab hv115, nol m
drop if (hv115==0 | hv115==8 | hv115==.)
tab hv105, m
ge wt= hv005/1000000
recode hv105 (15/44=1 "15-44") (45/49=2 "45_49") (50/54=3 "50-54"), ge (age_grp_pr)
recode hv115 (3=1 "widowed") (else=0 "not_widowed"), ge (widow_pr)
ta age_grp_pr widow_pr if hv104==1 // sample size male //
ta age_grp_pr widow_pr [aw=wt] if hv104==1, r nof // weighted widowhood male//
ta age_grp_pr widow_pr if hv104==2 // sample size female //
ta age_grp_pr widow_pr [aw=wt] if hv104==2, r nof // weighted widowhood female//

***
// NFHS3//

// IR File //
use "/Volumes/Avnish256/NFHS_Data/NFHS_3/IAIR52DT/IAIR52FL.dta", clear
tab v501, m
tab v501, nol m
tab v012, m
ge wt= v005/1000000
drop if v501==0
recode v501 (3=1 "widowed") (else=0 "not_widowed"), ge (widow_ir)
recode v012 (15/44=1 "15-44") (45/49=2 "45-49"), ge (age_grp_ir)
ta age_grp_ir widow_ir /// sample size //
ta age_grp_ir widow_ir [aw=wt], r nof // weighted widowhood //

//MR File //
use "/Volumes/Avnish256/NFHS_Data/NFHS_3/IAMR52DT/IAMR52FL.dta", clear
tab mv501, m
tab mv501, nol m
tab mv012, m
ge wt= mv005/1000000
drop if mv501==0
recode mv501 (3=1 "widowed") (else=0 "not_widowed"), ge (widow_mr)
recode mv012 (15/44=1 "15-44") (45/49=2 "45-49") (50/54=3 "50-54"), ge (age_grp_mr)
ta age_grp_mr widow_mr /// sample size //
ta age_grp_mr widow_mr [aw=wt], r nof // weighted widowhood //

// PR file//
use "/Volumes/Avnish256/NFHS_Data/NFHS_3/IAPR52DT/IAPR52FL.DTA", clear
tab hv115, m
tab hv115, nol m
drop if (hv115==0 | hv115==8 | hv115==.)
tab hv105, m
drop if (hv105==98 | hv105 ==.)
ge wt= hv005/1000000
drop if (hv105==10 | hv105==11 | hv105==12 | hv105==98 | hv105==.)
recode hv105 (15/44=1 "15-44") (45/49=2 "45_49") (50/54=3 "50-54"), ge (age_grp_pr)
recode hv115 (3=1 "widowed") (else=0 "not_widowed"), ge (widow_pr)
ta age_grp_pr widow_pr if hv104==1 // sample size male //
ta age_grp_pr widow_pr [aw=wt] if hv104==1, r nof // weighted widowhood male//
ta age_grp_pr widow_pr if hv104==2 // sample size female //
ta age_grp_pr widow_pr [aw=wt] if hv104==2, r nof // weighted widowhood female//

// NFHS2//

//IR File //

use "/Volumes/Avnish256/NFHS_Data/NFHS_2/IAIR42DT/IAIR42FL.DTA", clear
tab v501, m
tab v501, nol m
tab v012, m
ge wt= v005/1000000
drop if v501==0
```

```
recode v501 (3=1 "widowed") (else=0 "not_widowed"), ge (widow_ir)
recode v012 (15/44=1 "15-44") (45/49=2 "45-49"), ge (age_grp_ir)
ta age_grp_ir widow_ir /// sample size //
ta age_grp_ir widow_ir [aw=wt], r nof // weighted widowhood //

// no MR file//

//PR file//
use "/Volumes/Avnish256/NFHS_Data/NFHS_2/IAPR42DT/IAPR42FL.DTA", clear
tab hv115, m
tab hv115, nol m
drop if (hv115==0| hv115==.)
tab hv105, m
drop if hv105 ==.
ge wt= hv005/1000000
drop if (hv105==6 | hv105==7 | hv105==8 | hv105==9 | hv105==10 |hv105==11 |hv105==12 |hv105==.)
recode hv105 (15/44=1 "15-44") (45/49=2 "45_49") (50/54=3 "50-54"), ge (age_grp_pr)
recode hv115 (3=1 "widowed") (else=0 "not_widowed"), ge (widow_pr)
ta age_grp_pr widow_pr if hv104==1 // sample size male //
ta age_grp_pr widow_pr [aw=wt] if hv104==1, r nof // weighted widowhood male//
ta age_grp_pr widow_pr if hv104==2 // sample size female //
ta age_grp_pr widow_pr [aw=wt] if hv104==2, r nof // weighted widowhood female//

// NFHS1//

//IR file//
use "/Volumes/Avnish256/NFHS_Data/NFHS_1/IAIR23DT/IAIR23FL.DTA", clear
tab v501, m
tab v501, nol m
tab v012, m
ge wt= v005/1000000
recode v501 (3=1 "widowed") (else=0 "not_widowed"), ge (widow_ir)
recode v012 (15/44=1 "15-44") (45/49=2 "45-49"), ge (age_grp_ir)
ta age_grp_ir widow_ir /// sample size //
ta age_grp_ir widow_ir [aw=wt], r nof // weighted widowhood //

// no MR file//

// PR file //
// PR file //
use "/Volumes/Avnish256/NFHS_Data/NFHS_1/IAPR23DT/IAPR23FL.DTA", clear
tab hv115, m
tab hv115, nol m
drop if (hv115==0| hv115==6| hv115==9)
tab hv105, m
tab hv104, m
drop if hv104==9
ge wt= hv005/1000000
drop if (hv105==6 | hv105==7 | hv105==8 | hv105==9 | hv105==10 |hv105==11 |hv105==12 |hv105==.)
recode hv105 (15/44=1 "15-44") (45/49=2 "45_49") (50/54=3 "50-54"), ge (age_grp_pr)
recode hv115 (3=1 "widowed") (else=0 "not_widowed"), ge (widow_pr)
ta age_grp_pr widow_pr if hv104==1 // sample size male //
ta age_grp_pr widow_pr [aw=wt] if hv104==1, r nof // weighted widowhood male//
ta age_grp_pr widow_pr if hv104==2 // sample size female //
ta age_grp_pr widow_pr [aw=wt] if hv104==2, r nof // weighted widowhood female//

***Supplemetary Table11***
///Prevalence (and 95% CI) of widowhood among ever-married men and women aged 75 or above in 2016,2006, and 1999 across states and union territories India///

///NFHS4///
use "/Users/IITMandi/Downloads/Widowhood_NFHS4_5June_2024.dta", clear
// 1 IS MALE //
mean widow [aw=wt] if (hv104==1 & age_grp==4), over(hv024)
// 2 IS FEMALE //
mean widow [aw=wt] if (hv104==2 & age_grp==4), over(hv024)

///NFHS3///
use "/Users/IITMandi/Downloads/Widowhood_NFHS3_5June2024.dta", clear
// 1 IS MALE //
```

```
mean widow [aw=wt] if (hv104==1 & age_grp==4), over(hv024)
// 2 IS FEMALE //
mean widow [aw=wt] if (hv104==2 & age_grp==4), over(hv024)

///NFHS2///
use "/Users/IITMandi/Downloads/Widowhood_NFHS2_5June2024.dta", clear
// 1 IS MALE//
mean widow [aw=wt] if (hv104==1 & age_grp==4), over(newstate)
// 2 IS FEMALE//
mean widow [aw=wt] if (hv104==2 & age_grp==4), over(newstate)

***Supplementary Table 12***
///Prevalence (and 95% CI) of widowhood among ever-married men and women aged 65 to 74 in 2016, 2006, and
1999 across states and union territories India///

///NFHS4///
use "/Users/IITMandi/Downloads/Widowhood_NFHS4_5June_2024.dta", clear
// 1 IS MALE //
mean widow [aw=wt] if (hv104==1 & age_grp==3), over(hv024)
// 2 IS FEMALE//
mean widow [aw=wt] if (hv104==2 & age_grp==3), over(hv024)

///NFHS3///
use "/Users/IITMandi/Downloads/Widowhood_NFHS3_5June2024.dta", clear
// 1 IS MALE //
mean widow [aw=wt] if (hv104==1 & age_grp==3), over(hv024)
// 2 IS FEMALE//
mean widow [aw=wt] if (hv104==2 & age_grp==3), over(hv024)

///NFHS2///
use "/Users/IITMandi/Downloads/Widowhood_NFHS2_5June2024.dta", clear
// 1 IS MALE//
mean widow [aw=wt] if (hv104==1 & age_grp==3), over(newstate)
// 2 IS FEMALE//
mean widow [aw=wt] if (hv104==2 & age_grp==3), over(newstate)


***Supplementary Table 13***
///Prevalence (and 95% CI) of widowhood among ever-married men and women aged 45 to 64 in 2016,2006,and 1999
across states and union territories India///

///NFHS4///
use "/Users/IITMandi/Downloads/Widowhood_NFHS4_5June_2024.dta", clear
// 1 IS MALE //
mean widow [aw=wt] if (hv104==1 & age_grp==2), over(hv024)
// 2 IS FEMALE//
mean widow [aw=wt] if (hv104==2 & age_grp==2), over(hv024)

///NFHS3///
use "/Users/IITMandi/Downloads/Widowhood_NFHS3_5June2024.dta", clear
// 1 IS MALE //
mean widow [aw=wt] if (hv104==1 & age_grp==2), over(hv024)
// 2 IS FEMALE//
mean widow [aw=wt] if (hv104==2 & age_grp==2), over(hv024)

///NFHS2///
// 1 IS MALE //
mean widow [aw=wt] if (hv104==1 & age_grp==2), over(newstate)
// 2 IS FEMALE//
mean widow [aw=wt] if (hv104==2 & age_grp==2), over(newstate)

***Supplementary Table 14***
///Prevalence (and 95% CI) of widowhood among ever-married men and women aged less than 45 in 2016, 2006,1999
across states and union territories India///

///NFHS4///
use "/Users/IITMandi/Downloads/Widowhood_NFHS4_5June_2024.dta", clear
// 1 IS MALE //
mean widow [aw=wt] if (hv104==1 & age_grp==1), over(hv024)
// 2 IS FEMALE //
```

```
mean widow [aw=wt] if (hv104==2 & age_grp==1), over(hv024)

///NFHS3///
use "/Users/IITMandi/Downloads/Widowhood_NFHS3_5June2024.dta", clear
// 1 IS MALE //
mean widow [aw=wt] if (hv104==1 & age_grp==1), over(hv024)
// 2 IS FEMALE //
mean widow [aw=wt] if (hv104==2 & age_grp==1), over(hv024)

///NFHS2///
use "/Users/IITMandi/Downloads/Widowhood_NFHS2_5June2024.dta", clear
// 1 IS MALE//
mean widow [aw=wt] if (hv104==1 & age_grp==1), over(newstate)
// 2 IS FEMALE//
mean widow [aw=wt] if (hv104==2 & age_grp==1), over(newstate)
```

End

Text S5. Explanation of authorship change statement

The original authorship contributions statement supplied in the submitted version of this article disclosed that RK and SVS were both involved in the conceptualisation and design of the study, interpretation of the data, critical revision of the manuscript, and overall supervision of the work. However, in the initial submission, RK was inadvertently not recognized as a co-corresponding author with S V Subramanian in the authorship byline. The authors noted this issue at the review stage and notified the Journal of Global Health's editorial team. All authors consented to this change by signing an agreement form.
